# Discovery of Peptidic Siderophore Degradation by Screening Natural Product Profiles in Marine-Derived Bacterial Mono- and Cocultures

**DOI:** 10.1021/acs.biochem.4c00706

**Published:** 2025-01-14

**Authors:** Mónica Monge-Loría, Weimao Zhong, Nadine H. Abrahamse, Stephen Hartter, Neha Garg

**Affiliations:** † School of Chemistry and Biochemistry, Georgia Institute of Technology, 950 Atlantic Drive, Atlanta, Georgia 30332, United States; ‡ Georgia Aquarium, 225 Baker St. NW, Atlanta, Georgia 30313, United States; § Center for Microbial Dynamics and Infection, Georgia Institute of Technology, 315 Ferst Drive, Atlanta, Georgia 30332, United States

## Abstract

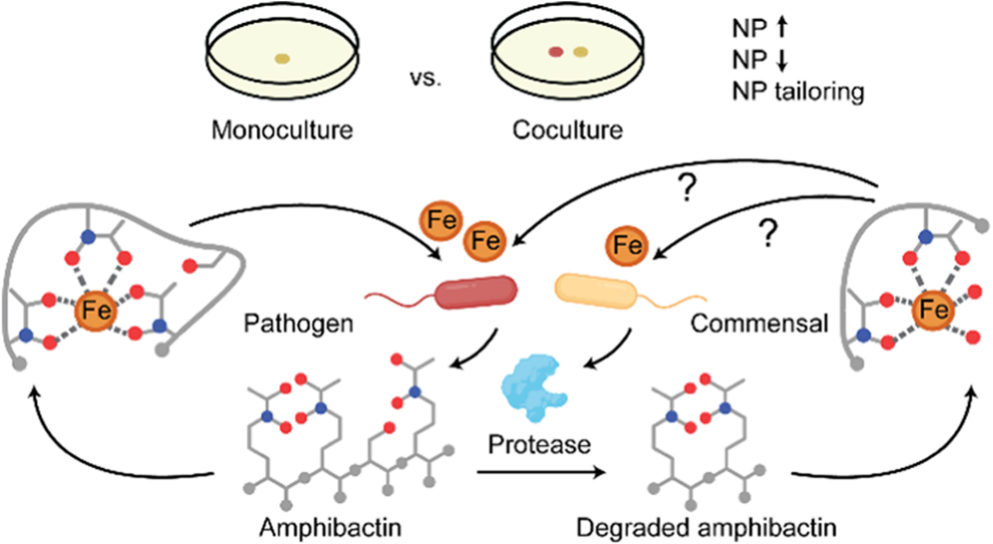

Coral reefs are hotspots of marine biodiversity, which results in the synthesis of a wide variety of compounds with unique molecular scaffolds, and bioactivities, rendering reefs an ecosystem of interest. The chemodiversity stems from the intricate relationships between inhabitants of the reef, as the chemistry produced partakes in intra- and interspecies communication, settlement, nutrient acquisition, and defense. However, the coral reefs are declining at an unprecedented rate due to climate change, pollution, and increased incidence of pathogenic diseases. Among pathogens, *Vibrio* spp. bacteria are key players resulting in high mortality. Thus, alternative strategies such as application of beneficial bacteria isolated from disease-resilient species are being explored to lower the burden of pathogenic species. Here, we apply coculturing of a coral-derived pathogenic species of *Vibrio* and beneficial bacteria and leverage recent advancements in untargeted metabolomics to discover engineerable beneficial traits. By chasing chemical change in coculture, we report *Microbulbifer* spp.-mediated degradation of amphibactins, produced by *Vibrio* spp. bacteria to sequester iron. Additional biochemical experiments revealed that the degradation occurs in the peptide backbone and requires the enzyme fraction of *Microbulbifer*. A reduction in iron affinity is expected due to the loss of one Fe(III) binding moiety. Therefore, we hypothesize that this degradation shapes community behaviors as it pertains to iron acquisition, a limiting nutrient in the marine environment, and survival. Furthermore, *Vibrio* sp. bacteria suppressed natural product synthesis by beneficial bacteria. Understanding biochemical mechanisms behind these interactions will enable engineering probiotic bacteria capable of lowering pathogenic burdens during heat waves and incidence of disease.

## Introduction

The ocean hosts an immense diversity of organisms, particularly coral reefs, which support 25% of all marine life, despite only covering 0.2% of the ocean’s area.^1^ This organismal diversity translates into the biosynthesis of a wide variety of compounds with unique molecular scaffolds, ecological roles, and bioactivities.^2−4^ Bioactive compounds from marine sources are especially useful, as their secretion into the ocean results in dilution necessitating a high potency to elicit a physiological response.^5^ At present, 13 marine-derived drugs have been approved by the Food and Drug Administration (FDA) in the United States, and 32 are at different clinical trial stages,^6^ demonstrating the chemical potential circumscribed to these environments. Interestingly, around a third of these compounds have been isolated from soft-bodied, sessile organisms such as corals and sponges. These characteristics are evolutionary drivers for biosynthetic pathways that produce compounds used for defense, as an advantage against competitors and as predation mechanisms.^7^ Apart from producing these advantageous secondary metabolites, marine macroorganisms have also developed the ability to obtain such compounds from microbes.^7^ In order to do so, they form intricate associations and symbiotic relationships with microbes such as bacteria, fungi, viruses and dinoflagellates; a system collectively referred to as the holobiont.^8,9^ In fact, a myriad of marine natural products previously thought to be produced by eukaryotic organisms have later proven to be of bacterial origin.^10−12^ These complex relationships modulate the macroorganism’s health as well as their response to environmental stressors;^13−17^ therefore, these communities are dynamic in order to select for the fittest holobiont.^18^

Globally, coral reefs are in a precarious state due to increased water temperature, increased incidence of disease, overfishing, hypoxia, and ocean acidification resulting in negative consequences for marine biodiversity, coastal erosion, drug discovery avenues, and human livelihood. Consequently, coral cover has declined by half since the 1950s.^19^ Thus, concentrated efforts are being taken to preserve and restore coral reefs by breeding resilient corals.^20^ Another approach that has seen a recent surge is the use of beneficial bacteria as probiotics to prevent opportunistic infections by pathogens which accelerate tissue damage when temperatures increase during summer.^21−24^ Indeed, the dynamic behavior of coral microbiomes has been linked to resilience of corals against stressors.^25^ The coral probiotic hypothesis proposes that by changing their microbial communities, corals are able to develop resistance to pathogens significantly faster than through mutation and natural selection alone.^18^ Evidence of such defensive symbioses has been observed in sponges, corals, tunicates, mollusks, crustaceans, as well as terrestrial invertebrates and vertebrates.^26,27^ Probiotic bacteria can confer resistance to the host by competitively excluding pathogens, producing antibiotic compounds or a combination of both.^28−30^ Delgadillo-Ordoñez et al. have demonstrated the microbial community shifts in coral reefs upon introduction of probiotic bacteria, alongside the decrease of pathogenic *Vibrio*.^31^ Moreover, Ushijima et al. have shown the use of probiotic bacteria as prophylactic treatment against stony coral tissue loss disease (SCTLD), to slow disease progression.^21^ Additional probiotic strains have been explored to treat SCTLD,^23^ as it has rapidly spread across the coast of Florida and into the Caribbean since its outbreak in 2014, affecting over 20 species of coral and causing the mortality of 30% of corals in Florida.^32^

The opportunistic pathogen, *Vibrio coralliilyticus*, is associated with disease outbreaks in the marine environment affecting various organisms including corals, oysters, and various fish species^33−37^ and is resistant to different classes of antibiotics such as tetracyclines and β-lactams.^38−41^ Although antibiotic application as a topical paste has been successful in coral diseases,^42,43^ it does not present a long-term solution and also raises concern of spreading antibiotic resistance from marine to terrestrial environments. Furthermore, pathogenic species such as *V. coralliilyticus* are part of normal microbiota of corals, but their abundance has been shown to increase during stressors such as increased temperatures during summer and exacerbation of disease acuteness through coinfections.^44^ Thus, introducing beneficial bacteria isolated from the marine environment serves as a useful and safe approach to reduce the pathogenic burden. The coral reef ecosystem presents a source of a very rich microbial consortium whose components interact in complex ways and microbial repositories are being created and exploited to identify beneficial traits.^23,24^ To leverage the coral probiotic hypothesis in the search for beneficial bacterial traits, we isolated and obtained several coral or sponge-derived microbes and cocultured them with a coral-derived strain of *V. coralliilyticus* (Cn52-H1), capable of producing andrimid, toxins, and additional virulence factors.^39^ Furthermore, we cultured strains that we have shown as prolific producers of natural products, previously isolated from corals.

In the marine environment, literature surveys have identified Actinobacteria, Firmicutes and Proteobacteria as the main phyla linked to antimicrobial activity and natural product biosynthesis^4^ among marine bacteria. Proteobacteria are the most abundant and diverse phylum^45^ in the ocean, collectively constituting over 50% of bacteria. Proteobacteria are additionally one of the major protease-producing phyla.^46−50^ This phylum includes the genus *Pseudoalteromonas*, which is known to devote up to 15% of its genome to secondary metabolite production.^51^ This genomic distribution is on par with known prolific secondary metabolite producers such as *Streptomyces*.^52^ Apart from pigments such as violacein, and pyomelanin, *Pseudoalteromonas* produce bioactive compounds including thiomarinol, macrolactins and bromoalterochromides and bioactive pigments such as prodigiosin.^4,53^ Another genus belonging to this phylum is *Microbulbifer*, a genus that has been recognized for its enzymatic capabilities^54−58^ and that has recently sparked interest due to its natural product potential. Secondary metabolites such as bulbiferates,^59^ bulbiferamides^60,61^ and pseudobilbiferamides^62^ have been described, however, the gap between identified natural products and the number of BGCs in their genomes exhibits the potential for natural product discovery from *Microbulbifer*.^63^ Thus, we ensured that representatives of the genera *Pseudoalteromonas* and *Microbulbifer* were included as coculture partners with *V. coralliilyticus* Cn52-H1. Using comparative untargeted metabolomics, coupled with the introduction of a pathogenic coculture partner, we aimed to identify potential beneficial traits of marine-derived bacteria against the pathogen. Using this approach, we discovered the ability of *Microbulbifer* spp. bacteria to enzymatically degrade a peptidic natural product, amphibactin, a siderophore produced by *V. coralliilyticus* Cn52-H1 reducing its affinity for iron. The biological function of amphibactins is to acquire iron from the surrounding environment, which is a scarce commodity for microbes in dilute ocean environments and is essential for microbial growth.^64,65^ Notably, amphibactins were found to be one of the most abundant siderophores in the open ocean highlighting its ecological importance.^65^ We observe that the degradation of amphibactin is carried out in the peptide backbone by multiple strains, which is the first report of such a hydrolysis of peptidic siderophores further enhancing the ecological implication of our finding. In retaliation to the presence of beneficial bacteria, *V. coralliilyticus* Cn52-H1 suppressed natural product biosynthesis by the beneficial bacteria. Thus, our work highlights complex interplay of biochemical interactions between marine derived pathogenic and beneficial bacteria.

## Results and Discussion

### Comparative Metabolomics of Bacterial Mono- and Cocultures with *V. coralliilyticus* Cn52-H1

**Figure 1:**
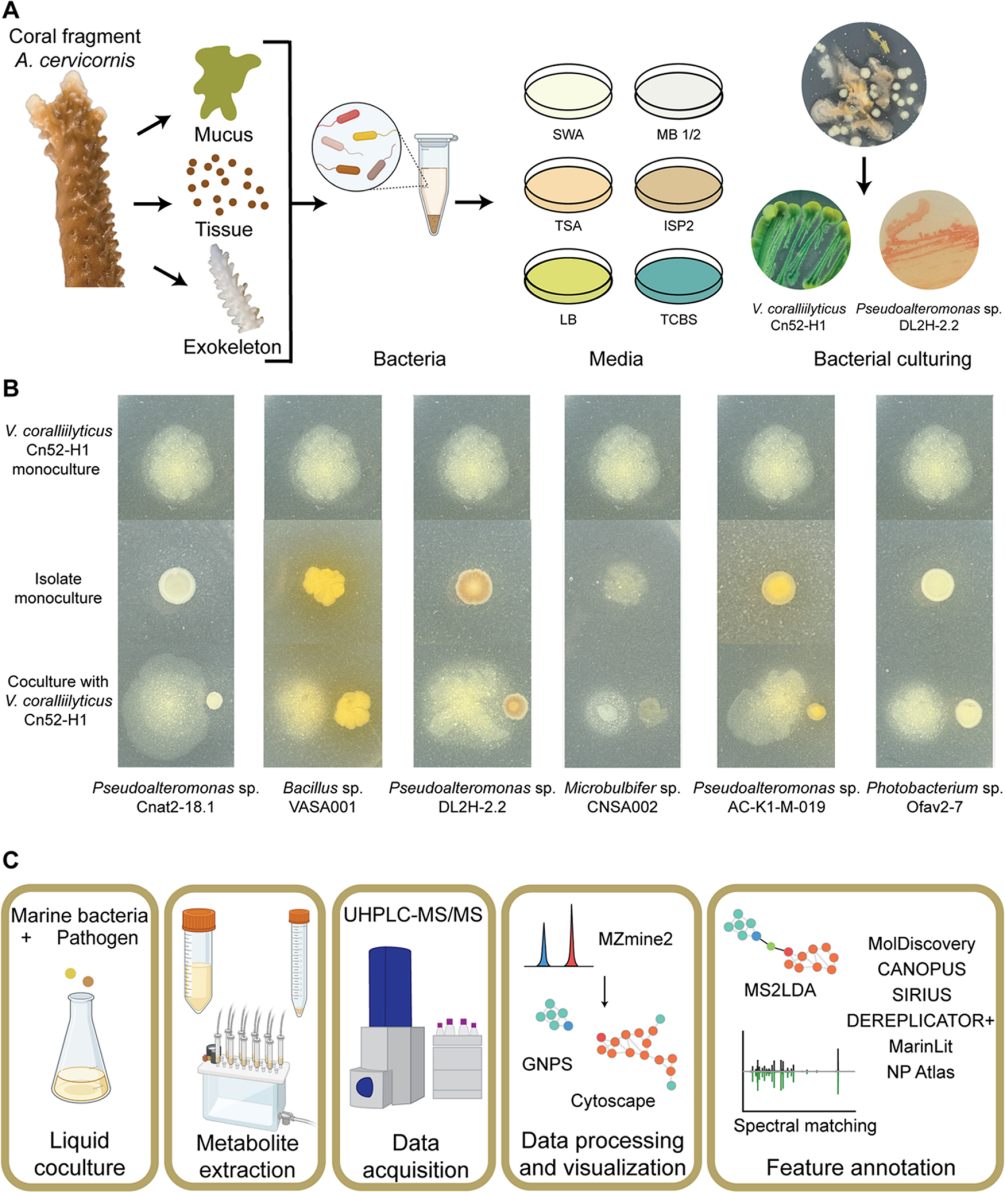
Bacterial isolation and metabolomics workflow. (A) Bacterial isolation from coral mucus, tissue, and the remaining skeleton was performed on six different culture media. Morphologically distinct colonies were isolated and restreaked to ensure purity. (B) Prioritized isolates’ phenotype in mono- and coculture with *V. coralliilyticus* Cn52-H1. (C) Prioritized strains were cocultured with *V. coralliilyticus* Cn52-H1, metabolites were extracted using liquid–liquid extraction (LLE), solid phase extraction (SPE), and solid–liquid extraction (SLE). The extracts were analyzed using UPLC-MS and the data was processed for downstream analysis using a suite of cheminformatics tools for compound annotation.

A total of 210 bacterial strains either isolated from *Acropora cervicornis* coral (119 isolates, sourced from Georgia Aquarium, Figure 1A) or sourced from previous isolations^23,66^ were cocultured with *V. coralliilyticus* Cn52-H1, previously isolated from healthy corals.^23^ This *Vibrio* isolate was selected as a coculture partner since we previously showed production of andrimid by this strain,^23^ a known secondary metabolite elicitor^67^ and due to the implicated role of *V. coralliilyticus* as a member of pathogenic consortia in the marine environment. The bacterial strains that altered colony morphology of *V. coralliilyticus* Cn52-H1 were prioritized for liquid culturing (Figure 1B and Table 1). Three extraction methods were performed to capture a wider breadth of metabolites (Figure 1C): liquid–liquid extraction (LLE), solid phase extraction (SPE), and solid–liquid extraction (SLE). Metabolomics data on these extracts were acquired via ultrahigh-performance liquid chromatography (UHPLC) coupled to high-resolution mass spectrometry (MS) in the positive ionization mode using data-dependent acquisition. Chromatographic peaks were extracted using MZmine2^68^ which provides a feature list consisting of *m*/*z*, retention time, and area under the chromatographic peak for each metabolite feature. The metabolites detected in blank and media controls were subtracted from this output resulting in a total of 5349 high quality metabolite features along with a consensus MS^2^ spectra for each feature. This data was further analyzed to compare the metabolite profiles of mono- and cocultures as summarized in Figure 1C and described in detail below.

**Table 1:** Strains Prioritized for Coculture with *V. coralliilyticus* Cn52-H1

strain	genus	origin	references
Cnat2–18.1	*Pseudoalteromonas*	Coral; *Colpophyllia natans*, Atlantic Ocean	(23)
DL2H-2.2	*Pseudoalteromonas*	Coral; *Diploria labyrinthiformis*, Atlantic Ocean	(23)
Ofav2–7	*Photobacterium*	Coral; *Orbicella faveolata*, Atlantic Ocean	(23)
AC-K1-M-019	*Pseudoalteromonas*	Coral; *A. cervicornis*, Georgia Aquarium	this work
CNSA002	*Microbulbifer*	Sponge; *Smenospongia aurea,* Atlantic Ocean	(66)
VASA001	*Bacillus*	Sponge; *S. aurea*, Atlantic Ocean	(66)

**Figure 2:**
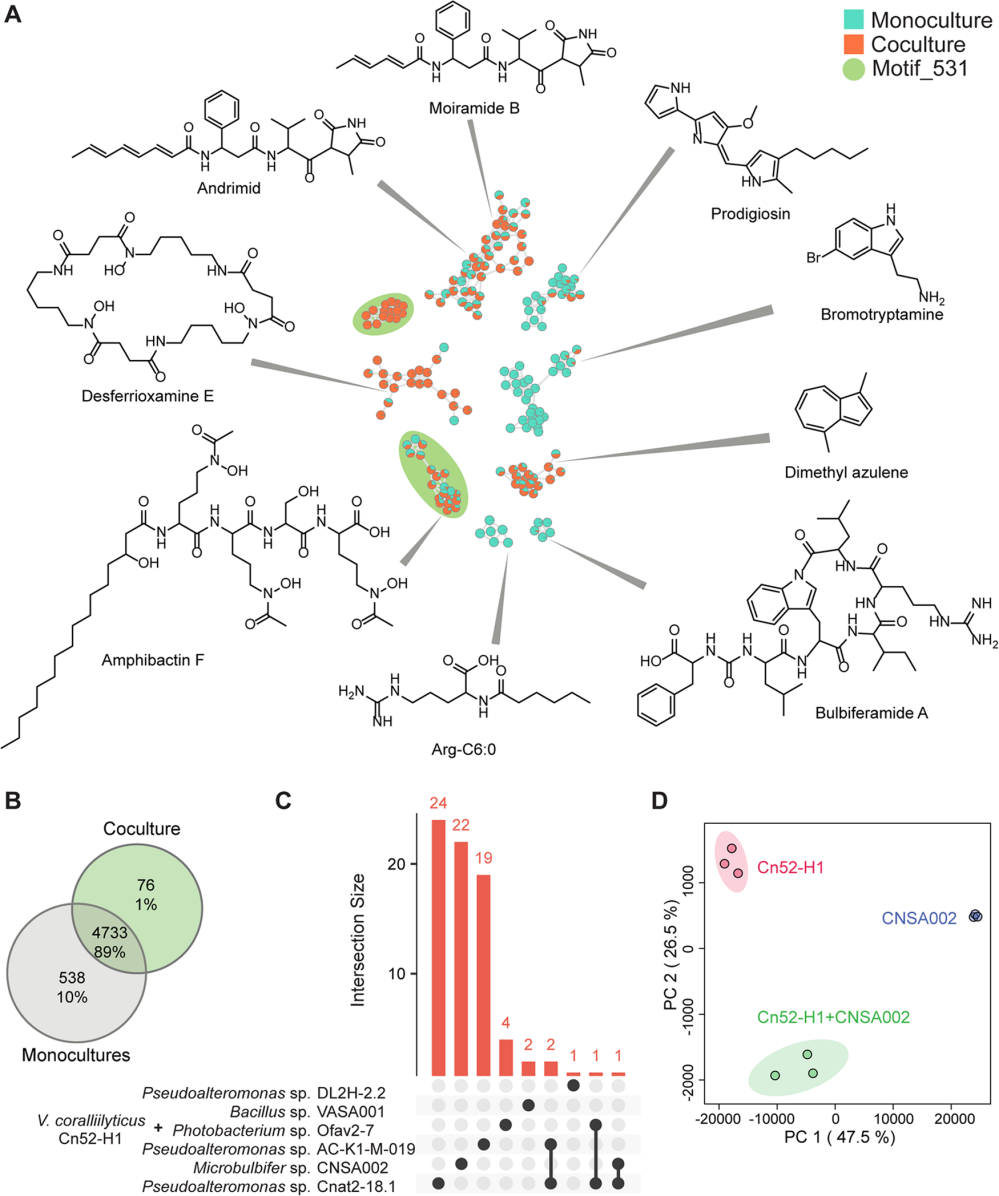
Metabolome profiling of bacterial mono- and cocultures. (A) FBMN showcasing a subset of features that were annotated in this study and their representative chemical structures. The amphibactin cluster and a cluster of unknown metabolites detected in coculture with *Microbulbifer* sp. CNSA0002 and sharing the MS2LDA motif 531 with the amphibactin cluster are highlighted with a green circle. (B) A Venn diagram representation of the number of features detected across different culture conditions. (C) An UpSet plot is used to show the distribution of 76 features unique to cocultures. (D) PCA plot of untargeted metabolomics data acquired on extracts of *V. coralliilyticus* Cn52-H1 monoculture, *Microbulbifer* sp. CNSA002 monoculture and their coculture.

The feature list and consensus MS^2^ spectra was first used to generate a feature-based molecular network (FBMN) in the global natural products social molecular networking (GNPS)^69^ platform, which was visualized in Cytoscape^70^ (Figures 2A and S1). A node in the network represents a unique *m*/*z* and chromatographic retention time (referred to as metabolite feature in this manuscript). The network is generated by quantifying MS^2^ spectral similarity and consists of either singleton nodes (MS^2^ spectrum having no similarity with other MS^2^ spectra in the data set) or several clusters of connected nodes (similar MS^2^ spectra). Similarity in chemical structures result in similar MS^2^ spectra, hence, the connected nodes represent structurally related molecules. In this network, 538 nodes (10.1% of total) were detected exclusively in extracts of monocultures, 76 nodes (1.5%) in extracts of cocultures, and 4733 (88.5%) were shared across all culture extracts (Figure 2B). An UpSet plot was generated in the Intervene platform^71^ to visualize the distribution of the 76 features detected across different coculture extracts (Figure 2C). Notably, the cocultures between *V. coralliilyticus* Cn52-H1 and *Pseudoalteromonas* sp. Cnat2–18.1, *Microbulbifer* sp. CNSA002, and *Pseudoalteromonas* sp. AC-K1-M-019 had the highest number of unique features, with 24, 22, and 19 unique features, respectively. The 15 of 22 features detected in coculture of *Microbulbifer* sp. CNSA002 clustered together representing structurally related compounds that are exclusively detected in coculture. Thus, this coculture combination was further prioritized for analysis including principal component analysis (PCA, Figure 2D) and heat map generated using hierarchical cluster analysis (HCA, Figure S2). The PCA plot revealed a clear separation between mono- and coculture, with principal components PC1 and PC2 accounting for 74% of variance. The heatmap supports the observation that the metabolome of each group is distinct and reveals more features that display variable detection between mono- and coculture extracts.

### Annotation of Features Variably Detected in CNSA002 Coculture

**Figure 3:**
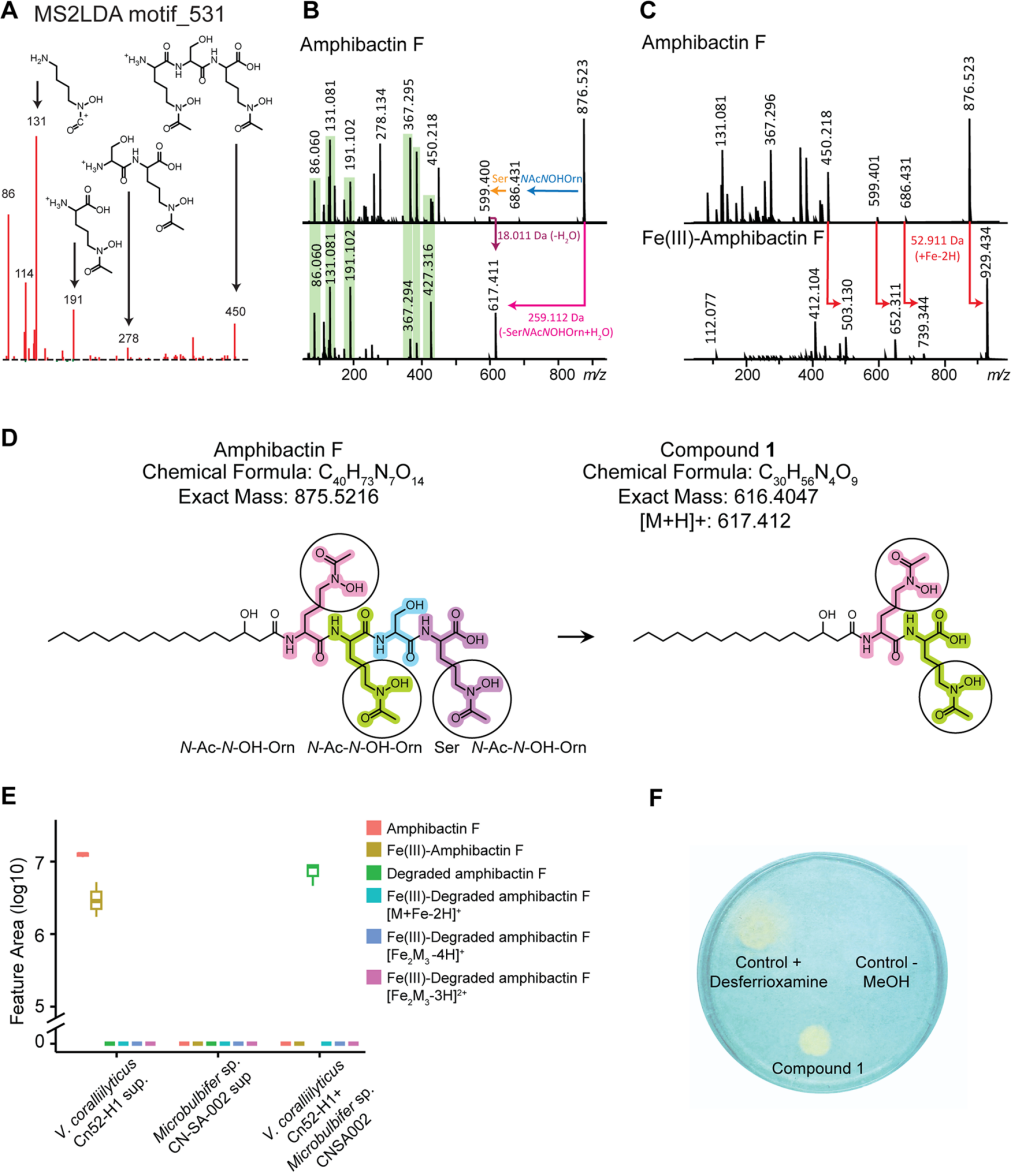
Amphibactin degradation by *Microbulbifer* sp. CNSA002. (A) MS2LDA motif 531, annotated as containing *N*-acetyl-*N*-hydroxy-ornithine. (B) Spectral comparison of amphibactin F and unknown feature with *m*/*z* 617.411 produced only in coculture. (C) MS^2^ mirror plot of amphibactin F and Fe(III)-amphibactin F. No Fe(III)-bound complex of *m*/*z* 617.411 was observed. (D) Amphibactin F, produced by *V. coralliilyticus* Cn52-H1 is degraded in the presence of *Microbulbifer* sp. CNSA002 cell-free supernatant producing compound **1**, structurally elucidated through NMR (Table S4 and Figures S4–S12). Iron-binding hydroxamate moieties are circled. (E) Boxplots of the relative abundances of amphibactin F and its degradation product in their apo- and complex form in monoculture and coculture. (F) Petri plate showing iron chelating activity of purified compound **1** using O-CAS agar assay.

**Figure 4:**
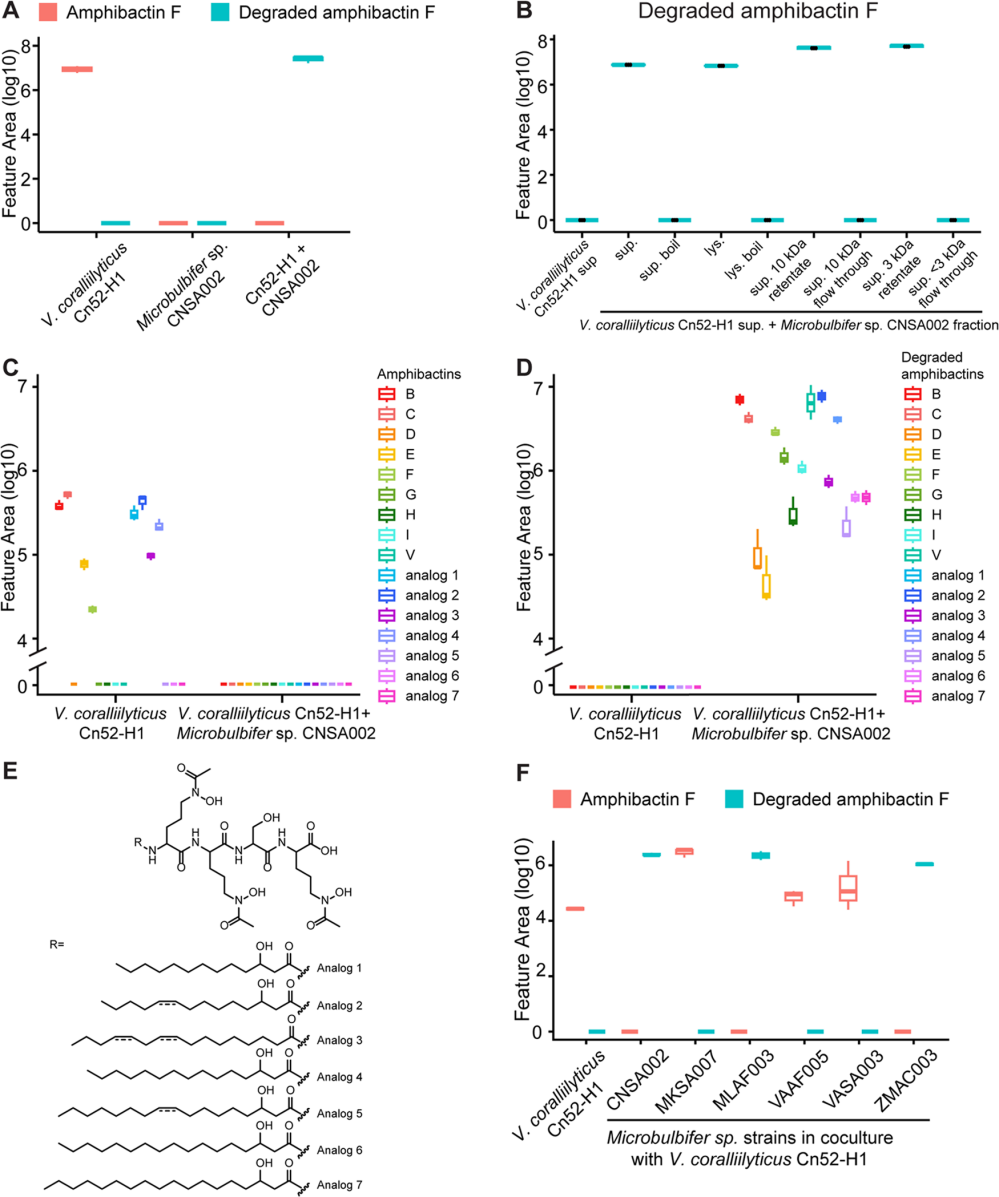
Detection of amphibactins and their degradation products. (A) Boxplots of the relative abundances of amphibactin F (876.529 *m*/*z*) and its degradation product (617.412 *m*/*z*) in monoculture of *V. coralliilyticus* Cn52-H1, *Microbulbifer sp*. CNSA002, their coculture, and Cn52-H1 culture in the presence of cell-free supernatant of CNSA002. (B) Boxplots of relative abundances of degraded amphibactin F in *V. coralliilyticus* Cn52-H1 cell-free supernatant alone, after boiling, and in the presence of flow through or retentate of an ultrafiltration experiment with either a 3 kDa or 10 kDa membrane. (C) Boxplots of amphibactins and (D) their degradation products in *V. coralliilyticus* Cn52-H1 monoculture and its coculture with *Microbulbifer sp*. CNSA002. (E) New amphibactin analogs indirectly identified through their degradation products in *Microbulbifer* sp. CNSA002 and *V. coralliilyticus* Cn52-H1 cocultures. The position of double bond was not determined and is putatively placed (shown with dashed line). (F) Boxplots of the relative abundances of amphibactin F and its degradation product in *V. coralliilyticus* Cn52-H1 monoculture and in coculture with several *Microbulbifer* sp. strains.

Several approaches were applied to identify the metabolite features, production of which was observed to be variable in the PCA, HCA (Table S1) and UpSet plot analysis (Table S2). First, the FBMN was queried to identify whether MS^2^ spectral matching with the GNPS library resulted in a match. These matches were further analyzed for accuracy of annotation. Second, the MS^2^ spectra of metabolite features with no spectral matches in the GNPS library were analyzed using a suite of cheminformatics tools including Sirius,^72^ CANOPUS^73^ with CSI:FingerID,^72^ and MS2LDA^74^ (see Methods Section) and multiple natural product databases such as the Natural Product Atlas,^75^ and MarinLit^76^ were manually searched. Several natural products were annotated using this pipeline (Table S3), relevance of which is described in the following subsections. However, the cluster of 15 nodes of interest detected exclusively in coculture of *V. coralliilyticus* Cn52-H1 and *Microbulbifer* sp. CNSA002 (orange nodes highlighted with a green circle, Figure 2A) had no spectral matches in the GNPS spectral library and no reasonable annotations were obtained by manual searching of the spectral or compound databases. Thus, we resorted to *in silico* methods for annotation before the isolation and structural characterization via NMR was attempted. The MS2LDA analysis, an unsupervised substructure discovery method, which outputs a set of common fragment ions and neutral losses in the MS^2^ spectra was applied. The features of interest contained the substructure motif 531, also present in the nodes annotated as amphibactins. The characteristic peak at 191.102 *m*/*z*, and its related fragments (Figure 3A,B), lead us to hypothesize the presence of an *N*-acetyl-*N*-hydroxy-ornithine amino acid as a substructure in these nodes of interest. This modified, noncanonical amino acid is ubiquitous in siderophores as the hydroxamate moiety is a strong iron chelator.^77^ The comparison of MS^2^ spectra of the unknown feature at *m*/*z* 617.411 and apo-amphibactin F further supported this observation (Figure 3B). We searched for an analyte corresponding to *m*/*z* of Fe(III)-amphibactin complex and Fe(III) complex of feature with *m*/*z* 617.411, but only observed Fe(III)-bound amphibactin (Figure 3C,E). Interestingly, concomitant with the exclusive detection of the unannotated features in the coculture, amphibactins themselves were not detected in coculture (Figures 3E, and 4A). This observation further substantiates the developing theory that these features likely originate from *Microbulbifer* sp. CNSA002 mediated degradation of amphibactins (Figure 3D). Additionally, since no features from *Microbulbifer* sp. CNSA002 monoculture were linked to this MS2LDA motif, we concluded that this biotransformation occurs on the amphibactin produced by *V. coralliilyticus* Cn52-H1, which was further verified by isolation and structural characterization.

**Figure 5:**
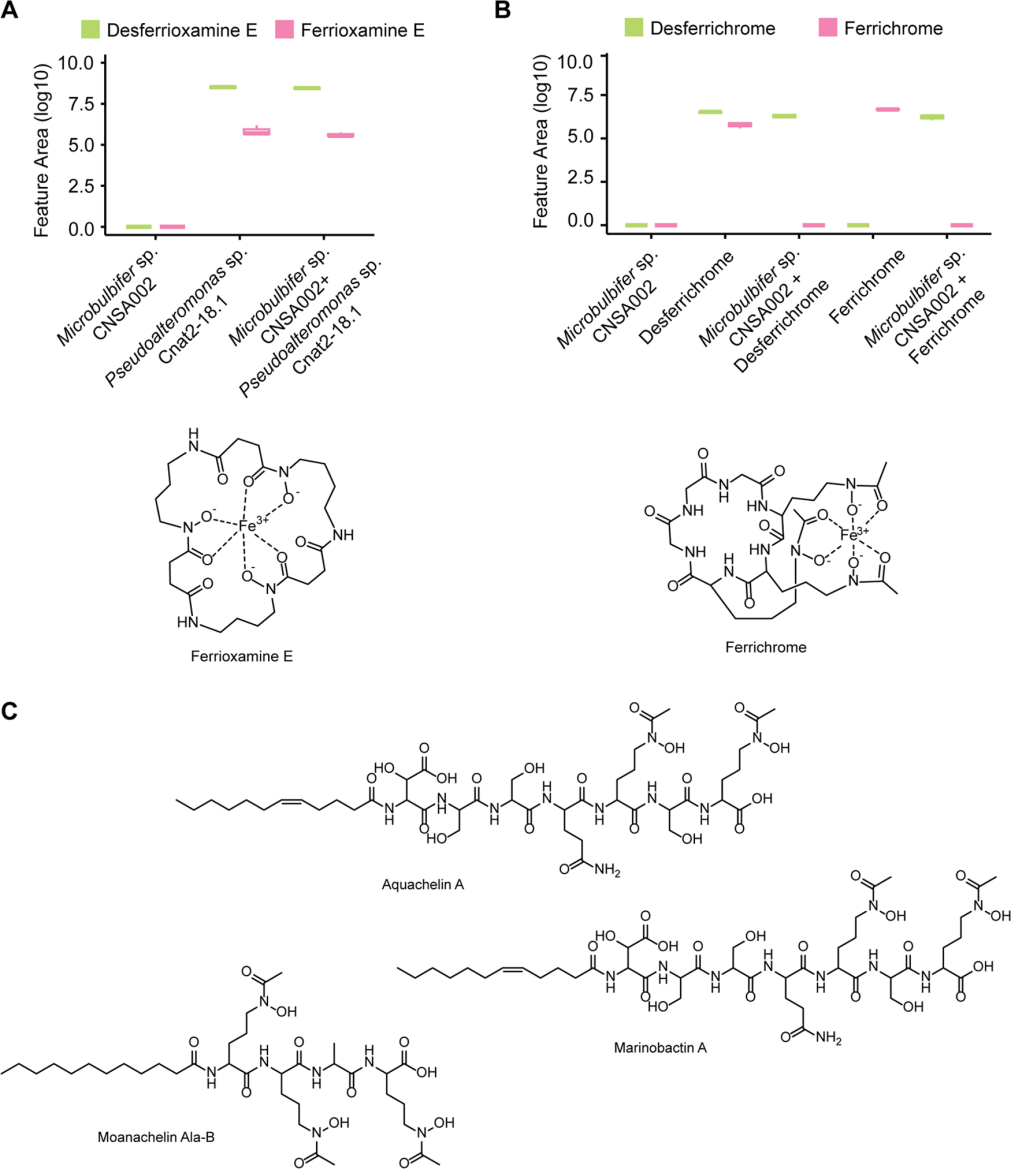
Detection of hydroxamate siderophores and ferrisiderophores in the presence of *Microbulbifer* sp. CNSA002. (A) Boxplot of the relative abundances of desferrioxamine E and ferrioxamine E (601.356 *m*/*z* and 654.267 *m*/*z* respectively) in *Pseudoalteromonas* sp. Cnat2–18.1 and *Microbulbifer* sp. CNSA002 mono- and coculture. (B) Boxplot of the relative abundances of desferrichrome and ferrichrome (688.326 *m*/*z* and 741.237 *m*/*z* respectively) when supplemented in a *Microbulbifer* sp. CNSA002 culture and controls. (C) Representative structures of peptidic hydroxamate siderophores: aquachelin, marinobactin, and moanachelin.

The *V. coralliilyticus* Cn52-H1 and *Microbulbifer* sp. CNSA002 coculture was extracted and fractionated using SPE, followed by HPLC (Figure S3). The fractions were analyzed by liquid chromatography–mass spectrometry (LC–MS) to determine the composition of each fraction. The fraction with the highest purity was characterized through one-dimensional (1D) and two-dimensional (2D) NMR validating the proposed structure of unknown feature at *m*/*z* 617.411, referred to as compound **1** from hereon (Figures S4–S12 and Table S4). Notably, the difference between this structure and amphibactin F lies in the absence of a serine and an *N*-acetyl-*N*-hydroxy-ornithine unit (Figure 3D). The loss of these two amino acids are commensurate with a Δ*m*/*z* of 259.112 between the amphibactin and the respective amphibactin degradation product (Figure 5B). In this way, we were able to assign the putative chemical structures to the 15 nodes exclusively present in coculture and the corresponding parent amphibactin molecule (Figures S13, S14 and Table 2). This assignment was additionally confirmed through the compounds’ fragmentation patterns (Figures S13 and S14).

**Table 2:** Degraded Amphibactins Detected in *V. coralliilyticus* Cn52-H1 and *Microbulbifer* sp. CNSA002 Coculture

name	theoretical *m*/*z* [M + H]^+^	experimental *m*/*z* [M + H]^+^	error (ppm)	corresponding amphibactin	theoretical *m/z* [M + H]^+^	acyl tail	references
degraded amphibactin D	573.386	573.386	0	Amphibactin D	832.5026	C14:0	(78)
degraded amphibactin V	587.401	587.401	0	Amphibactin V	846.5183	C15:0	(79)
degraded amphibactin B	589.381	589.381	0	Amphibactin B	848.4975	C14:0;3-OH	(78)
degraded amphibactin E	599.401	599.402	1.7	Amphibactin E	858.5183	C16:1	(78)
degraded amphibactin H	601.417	601.417	0	Amphibactin H	860.5339	C16:0	(78)
degraded amphibactin C	615.396	615.397	1.6	Amphibactin C	874.5132	C16:1;3-OH	(78)
degraded amphibactin F	617.412	617.412	0	Amphibactin F	876.5288	C16:0;3-OH	(78)
degraded amphibactin I	627.433	627.433	0	Amphibactin I	886.5496	C18:1	(78)
degraded amphibactin G	643.428	643.427	1.6	Amphibactin G	902.5445	C18:1;3-OH	(78)
degraded amphibactin analog 1	575.365	575.365	0	Analog 1	834.4819	C13:0;3-OH	this work
degraded amphibactin analog 2	587.365	587.366	1.7	Analog 2	846.4819	C14:1^a^;3-OH	this work
degraded amphibactin analog 3	597.386	597.386	0	Analog 3	856.5026	C16:2^a^	this work
degraded amphibactin analog 4	603.396	603.397	1.7	Analog 4	862.5132	C15:0;3-OH	this work
degraded amphibactin analog 5	629.412	629.412	0	Analog 5	888.5284	C17:1^a^;3-OH	this work
degraded amphibactin analog 6	631.428	631.427	1.6	Analog 6	890.5437	C17:0;3-OH	this work
degraded amphibactin analog 7	645.443	645.443	0	Analog 7	904.5599	C18:0;3-OH	this work

a Position of double bond was not determined.

To test for the ability of compound **1** to bind Fe(III), we performed a modified chrome azurol S (CAS) assay (O-CAS assay^80^). The positive control, desferrioxamine mesylate, and compound **1** showed a color change from blue to orange (Figure 3F). This observation suggests that the degraded amphibactin can bind iron. This observation is expected as compound **1** is still a tetradentate hydroxamate ligand. However, a decrease in binding affinity is expected due to the loss of a hydroxamate moiety. Furthermore, we did not detect the Fe-bound form of compound **1** while Fe(III)-bound amphibactin was detected (Figure 3C, Δ*m*/*z* of 52.9115, [M + Fe-2H]^+^ adduct), suggesting that other siderophores or proteins produced by *Microbulbifer* sp. can steal iron from the degraded amphibactin form. Indeed, *Microbulbifer* sp. does have the ability to acquire iron as shown by the O-CAS assay (Figure S15). The purported lower iron affinity can be explained by Fe(III)’s preference for octahedral geometry, maximizing iron affinity when ligands are hexadentate.^81^ However, tetra- and bidentate siderophores can also form a complex with Fe(III) and support bacterial growth, but have lower binding affinity.^81−83^ Examples of these siderophores include 2,3-dihydroxybenzoylglycine, amonabactins, rhodotorulic acid, alcaligin and bisucaberin.^82,84−86^ Tetradentate, dihydroxamate siderophores have also been found to form different Fe(III) complexes at different pH, for instance Fe_2_L_3_ complexes (where LH_2_ represents the ligand), in order to complete the coordination for iron.^87^ This complex form including singly or doubly charged adducts of the amphibactin degradation products were manually searched in the data set and were also not detected under our experimental conditions further supporting that *Microbulbifer* degrades amphibactin to gain competitive advantage for iron acquisition.

### Enzymatic Degradation of Amphibactins and New Amphibactin Analogs

One drawback of single-vessel cocultures is the difficulty in assigning the biological source of the molecule to either bacteria. To overcome this challenge and validate that *Microbulbifer* sp. is required for the detection of compound **1**, we cultured *V. coralliilyticus* Cn52-H1 in the presence of cell-free supernatant of *Microbulbifer* sp. CNSA002. Cell-free supernatant controls of monocultures were incubated with filtered artificial seawater (FASW) alongside and treated in the same manner. Metabolites were extracted and data were acquired following the workflow in Figure 1C. The amphibactin degradation products were not detected in the cell-free supernatant of *V. coralliilyticus* Cn52-H1, which indicated that the degradation is not a time-, temperature- or light-dependent transformation (Figures 4A and S16). Additionally, no change in pH was observed upon coculture. The amphibactin degradation products were detected in coculture (Figure 4A, Cn52-H1 + CNSA002). To explore if the degradation of amphibactin is enzymatic, we boiled both the cell-free supernatant and the lysed cell pellet of CNSA002 and incubated it with *V. coralliilyticus* Cn52-H1 cell-free supernatant containing amphibactins. No degradation was observed after boiling (Figures 4B and S17). Additionally, we also fractionated the *Microbulbifer* sp. CNSA002 supernatant using 3 and 10 kDa ultracentrifugation filters to separate small molecules from macromolecules. The degradation was observed only with the retentate of either filter and no degradation was observed with the flow through of either filter (Figures 4B and S17). Therefore, we propose that the amphibactin degradation is carried out by a protease produced by *Microbulbifer* sp. CNSA002.

All analogs of amphibactin are degraded by *Microbulbifer* sp. CNSA002 (Figure 4C,D), and the degraded compounds are generally detected at a higher intensity than the corresponding amphibactins. Some amphibactins (D, G, H, I, V, analog 5, 6 and 7) were not detected in the *V. coralliilyticus* Cn52-H1 monoculture (Figure 4C), but concurrent with their increased production in coculture, their degradation products were detected in coculture with *Microbulbifer* sp. CNSA002 (Figure 4D). This observation, in conjunction with FBMN annotation propagation and MS^2^ analysis, allowed for the indirect determination of seven amphibactin analogs that had not been previously reported (Figure 4E). These analogs differ in acyl chain length, six of the seven contain a hydroxylation, and one of the analogs is doubly saturated (Table 2). The membrane partitioning of amphibactins is previously reported to be higher for longer chain acyl-siderophores when compared with shorter chain, unsaturated, and hydroxylated fatty acids.^88^ Additionally, coculturing often results in shifts in membrane composition and fatty acid profiles of microorganisms. Thus, variable fatty acid precursors may be available in coculture for acylation of the amphibactin headgroup. We surveyed five additional *Microbulbifer* sp. strains from our library to determine whether amphibactin degradation is a conserved trait across different marine *Microbulbifer* sp. bacteria. Among the tested bacteria, the strains MLAF003 and ZMAC003 degraded amphibactin, whereas MKSA007, VAAF005, and VASA003 did not. However, genomic analysis of the amphibactin-degrading and nondegrading strains did not yield a candidate protease following this pattern, suggesting that some *Microbulbifer* sp. are nondegrading due to the enzyme either not being present or not being expressed under the conditions tested (Figure 4F).

Iron, being an essential trace nutrient, is acquired by microorganisms from the external environment as Fe(III) via siderophores, especially in marine environments where iron availability is limited. Thus, high-affinity siderophore production and iron acquisition lie at the heart of mutualistic and competitive interactions between organisms. Because of high iron affinity, Fe(III)-siderophore complexes are stable, therefore, iron release from these complexes requires specialized strategies. These include Fe(III) reduction to Fe(II), change in coordination mode, proton assisted dissociation and siderophore degradation via esterases.^81,89−91^ Esterase-mediated iron release has been described for the tris-catechol siderophore enterobactin and its glycosylated analogs, salmochelins, as early as 1971.^92−95^ A similar degradation is observed in bacillibactin in *Bacillus subtilis*(96,97) and in *Aspergillus* fungi for fusarinines.^98−100^ Although fusarinines and amphibactins are both hydroxamate siderophores, fusarinines contain an ester bond whereas amphibactins contain amide bonds. Therefore, the enzymatic degradation of fusarinines is carried out by esterases, unlike the proteolytic degradation proposed herein. Bacterial amidases have been shown to degrade the hydroxamate siderophore family of desferrioxamines, however, this degradation did not occur on the holo-siderophore (ferrioxamines), and the degraded product was used as a carbon and nitrogen source rather than for iron acquisition.^101−106^ Furthermore, the degradation in these cases does not involve the peptide backbone amide bond. Nonenzymatic desferrioxamine degradation has been reported by the fungus *Pyrenophora biseptata*, involving the reduction of the hydroxamate moieties.^107^ This degradation was proposed to lower the iron affinity of the desferrioxamine, as the chelating moieties are lost, and therefore increase iron availability to other microbes and plants. However, no degradation was observed for the ferri-siderophore suggesting that iron chelation protected the molecule from degradation. Another hydroxamate siderophore, ferrichrome, is degraded by *Pseudomonas*, again as a carbon and nitrogen source.^108−110^ Siderophore modification as an iron release strategy has only been observed in catecholate-type siderophores. Catecholate-type siderophores possess the highest binding affinities and the bound Fe(III) exhibits an extremely low redox potential, necessitating this alternative method for iron release from the ferri-siderophore.^91^ In contrast, hydroxamate siderophores typically utilize a reductive mechanism.^111^ This redox mechanism for iron release has been proposed for amphibactins, as it is energetically favorable.^112^ Nonetheless, the hydrolysis of a hexadentate hydroxamate siderophore to a tetradentate one shifts the Fe(III) redox potential, favoring its reduction into the bioavailable Fe(II) form and its dissociation from the complex.^113^

Siderophore tailoring has been observed for other amphiphilic hydroxamate siderophores, where cleavage of the acyl tail by an amide hydrolase produces the siderophore headgroup.^114^ This degradation was carried out by a *Marinobacter* sp. bacterium on a variety of marine siderophores including the native marinobactins, and the xenosiderophores aquachelins, and loihichelins.^114^ This hydrolysis is evidently different from the reaction described in this work, as the chelating groups remain unchanged. As such, the purpose for this enzymatic reaction is proposed to be related to siderophore availability and diffusion, since the acyl tail of this class of amphiphilic siderophores has been linked to membrane partitioning as a way to counter diffusion in the marine environment.^88,111^ Nonetheless, this study constitutes another instance of siderophore biotransformation in bacterial cocultures revealing a possible common strategy in iron acquisition. Thus, to the best of our knowledge, this work is the first report of a hydroxamate degradation that involves hydrolysis of the peptide backbone.

One drawback of the siderophore-degradation strategy for iron release is the energetic strain for the bacteria, since it prevents the recycling of the siderophore. However, this disadvantage is circumvented if the siderophore-degrading bacteria is not the producer of the compound. This strategy has been observed in the enterobactin degradation by *Pseudomonas aeruginosa*, *Campylobacter jejuni* and *Campylobacter coli*,^115,116^ all of which do not produce this compound but rather use it as a xenosiderophore. Thus, degradation of amphibactins by *Microbulbifer* sp. CNSA002 might serve two purposes. First, it lowers the affinity for iron so a stronger siderophore can steal it. The *Microbulbifer* sp. CNSA002 does have the genomic potential to produce two siderophores, a pyochelin-type siderophore and a predicted nonribosomal peptide synthetase-independent siderophore (Table S5 and Figure S15). Furthermore, genomic analysis revealed that CNSA002 has a TonB-dependent transporter^117^ for siderophores, bacterial iron storage protein bacterioferritin,^118^ and ferrichrome iron receptor involved in the uptake of iron in complex with ferrichrome. Second, *Microbulbifer* sp. may be able to import the degraded siderophore, which remains to be explored in future work.

### Specificity of Siderophore Degradation

The hydroxamate moiety is found in several classes of marine derived siderophores including ferrioxamine, ferrichrome, marinobactins, moanachelins, acquachelins, acremonpeptides, and tenacibactins, to name a few.^77^ To determine the specificity of siderophore degradation exhibited by *Microbulbifer* sp. CNSA002 we cocultured it with the desferrioxamine-producing *Pseudoalteromonas* sp. Cnat2–18.1 from our bacterial culture library. No degradation of apo- or Fe(III)-bound form of desferrioxamine was observed in coculture, as the levels detected in mono- and coculture were not significantly different (Figures 5A and S18). Similar observations were made when the media was supplemented with analytical commercially available standard of desferrioxamine B. Next, the possibility of ferrichrome degradation was also explored, by supplementation of *Microbulbifer* sp. CNSA002 culture with the siderophore in both Fe(III)- bound and unbound form. The controls where media is incubated with ferrichrome (Figure 5B, Ferrichrome) or desferrichrome (Figure 5B, Desferrichrome) showed that the compound was stable in the media conditions. No evident degradation was observed for either siderophore form in the presence of *Microbulbifer* sp. CNSA002. However, no ferrichrome was detected when the *Microbulbifer* sp. CNSA002 was incubated with ferrichrome (Figure 5B, *Microbulbifer* sp. CNSA002 + Ferrichrome). Instead, only non-Fe(III) bound form, desferrichrome, was detected. This is in contrast to the siderophore detection for ferrioxamines, where the detection of Fe(III)-bound form is not significantly different between the *Pseudoalteromonas* sp. monoculture and its coculture with *Microbulbifer* sp. CNSA002. This observation suggests that *Microbulbifer* sp. strain CNSA002 can acquire iron from ferrichrome but not from ferrioxamine. Indeed, a ferrichrome iron receptor to import ferrichrome inside the cell was found in the genome of *Microbulbifer* sp. CNSA002.

The inability of *Microbulbifer* sp. CNSA002 to degrade desferrioxamines and ferrichromes is not surprising, as these siderophores are purposefully selected to be nonpeptidic and cyclic, respectively.^101^ We were unable to test the degradation of aquachelins, moanachelins, and marinobactins (Figure 5C), which are peptidic siderophores structurally similar to amphibactins due to nonavailability of producing bacteria. However, the possibility of *Microbulbifer* sp. CNSA002 to use both amphibactins and ferrichrome as xenosiderophores evidence the complex interactions in bacterial communities surrounding iron bioavailability. Since siderophores are secreted, they are considered public goods that can therefore be shared by cooperators or exploited by cheaters under siderophore piracy.^119,120^ For instance, a strain of *V. cyclitrophicus* is able to shift its phenotype from siderophore producer to cheater in the presence of the exogenous siderophore desferrioxamine B.^121^ Under these conditions, it is energetically favorable for this bacterium to produce the corresponding exogeneous siderophore receptor rather than to increase their own siderophore production to compete for iron. Siderophore cheaters are common in *Vibrio*, where it has been noted that genes encoding for siderophore receptors surpass the number of biosynthetic gene clusters for these molecules, and in some cases these BGCs are not even present, making the bacteria an obligate cheater.^122^ Siderophores can also be used as a competitive strategy, locking iron and inducing iron starvation in competing bacteria. An example of this tactic has been found in *Vibrio fischeri*, a marine bacteria that secretes the siderophore aerobactin, limiting the growth of other *Vibrio* species.^123^ The ability to obtain iron from different siderophores would give *Microbulbifer* sp. CNSA002 an advantage, utilizing public goods and circumventing iron starvation. The genus Microbulbifer has been previously described as biopolymer-degrading marine bacteria, which produces hydrolytic enzymes for the breakdown of cellulose, xylan, chitin, and gelatin.^124^ We add to the biocatalytic capability of this genus by showing that some of the strains of these species can hydrolyze the backbone amide bond of peptidic hydroxamate siderophores.

### Annotation and Variable Detection of Additional Natural Products

**Figure 6:**
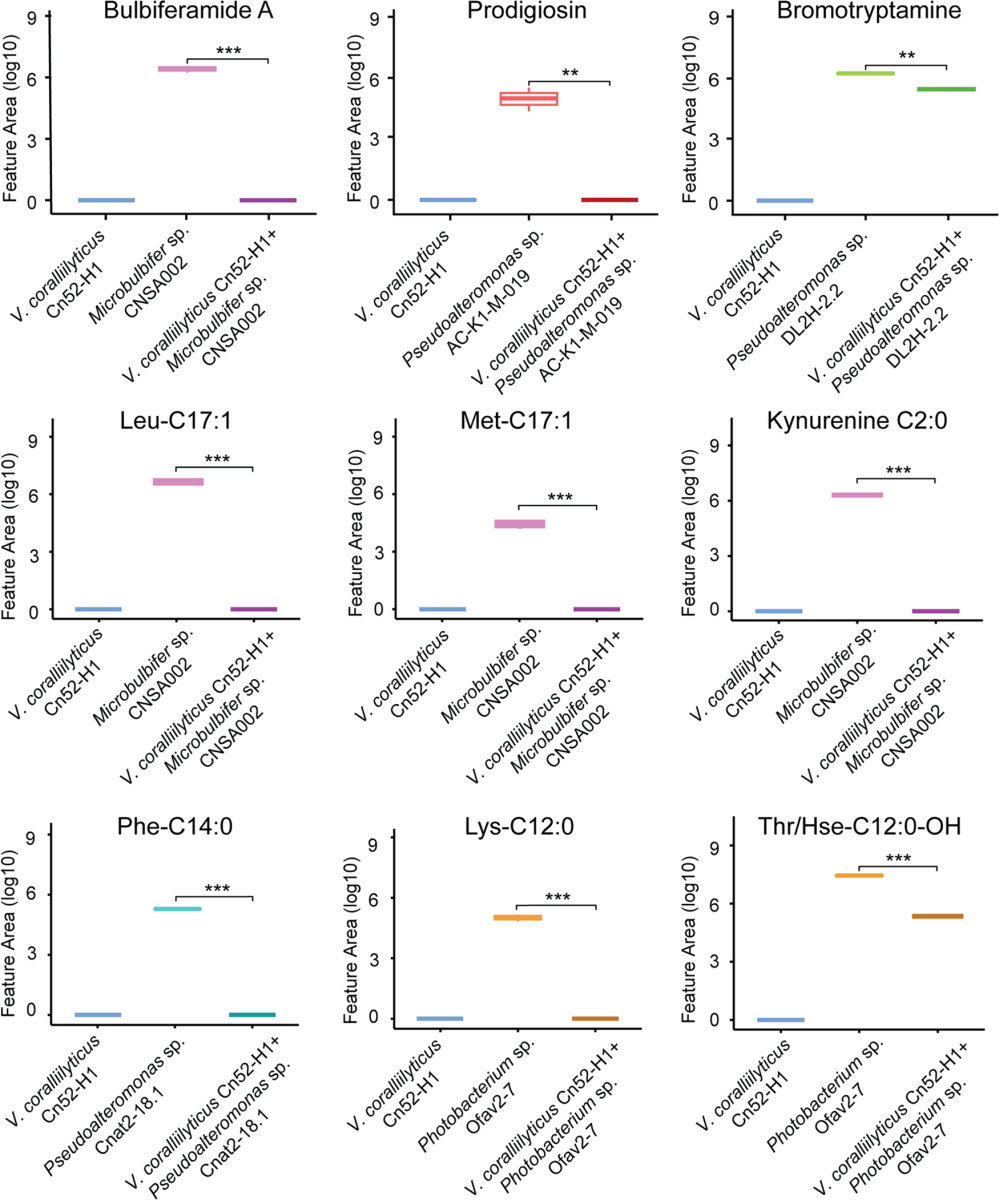
Boxplots of the relative abundances of bulbiferamide A, prodigiosin and bromotryptamine as well as a representative panel of *N*-acyl amides, in monoculture and coculture conditions. Asterisks indicate significant differences between the compared groups.

Using experimental and *in silico* spectral matching, several additional natural products were annotated in this data set. The production of some of these were found to be variable in mono- and coculture. Notably, several of these natural products, including clusters of bulbiferamides, prodigiosins, and bromotryptamines are either not detected in coculture with *V. coralliilyticus* Cn52-H1 or detected at significantly higher intensity in monoculture (visualized as pie chart representation in FBMN (Figures 2A and S1)) and as boxplots (Figure 6). The antimicrobial natural product prodigiosin^53^ was detected in the monocultures of the *Pseudoalteromonas* sp. strains AC-K1-M-019 and DL2H-2.2. However, no production was observed in coculture. A similar trend was observed for prodigiosin analogs annotated, including cycloprodigiosin and heptyl prodigiosin. This family of compounds exhibit a wide range of bioactivity including anticancer, antimicrobial, antialgal, and antiparasitic properties.^125−132^ Similarly, bulbiferamides were detected only in monoculture of *Microbulbifer* sp. CNSA002. These compounds are recently described ureidohexapeptides with antitrypanosomal activity.^60,62^ Additionally, several brominated tryptamines were detected in monoculture of *Pseudoalteromonas* sp. DL2H-2.2 and not in coculture. Brominated indoles have been previously isolated from marine sponges and bryozoans, exhibiting antioxidant activity and anti-inflammatory.^133−136^ Although there is no mention of a bacterial origin for these brominated compounds in sponges and bryozoans, it is common for marine natural products to be attributed to the animal host, even though they are often of bacterial origin.^137^ This observation is further supported by the identification of bioactive bromotryptamine analogs produced by the marine bacterium *Pseudoalteromonas rubra*.^138^ However, the possibility that these compounds are produced both by the host and animal cannot be eliminated. In a similar vein, we annotated multiple azulene analogs produced by *Pseudoalteromonas* sp. AC-K1-M-019 as well as *Pseudoalteromonas* sp. DL2H-2.2. These compounds are guaiane sesquiterpenes, which have been reported in different marine organisms such as corals and sponges.^139−141^ Compounds from this family have exhibited bioactivity against *Leishmania* and tumor cell lines, as immunomodulators, anti-inflammatory, antibacterial, antiviral and antiproliferative.^142−149^

Another class of compounds that was differentially detected between mono- and cocultures are *N*-acyl amides. We annotated a large diversity of this class of compounds (Figure S21 and Table S6) and observed differential detection of this class of compounds in monocultures of different strains as well as the in coculture of Cn52-H1 and CNSA002. Several of these compounds, produced by *V. coralliilyticus* Cn52-H1, were not detected in its coculture with *Microbulbifer* sp. CNSA002, as evidenced by the top50 feature heatmap (Figure S2 and Table S1). This class of features was detected across the board, with a large structural diversity for both the headgroup and acyl chain (Table S6). The highest number of annotated *N*-acyl amides was detected in *Pseudoalteromonas* sp. Cnat2–18.1 monocultures, including 15 *N*-acyl amides unique to this condition (Figure S22). *Photobacterium* sp. Ofav2–7 also stands out due to the number of unique members including citrulline headgroup containing *N*-acyl amides. *N*-acyl amides are ubiquitous in nature, as they are endogenous signaling molecules consisting of an amino acid covalently bound to a fatty acid.^150^ Despite their prevalence, their functions and biological mechanisms are largely understudied. Most of our knowledge about *N*-acyl amides (also known as lipoamino acids and elmiric acids) are described in human samples, where they are involved in inflammation, homeostasis and illnesses such as diabetes, cancer and neurodegenerative and cardiovascular diseases.^151−154^ In bacteria, *N*-acyl amides were shown to play a role in virulence,^155^ signaling,^156,157^ as antibiotics,^158−160^ surfactants,^161^ as a part of the membrane lipids^162,163^ and protein sorting systems.^164^ In our case, whether these molecules play a role as a chemical cue or antimicrobials remains to be explored.

The differential production of secondary metabolites by *Pseudoalteromonas* sp. and *Microbulbifer* sp. in coculture with *V. coralliilyticus* Cn52-H1 denotes natural product suppression upon coculture with *V. coralliilyticus* Cn52-H1. As observed in Figure 2B, 10% of features were uniquely detected in monoculture. Previously, subinhibitory concentrations of andrimid had been shown to elicit expression of silent biosynthetic gene clusters, particularly the induction of holomycin, in *Photobacterium galatheae* through a general stress response mechanism.^67^ However, when challenged with subinhibitory concentrations of another antibiotic, trimethoprim, natural product detection was attenuated corresponding to an SOS response.^67^ Therefore, the natural product suppression herein described could be attributed to multiple factors. As was observed for holomycin, different antibiotic elicitors can have varied effects on secondary metabolite production. Thus, exposure of these bacteria to andrimid or a different small molecule produced by *V. coralliilyticus* Cn52-H1 could result in inhibition of natural product biosynthesis, explaining why several natural products are only detected in monoculture and not in coculture. Depending upon the partner, cocultures can exhibit symbiotic interactions among its members such as parasitism, commensalism or mutualism.^165^ They can also display antagonistic relationships, through competition or predation.^165^ Due to antibiotic hormesis, increased exposure could lead to the observed inhibition.^166^ Natural product-mediated alteration of microbial species composition and interactions have been described in other environments also.^167^ Thus, cocultivation between different bacteria can aid in deciphering the interspecies interactions at play in the natural environment and allow for selection of phenotypes that may be beneficial to that environment.

## Conclusions

The coinfections by opportunistic pathogens during heat and other stressors is a major obstacle to the recovery of coral reefs akin to challenging recovery when infections of humans by SARS-COV-2 result in bacterial pneumonia. Furthermore, the opportunistic coral pathogens are often normal microflora of coral reefs, but their abundance increases during stressors resulting in exacerbation of disease. Thus, beneficial bacteria that can keep the pathogens at bay are a useful strategy to prevent coinfections. However, elucidating chemical interactions between these microbes in the native environment is challenging. In this work, we elucidated chemical interactions between coral-derived beneficial and pathogenic bacteria using discovery-centered untargeted metabolomics approach. Using this approach, we discovered a previously unknown tailoring of pathogen-synthesized peptide siderophores by the beneficial bacteria limiting their affinity for iron. Since iron is limited in the marine environment and the requirement for iron-dependent metabolic activity is large, siderophore production occurs widely in the marine environment. Thus, withholding iron from a pathogen is an important strategy to limit their proliferation. Furthermore, there is a fine balance between benefits and harmful effects of excess iron in coral reefs. On one side, iron is essential for the functioning of the microbiome, the endosymbiotic algae that reside inside coral cells, while excess iron can result in harmful phytoplankton blooms. Thus, maintaining healthy iron-levels through community interactions is important to the health of reefs. In the future, implications of this biotransformation in both the producer (*V. coralliilyticus*) and the coculture partner are important to study as a complex dynamic surrounding iron acquisition may be involved. For example, can this beneficial bacteria rescue overgrowth of *Vibrio* spp. bacteria in coral colonies under high heat conditions? Amphibactin is among the most-abundant siderophores detected in both the Atlantic and Pacific Ocean. Thus, our discovery of amphibactin degradation has important ecological implications. Further, biochemical experiments involving activity-based protein profiling will aid in identification of the specific protease in the protein fraction that is responsible for this activity. Lastly, our chemistry-first metabolomics approach has led us to the identification of this microbial interaction which would otherwise be missed by a genomics-first approach and can be potentially exploited to engineer microbiomes with higher resilience to iron starvation strategies from opportunistic pathogens.

## Methods

### Bacterial Isolation

*A. cervicornis* corals were sampled at the Georgia Aquarium and the fragments were stored in FASW at room temperature during. Mucus samples (2 mL) were collected from each fragment. Subsequently, the coral tissue was separated from the skeleton using an Iwata Siphon Feed Airbrush and collected in a sterile bag. The remaining skeleton was placed in a culture tube containing 5 mL of salt water broth (SWB^39^) and incubated at 30 °C for 48 h. The mucus, tissue slurry, and skeleton culture were diluted 5× and 10× before plating. A total of 36 samples were plated onto six different media: salt water agar (SWA), half-strength marine broth (Marine Broth 2216, BD Difco), Luria broth agar (LB, BD Difco), International Streptomyces Project-2 medium agar (ISP2, BD Difco), tryptic soy agar (TSA, BD Difco) and thiosulfate-citrate-bile salts-sucrose agar (TCBS, BD Difco). Plates were incubated at 30 °C for up to 10 days. Individual, morphologically distinct, colonies were subsequently streaked multiple times in the appropriate media for purification. Stock cultures of the 119 purified strains were prepared in 25% glycerol and frozen at −80 °C. Additional isolates were received from Dr. Valerie Paul at the Smithsonian Marine Station, isolated from corals *Montastraea cavernosa*, *C. natans*, *Pseudodiploria strigosa*, *O. faveolata* and *D. labyrinthiformis*.^23^ Several isolates were received from Dr. Vinayak Agarwal, Georgia Institute of Technology and were isolated from marine sponges *S. aurea*, *Aplysina fulva*, and *Aiolochroia crassa*.^66^

### Bacterial Coculturing

Morphologically diverse bacteria (Table 1) were prioritized for coculturing with *V. coralliilyticus* Cn52-H1 in triplicate, based on pigment production, taxonomic identification or their previous identification as potentially beneficial probiotic strains.^23^ Individual bacteria were also cultured in axenic culture in triplicates. Each strain was inoculated in 250 mL flasks containing 22 mL of SWB to an OD_600_ of 0.05. For cocultures, each isolate and *V. coralliilyticus* Cn52-H1 were inoculated together at an OD_600_ of 0.05 each. The cultures were incubated at 30 °C, shaking at 200 rpm for 24 h. Cultures were harvested and aliquoted into two 10 mL portions. The first portion was extracted using a liquid–liquid extraction (LLE) with ethyl acetate (EtOAc). Briefly, 10 mL of EtOAc was added to this portion, and then vortexed vigorously, and centrifuged at 2000*g* for 3 min. The extraction was repeated once more and the organic layers were removed with a glass pipet, pooled and dried *in vacuo* using a SpeedVac (Thermo Scientific, Waltham, MA). The second portion was centrifuged twice at 5250*g* for 15 min to separate the cell pellet from the supernatant. The supernatant was extracted via solid-phase extraction (SPE) using a 100 mg C_18_ column (Thermo Scientific, Waltham, MA), whereas the cell pellet was extracted using a solid–liquid extraction (SLE) with a 2:2:1 ethyl acetate:methanol:water (EtOAc:MeOH:H_2_O) solvent system. The SPE was performed by washing the column with 5 mL of 100% acetonitrile (MeCN), equilibrating with 5 mL of H_2_O and loading the supernatant to the column. Analytes bound to the column were eluted with 2.5 mL of 20%, 50% and 100% MeCN, eluates were pooled into a single extract and dried *in vacuo*. For the SLE, 6.7 mL of 2:2:1 EtOAc:MeOH:H_2_O were added to the cell pellet and vortexed vigorously every 30 min for 5 h. The extract was then centrifuged at 5250*g* for 15 min to remove the debris and dried *in vacuo*. Extracts were stored at −20 °C until data acquisition.

*Microbulbifer* sp. CNSA002 was also incubated with a cell-free supernatant of *V. coralliilyticus* Cn52-H1 and *vice versa*. Cell-free supernatants were prepared by freezing a 50 mL bacterial culture, thawing of the frozen culture, and centrifuging at 5250*g* for 15 min (performed twice). Supernatants were then filtered through a PES 0.2 μm membrane twice. For supernatant experiments, SWB was supplemented in a 1:1 ratio with each supernatant (22 mL total) and inoculated with the corresponding bacteria to an OD_600_ of 0.05. Monoculture controls were inoculated in 1:1 sterile SWB:FASW. Cultures were incubated at 30 °C, shaking at 200 rpm for 24 h and extracted using SPE as previously described. Supernatant controls were incubated and extracted in the same manner.

Other *Microbulbifer* sp. strains from our bacterial library were cocultured with *V. coralliilyticus* Cn52-H1, including VASA003, ZMAC003, VAAF005, MKSA007, and MLAF003. Incubation and metabolite extraction were performed as previously described.

### Untargeted Metabolomics Data Acquisition and Analysis

The dried extracts were resuspended in 300 μL of 100% MeOH containing 1 μM of sulfadimethoxine as an internal standard. Samples were vortexed, sonicated for 10 min and centrifuged at 16,160*g* for 15 min. The resuspended extracts were analyzed using an Agilent 1290 Infinity II Ultra High-Pressure Liquid Chromatography (UHPLC) system (Agilent Technologies; Santa Clara, CA) with a Kinetex 1.7 μm C_18_ reversed phase UHPLC column (50 × 2.1 mm^2^) (Phenomenex; Torrance, CA) coupled to an ImpactII ultrahigh resolution Qq-ToF mass spectrometer (Bruker Daltonics, GmbH, Bremen, Germany) equipped with an electrospray ionization (ESI) source. Chromatographic separation was performed with the following mobile phase gradient: 5% solvent B (MeCN, 0.1% (v/v) formic acid) and 95% solvent A (H_2_O, 0.1% (v/v) formic acid) for 3 min, a linear gradient of 5% B–95% B over 17 min, held at 95% B for 3 min, 95% B–5% B in 1 min, and held at 5% B for 1 min, 5% B- 95% B in 1 min, held at 95% B for 2 min, 95% B–5% B in 1 min, and held at 5% B for 2.5 min, at a flow rate of 0.5 mL/min throughout. MS spectra were acquired in positive ionization mode from *m*/*z* 50 to 2000 Da. An external calibration with ESI-L Low Concentration Tuning Mix (Agilent Technologies) was performed prior to data collection, and hexakis(1*H*,1*H*,2*H*-perfluoroethoxy)phosphazene was used as an internal lock-mass calibrant throughout the run. For MS^2^ data acquisition, the eight most intense ions per MS^1^ were selected for fragmentation. A basic stepping function was used to fragment ions at 50 and 125% of the CID calculated for each *m*/*z* with timing of 50% for each step. The MS/MS active exclusion parameter was set to two, and the active exclusion was released after 30s. The mass of the internal lock-mass calibrant was excluded from the MS^2^ list. UV data was acquired with a UV DAD detector (Agilent Technologies) from 190 to 400 nm, with a 2 nm step. Zero offset was set at 5% along a 1000 mAU attenuation. Data was acquired throughout the LC run with a >0.1 min peak width.

Raw data were converted to mzXML format, using vendor proprietary software. Metabolite features were extracted using MZmine 2.53,^68^ performing mass detection, chromatogram building, chromatogram deconvolution, isotopic peak grouping, retention time alignment, replicate filtering, duplicate peak removal, and gap filling. The resulting processed data was submitted to the Global Natural Product Social Molecular Network platform (GNPS) to generate a feature-based molecular network (FBMN). The molecular network was generated using the following parameters: fragment ions were removed within a ±17 Da window of the precursor *m*/*z*, precursor ion and fragment ion mass tolerance were set to 0.02 Da and edges were filtered to have a score above 0.7 and at least 4 matched peaks. Edges were kept if both nodes were present in each other’s top 10 most similar nodes, and molecular families’ maximum size was set to 100. Experimental fragmentation spectra were searched against GNPS’s spectral libraries and filtered in the same way (cosine score above 0.7 and a minimum of 4 matched peaks). The workflows for DEREPLICATOR,^168^ DEREPLICATOR+,^169^ MolDiscovery^170^ and MS2LDA^74^ were also run on GNPS. The output from MZmine was additionally exported for analysis with SIRIUS^72^ 5.3.6. with CSI:FingerID^171^ and CANOPUS.^73^ These tools provide putative annotations, which are confirmed using an MS^2^ spectral comparison with literature reported spectra, in-house spectra, with data acquired on analytical standards, and manual annotation of MS^2^ fragments resulting in level 2 compound annotations. SIRIUS computes putative chemical formulas using MS^1^ and fragmentation trees (based on user uploaded MS^1^ isotopic peaks and MS^2^ fragmentation patterns). CSI: FingerID transforms MS^2^ spectra into predicted structural fingerprints that enable matching to chemical databases. CANOPUS predicts the chemical class of metabolites by utilizing CSI:FingerID’s predicted structural fingerprints. Library searches were performed for The Natural Product Atlas^75^ and MarinLit.^76^ The molecular network was visualized using Cytoscape^70^ v3.9.0 and features present in the blanks were subtracted. UpSet plots were generated using the Intervene^71^ platform, to facilitate data visualization and statistical analysis were performed on the Metaboanalyst platform.^172^

### Fermentation, Extraction, and Compound Purification

*Microbulbifer* sp. CNSA002 and *V. coralliilyticus* Cn52-H1 were inoculated at an OD_600_ of 0.05 in 1 L flasks containing 200 mL of SWB. Cultures were incubated at 30 °C, shaking at 200 rpm for 24 h. Harvested cultures (5 L) were centrifuged at 4920*g* for 25 min at 4 °C to separate the cell pellet from supernatant. The cell-free supernatant was mixed with 4% (*w/v*) XAD-16 resin (Sigma-Aldrich, St. Louis, MO) and stirred for 16 h. Afterward, the supernatant was removed, and the resin was washed with 500 mL of H_2_O and eluted with 200 mL of ethanol (EtOH). The solvent was dried *in vacuo* to yield a 1.7 g crude extract.

The crude extract was resuspended in H_2_O and fractionated via reverse phase liquid chromatography (RPLC) using a 10 g C_18_ column (Thermo Scientific, Waltham, MA). The column was washed with 150 mL of MeCN and equilibrated with 150 mL of H_2_O. The crude was loaded, and the analytes were eluted with 10, 20, 40 60 and 100% MeCN to obtain five fractions (1–5) which were dried *in vacuo*. Fraction 3 (40% MeCN) was further fractionated using an Agilent 1260 Infinity II Liquid Chromatography (semipreparative HPLC) system (Agilent Technologies) equipped with a Luna 5 μm C_18_ reversed phase HPLC column (250 × 10 mm^2^) (Phenomenex). The mobile phases used for chromatographic separation were solvent B: MeCN, 0.1% (v/v) trifluoroacetic acid (TFA) and solvent A: H_2_O, 0.1% (v/v) TFA. The gradient was performed with the following mobile phase compositions: a linear gradient from 10% solvent B to 100% solvent B in 25 min, held at 100% B for 10 min, at a flow rate of 2 mL/min throughout. Elution was monitored with a UV detector monitoring the run at 205 and 310 nm. This fractionation yielded compound **1** (3.0 mg).

Compound **1**: yellow, oily. ^1^H and ^13^C NMR data, see Table S4. Positive HRESIMS *m*/*z* 617.4121 [M + H]^+^ (calculated for C_30_H_57_N_4_O_9_, 617.4120)

One-dimensional (1D) and two-dimensional (2D) NMR spectra were recorded on a Bruker Avance III HD 700 MHz NMR, in DMSO-*d*_6_ and calibrated using residual undeuterated solvent as internal reference. The chemical shift (δ) is reported in parts per million (ppm) and the coupling constants (*J* values) are in Hz. NMR data for compound **1** has been deposited to NP-MRD^173^ (ID: NP0341873).

### O-CAS Agar Assay

The iron chelating activity of Compound **1** was confirmed via a modified CAS assay (O-CAS^80^). A 1 μL aliquot of a 1 mg/mL methanolic solution of compound **1** was spotted on an O-CAS agar plate.^80^ Desferrioxamine mesylate (Sigma-Aldrich) 1 mg/mL was used as a positive control, whereas methanol was spotted as a negative control. The plate was incubated at room temperature for 30 min before registering the results. An O-CAS agar plate was also overlaid on a colony of *Microbulbifer* sp. CNSA002 grown on SWA.

### CNSA002 Whole Genome Sequencing

The cell pellet from 2 mL of *Microbulbifer* sp. CNSA002 culture was collected by centrifugation at 16000*g* for 2 min. The cell pellet was solubilized in a 120 μL aliquot of 10 mg/mL lysozyme in 10 mM Tris–HCl, pH 8.0 buffer and incubated at 37 °C for 45 min. Cells were collected by centrifugation and gDNA was isolated following the Wizard Genomic DNA purification kit (Promega) specifications. The size of gDNA was checked on a 0.5% agarose gel at 120 V for 40 min. The quality and concentration of gDNA was measured on a NanoDrop spectrophotometer (Thermo Scientific). Samples were sent for sequencing to SeqCenter (Pittsburgh), and Illumina and Nanopore sequencing were performed. The genome sequence has been deposited to NCBI under the bioproject PRJNA1173768.

### Enzymatic Hydrolysis of Amphibactin

To test the enzymatic nature of the amphibactin degradation, a 20 mL aliquot of SWB was inoculated with *Microbulbifer* sp. CNSA002 and incubated at 30 °C for 24 h, shaking at 200 rpm. The cell pellet and culture supernatant were separated by centrifugation at 4920*g* for 45 min at 4 °C. A 10 mL aliquot of phosphate buffer (50 mM Na_3_PO_4_, pH 7.0) was added to the cell pellet and subsequently sonicated on ice for 30 min (40% amplitude, 20 s on, 40 s off) to obtain the cell lysate. The culture supernatant and cell lysate were tested for protease activity. A portion of each fraction was boiled at 95 °C for 10 min to denature the proteins and tested for protease activity. The culture supernatant was additionally fractionated using two molecular weight cutoff (MWCO) Amicon ultracentrifugation filters (3 kDa and 10 kDa, Sigma-Aldrich) and the four resulting fractions were tested for activity.

In order to assay the activity of each fraction, the amphibactin-containing *V. coralliilyticus* Cn52-H1 cell-free supernatant was employed. A 900 μL aliquot of *V. coralliilyticus* Cn52-H1 cell-free supernatant was supplemented with 100 μL of each fraction. Reactions were incubated at 30 °C, shaking at 200 rpm overnight and extracted via SPE. The SPE was performed using a 25 mg C_18_ column (Thermo Scientific, Waltham, MA). The columns were washed with 1 mL of 100% MeCN and equilibrated with 1 mL of H_2_O. The reactions were loaded into the column and analytes bound to the column were eluted with 1 mL of 20, 50 and 100% MeCN. Eluates were pooled into a single extract, dried *in vacuo* and stored at −20 °C until data acquisition. The dried extracts were resuspended in 60 μL of 100% MeOH containing 1 μM of sulfadimethoxine as an internal standard. Samples were prepared and LCMS data was acquired as previously detailed.

### Ferrichrome and Desferrioxamine Degradation Assays

To determine if *Microbulbifer* sp. CNSA002 can degrade the siderophore ferrichrome (Sigma-Aldrich), a 15 mL aliquot of SWB was supplemented with either desferrichrome or ferrichrome (6.67 μM) and inoculated with *Microbulbifer* sp. CNSA002. Controls for both the apo- and holosiderophore, as well as for the bacterium and media control were set up. To test for desferrioxamine degradation, cocultures of *Microbulbifer* sp. CNSA002 and the desferrioxamine-producing *Pseudoalteromonas* sp. Cnat2–18.1 were inoculated. A *Microbulbifer* sp. CNSA002 was cultured in SWB supplemented with desferrioxamine B (0.6 mM) (Sigma-Aldrich). Monocultures of each bacteria and for the media were set up. Cultures were incubated at 30 °C for 24 h, shaking at 200 rpm. Cultures were harvested and extracted via SPE, dried down and reconstituted for LC-MS/MS analysis as previously described.

## Data Availability

*Mirobulbifer* sp. CNSA002 whole genome sequence has been deposited in NCBI under the bioproject PRJNA1173768. NMR data for compound 1 has been deposited to NP-MRD^173^ (ID: NP0341873). LCMS data has been deposited in GNPS-MassIVE and the data set ID is MSV000096127.

## Supporting Information Available

Representative feature-based molecular networking for mono- and coculture experiments (Figure S1); Heatmap of the top 50 features in monocultures and coculture of *V. coralliilyticus* Cn52-H1 and *Microbulbifer* sp. CNSA002 (Figure S2); natural product extraction and characterization workflow (Figure S3); amphibactin F degradation product, elucidated through 1D and 2D NMR experiments (Figure S4); NMR data for compound 1 (Figures S5–S12); MS^2^ spectral analysis of amphibactins and new amphibactin analogs (Figures S13 and S14); *Microbulbifer* sp. CNSA002 O-CAS agar assay (Figure S15); detection of degraded amphibactins in *V. coralliilyticus* Cn52-H1 in coculture with *Microbulbifer* sp. CNSA002 and supplemented with *Microbulbifer* sp. CNSA002 supernatant and CNSA002 fractions (Figures S16 and17); Boxplot of the relative abundances of desferrioxamine G and ferrioxamine G in *Pseudoalteromonas* sp. Cnat2–18.1 and *Microbulbifer* sp. CNSA002 monocultures and coculture (Figure S18); mirror plots comparing experimental MS^2^ spectra of natural products, hydroxamate siderophores and *N*-acyl amides detected in this study with spectra deposited in the GNPS library (Figures S19–21); upset plot for *N*-acyl amides detected in different monocultures and cocultures (Figure S22); putative annotation of top 50 features present in the *V. coralliilyticus* Cn52-H1 and *Microbulbifer* sp. CNSA002 coculture heatmap (Table S1); putative annotation of 76 features uniquely detected in marine bacteria cocultures with *V. coralliilyticus* Cn52-H1 (Table S2); list of annotated metabolites detected in this study (Table S3); ^1^H (700 MHz) and ^13^C (176 MHz) NMR data of compound 1 in DMSO-*d*_6_ (Table S4); biosynthetic gene clusters present in *Microbulbifer* sp. bacteria analyzed in this study (Table S5); list of annotated *N*-acyl amides detected in this study (Table S6) (PDF)
