# Supplementary material for: Discovery of Peptidic Siderophore Degradation by Screening Natural Product Profiles in Marine-Derived Bacterial Mono- and Cocultures
Source: Biochemistry. 2025 Jan 14;64(3):634–54. doi: 10.1021/acs.biochem.4c00706 (PMC11800396; doi:10.1021/acs.biochem.4c00706)
Supplement: Supplementary file 1 — bi4c00706_si_001.pdf [file bi4c00706_si_001.pdf]

Supporting information

Discovery of peptidic siderophore degradation by screening natural product profiles in marine-derived bacterial mono- and cocultures.

Mónica Monge-Loría[1], Weimao Zhong[1], Nadine H. Abrahamse[1], Stephen Hartter[2],

Neha Garg*[1,3]

[1]School of Chemistry and Biochemistry, Georgia Institute of Technology. 950 Atlantic Drive, Atlanta, GA 30332 (USA).

[2]Georgia Aquarium. 225 Baker St. NW, Atlanta, GA 30313 (USA).

[3]Center for Microbial Dynamics and Infection, Georgia Institute of Technology. 315 Ferst Drive, Atlanta, GA 30332 (USA).

Correspondence: neha.garg@chemistry.gatech.edu

Table of Contents

Figure S1. Representative feature-based molecular networking for mono- and coculture experiments. 3
Figure S2. Heatmap of the top 50 features in monocultures and coculture of *V. coralliilyticus* Cn52-H1 and *Microbulbifer* sp. CNSA002 4
Figure S3. Natural product extraction and characterization workflow. 5
Figure S4. Amphibactin F degradation product, elucidated through 1D and 2D NMR experiments. 5
Figure S5. The ^{1}H NMR spectrum of compound **1** (700 MHz, DMSO- d_6) 6
Figure S6. The ^{13}C NMR spectrum of compound **1** (176 MHz, DMSO- d_6) 7
Figure S7. The DEPT135 NMR spectrum of compound **1** (176 MHz, DMSO- d_6) 8
Figure S8. The HSQC spectrum of compound **1** (700 MHz, DMSO- d_6) 9
Figure S9. The HMBC spectrum of compound **1** (700 MHz, DMSO- d_6) 10
Figure S10. The ^{1}H-^{1}H COSY NMR spectrum of compound **1** (700 MHz, DMSO- d_6) 11
Figure S11. The ^{1}H-^{1}H TOCSY spectrum of compound **1** (700 MHz, DMSO- d_6) 12
Figure S12. The ROESY spectrum of compound **1** (700 MHz, DMSO- d_6) 13
Figure S13. MS^2 spectral analysis of amphibactins and degradation products. 16
Figure S14. MS^2 spectral analysis of new amphibactin analogs, compared to amphibactin B. . 17
Figure S15. *Microbulbifer* sp. CNSA002 O-CAS agar assay 18
Figure S16. Detection of degraded amphibactins in *V. coralliilyticus* Cn52-H1 in coculture with *Microbulbifer* sp. CNSA002 and supplemented with *Microbulbifer* sp. CNSA002 supernatant... 19
Figure S17. Detection of degraded amphibactins in *V. coralliilyticus* Cn52-H1 supernatant, supplemented with different *Microbulbifer* sp. CNSA002 fractions 20

Figure S18. Boxplot of the relative abundances of desferrioxamine G and ferrioxamine G (619.366 *m/z* and 672.277 *m/z* respectively) in *Pseudoalteromonas* sp. Cnat2-18.1 and *Microbulbifer* sp. CNSA002 monocultures and coculture...21
Figure S19. Mirror plots comparing experimental MS^2 spectra of natural products detected in this study with spectra deposited in the GNPS library. 22
Figure S20. Mirror plots comparing experimental MS^2 spectra of hydroxamate siderophores and HPTzTn-COOH detected in this study with spectra deposited in the GNPS library. 23
Figure S21. Mirror plots comparing experimental MS^2 spectra of *N*-acyl amides detected in this study with spectra deposited in the GNPS library...24
Figure S22. Upset plot for *N*-acyl amides detected in different monocultures and cocultures. The top 8 intersections are shown for clarity. 25

Table S1. Putative annotation of top 50 features present in the *V. coralliilyticus* Cn52-H1 and *Microbulbifer* spp. CNSA002 coculture heatmap. 26
Table S2. Putative annotation of 76 features uniquely detected in marine bacteria cocultures with *V. coralliilyticus* Cn52-H1. 28
Table S3. List of annotated metabolites detected in this study. 33
Table S4. ^{1}H (700 MHz) and ^{13}C (176 MHz) NMR data of compound **1** in DMSO- d_6 (*J* in Hz, *δ* in ppm)..35
Table S5. Biosynthetic gene clusters present in *Microbulbifer* sp. bacteria analyzed in this study. 36
Table S6. List of annotated *N*-acyl amides detected in this study..37

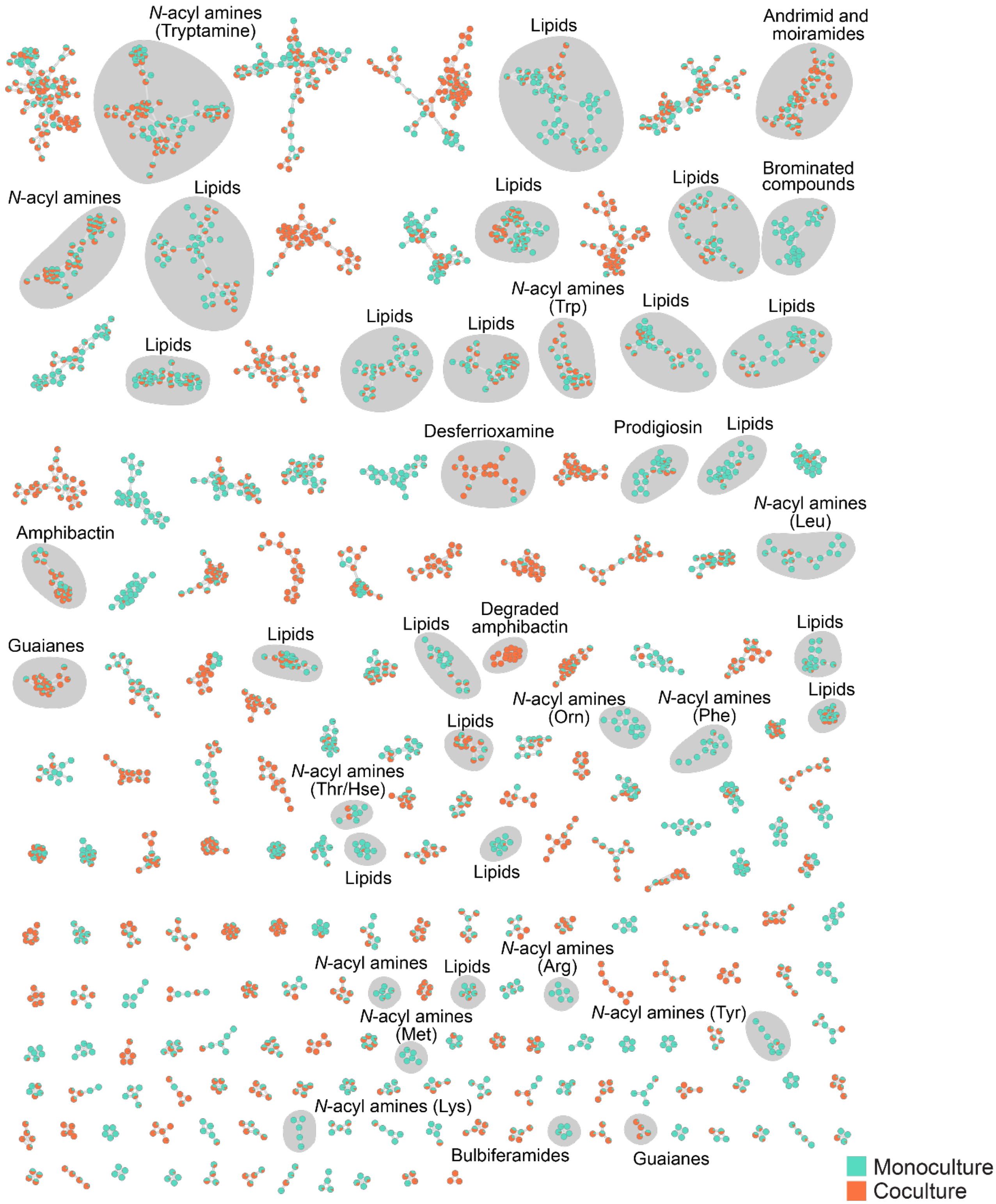

Figure S1. Representative feature-based molecular networking for mono- and coculture experiments. Annotated clusters and identified compound classes are shaded in gray. Singletons are removed for clarity.

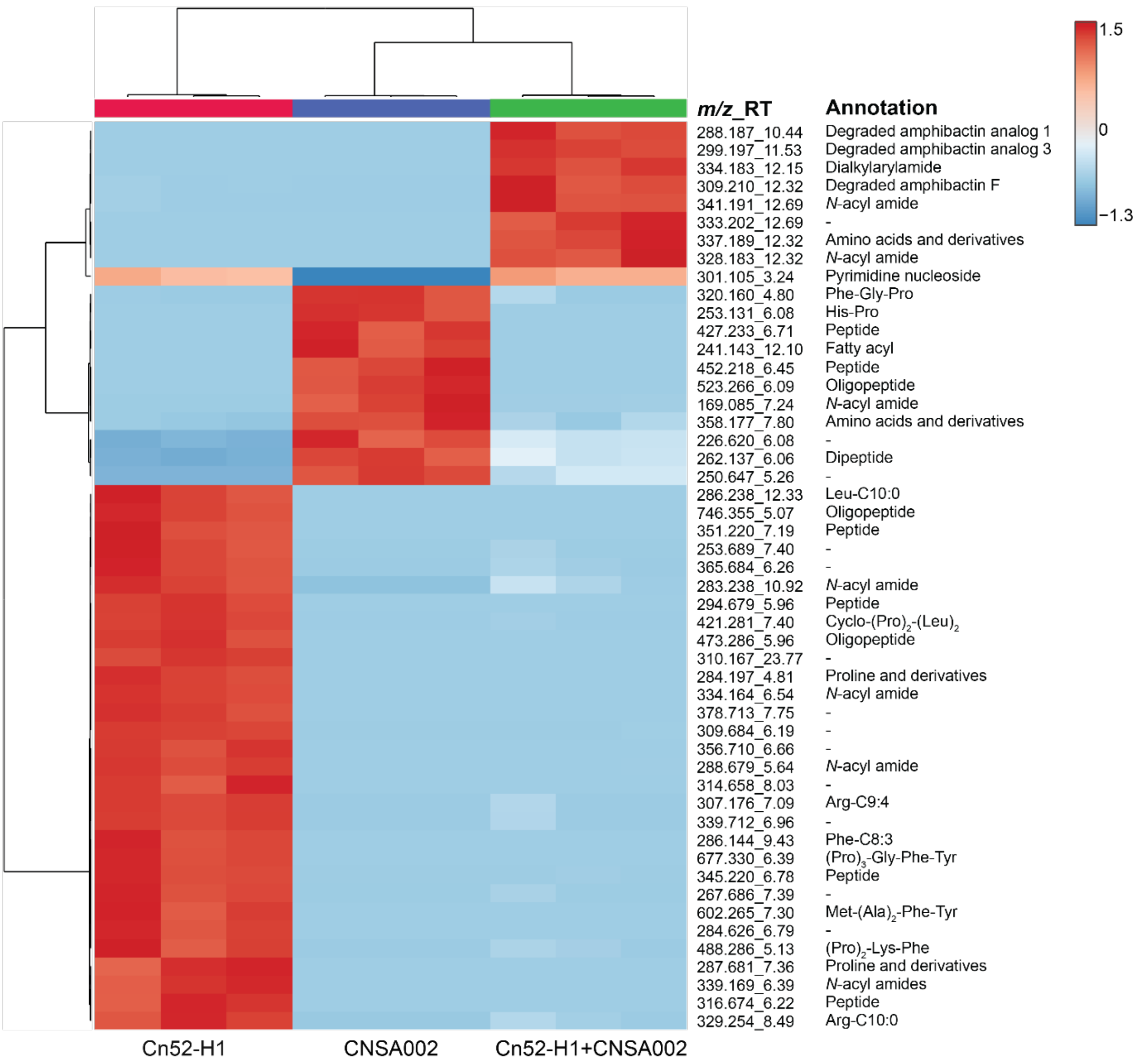

Figure S2. Heatmap of the top 50 features in monocultures and coculture of *V. coralliilyticus* Cn52-H1 and *Microbulbifer* sp. CNSA002 showing the differences in their relative abundances

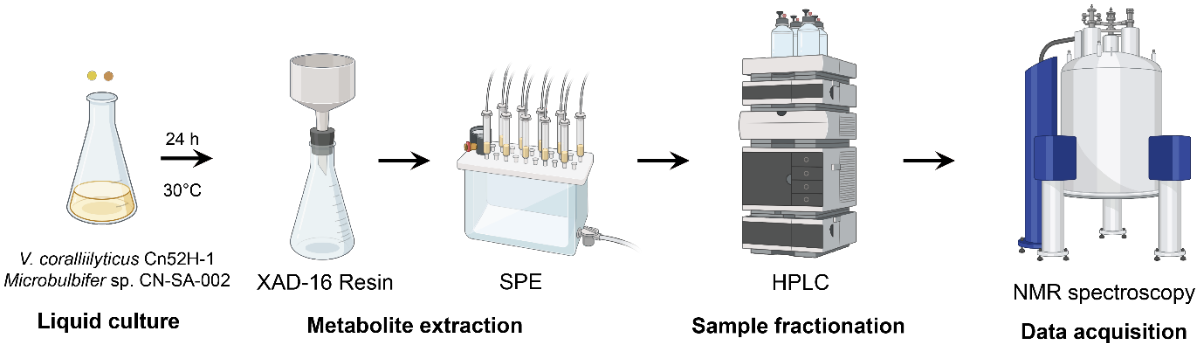

Figure S3. Natural product extraction and characterization workflow. In order to elucidate the structure of the compounds of interest, a large-scale *V. coralliilyticus* Cn52-H1 and *Microbulbifer* sp. CNSA002 coculture was extracted and fractionated. NMR data was acquired on fractions of interest for structure elucidation

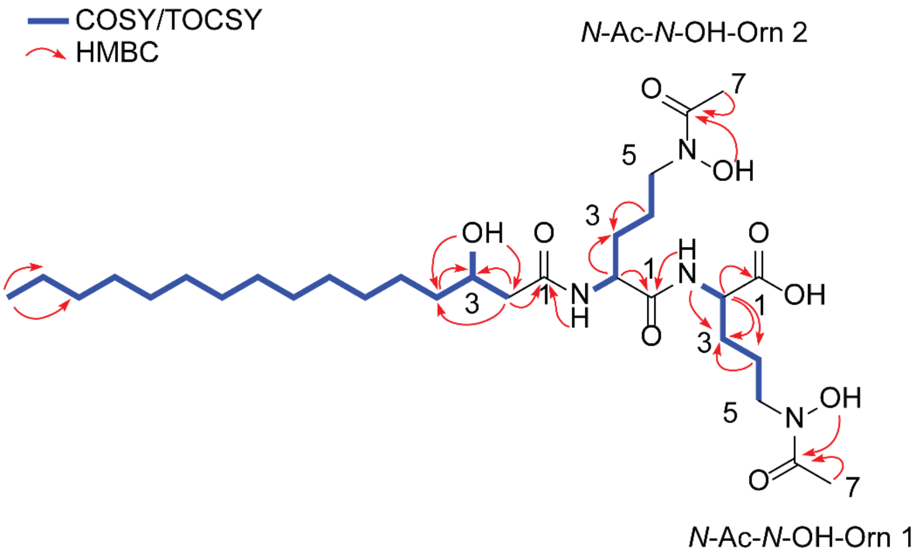

Figure S4. The structure of degraded Amphibactin F, elucidated through 1D and 2D NMR experiments.

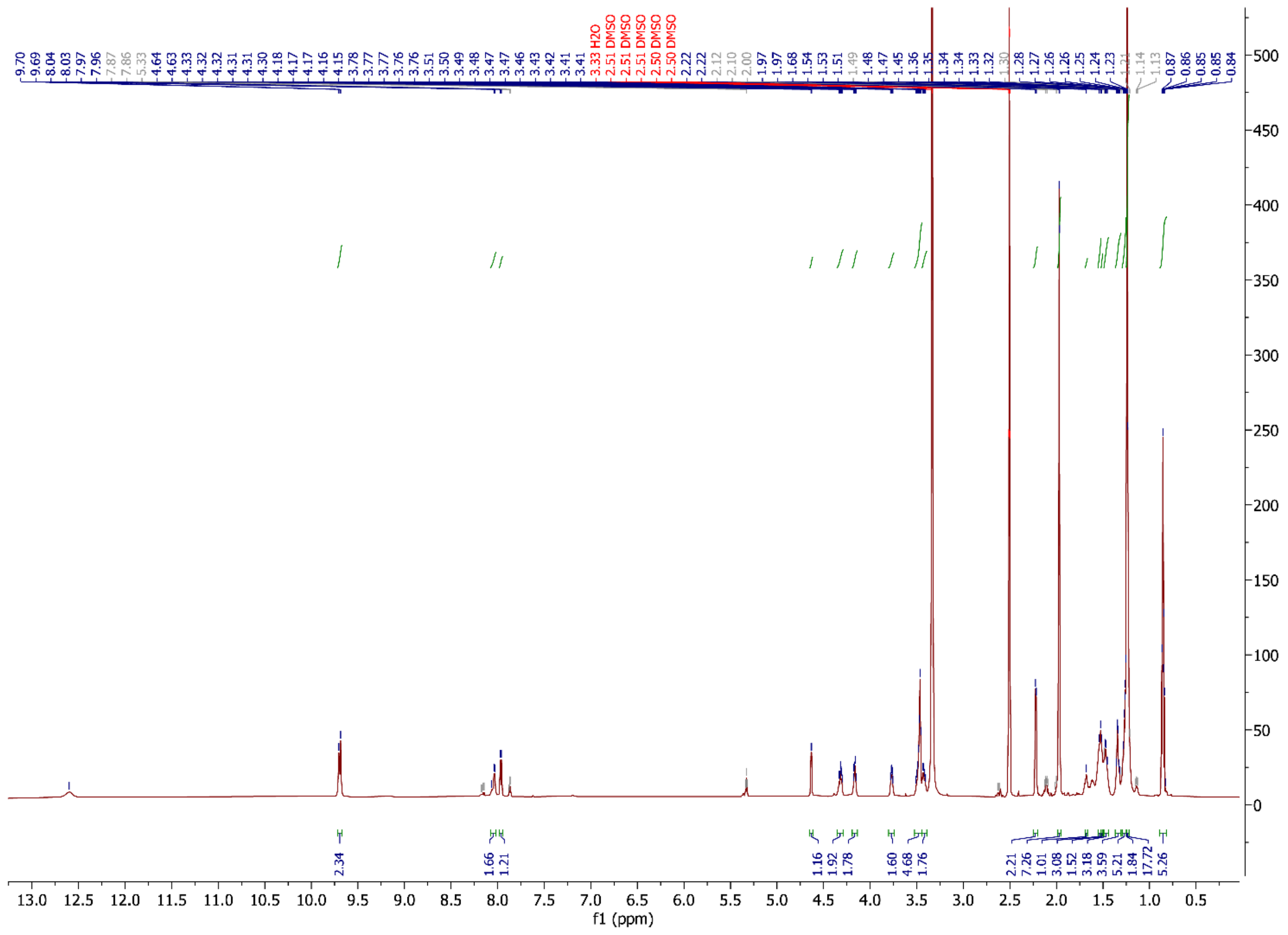

Figure S5. The ^1H NMR spectrum of compound **1** (700 MHz, DMSO- d_6).

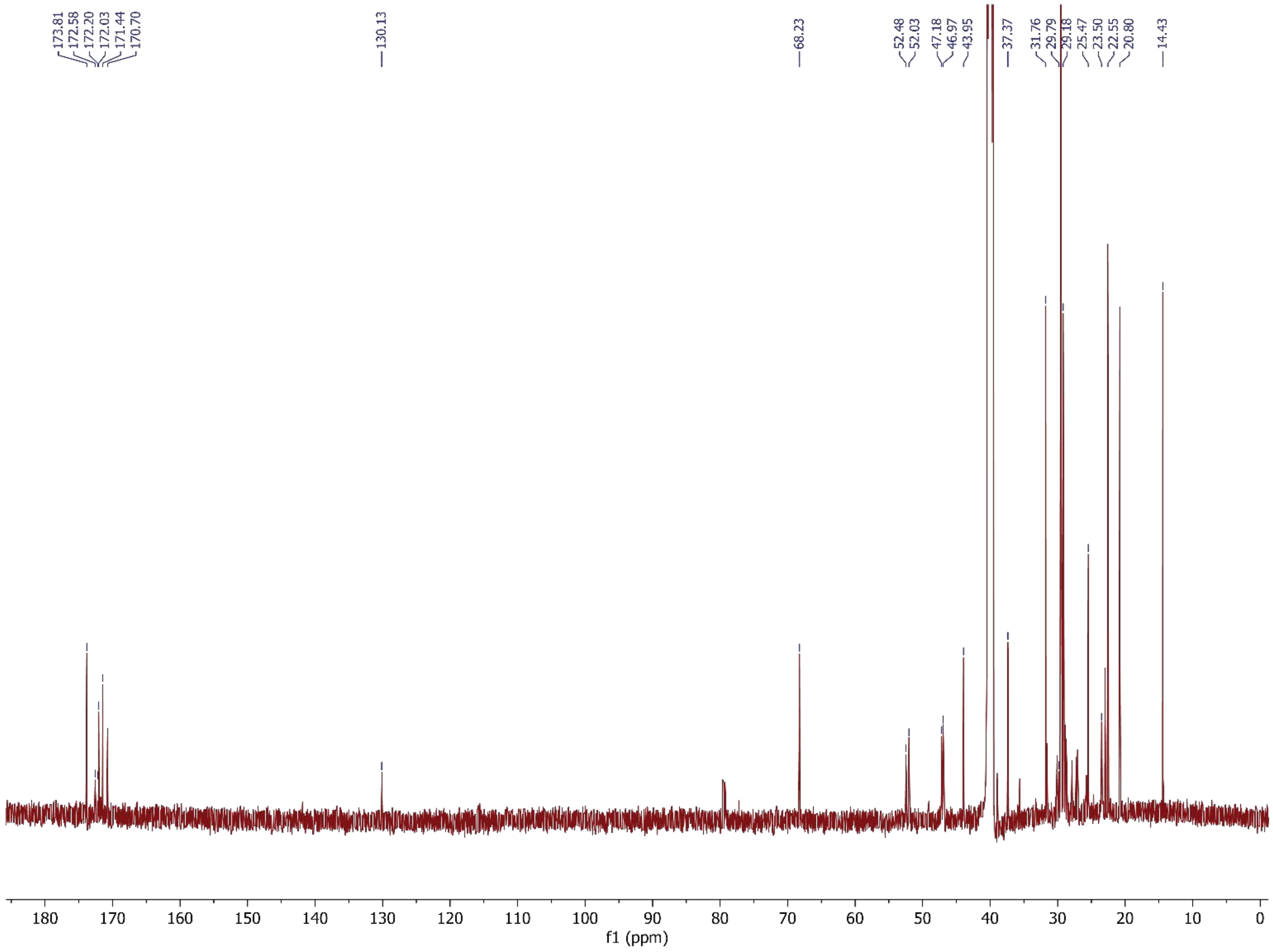

Figure S6. The ^{13}C NMR spectrum of compound **1** (176 MHz, DMSO- d_6).

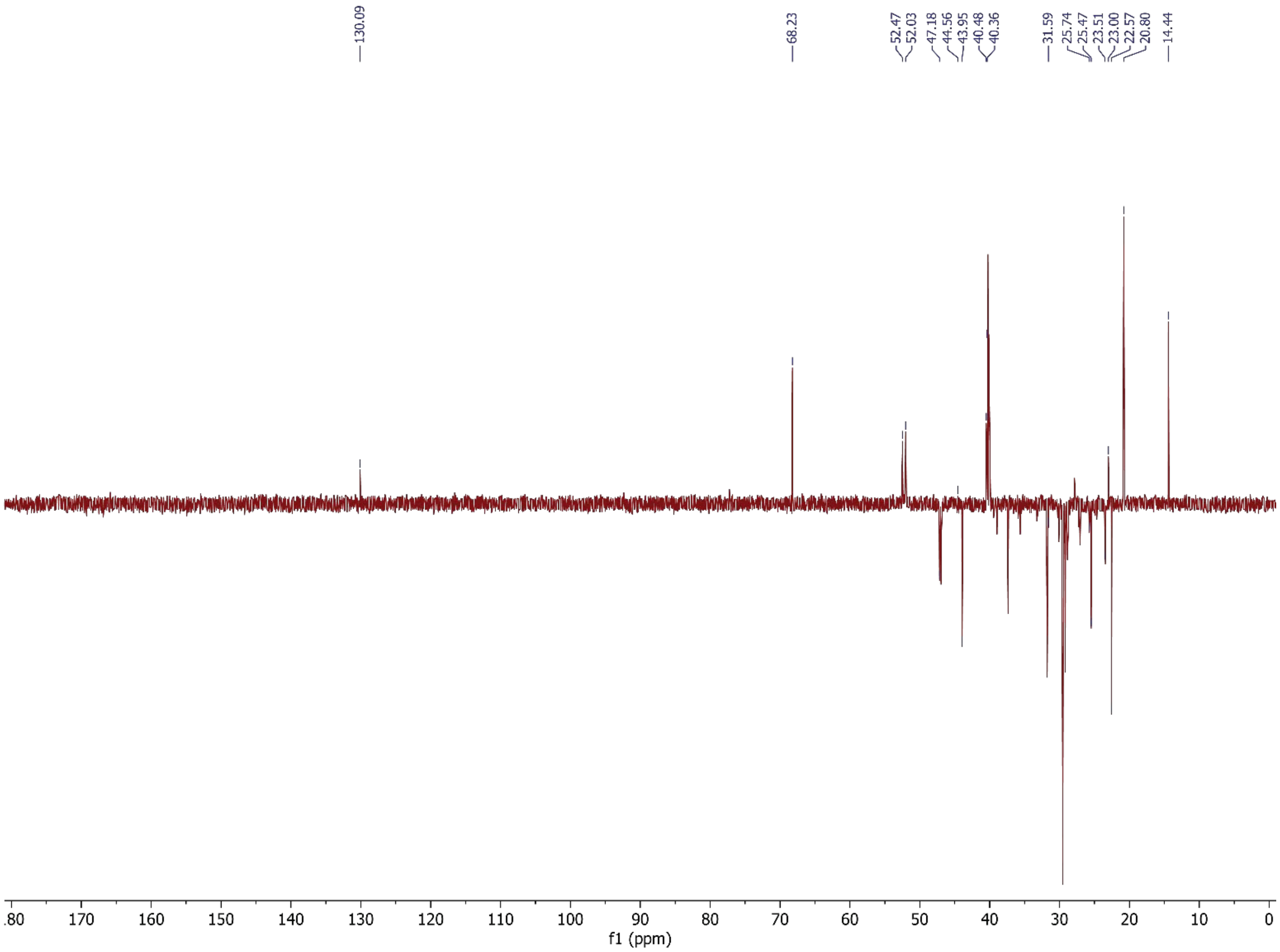

Figure S7. The DEPT135 NMR spectrum of compound **1** (176 MHz, DMSO- d_6).

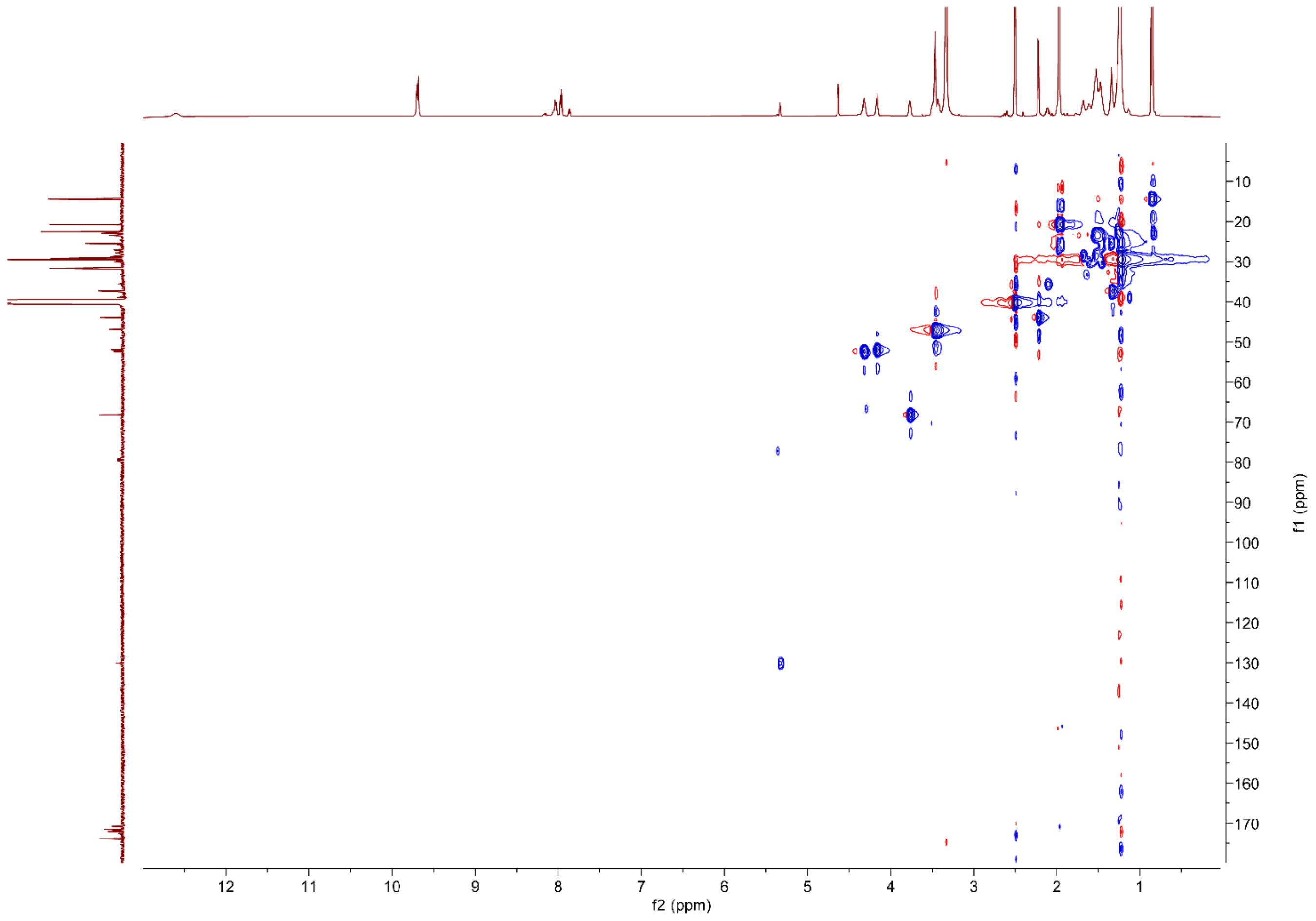

Figure S8. The HSQC spectrum of compound **1** (700 MHz, DMSO- d_6).

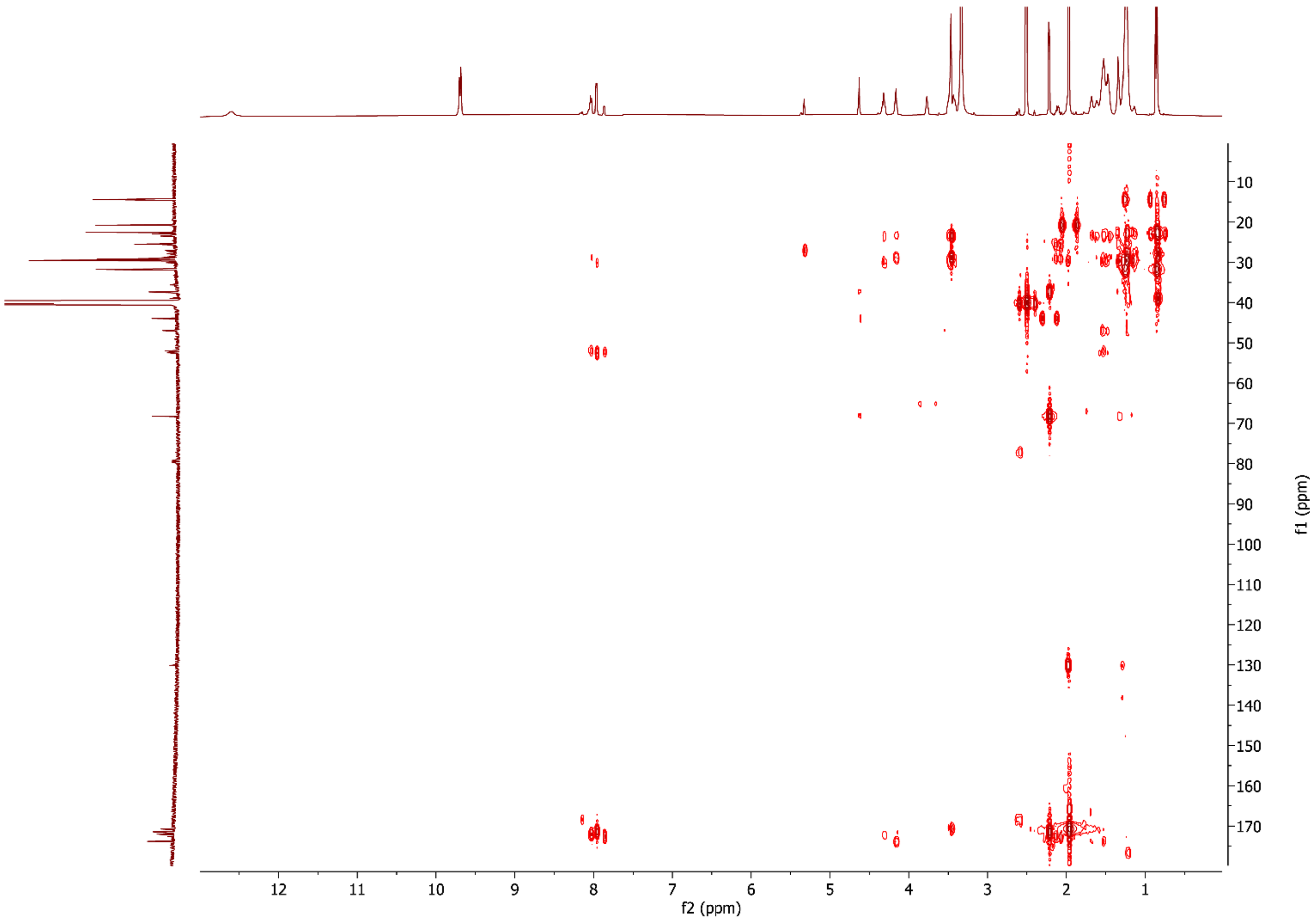

Figure S9. The HMBC spectrum of compound **1** (700 MHz, DMSO- d_6).

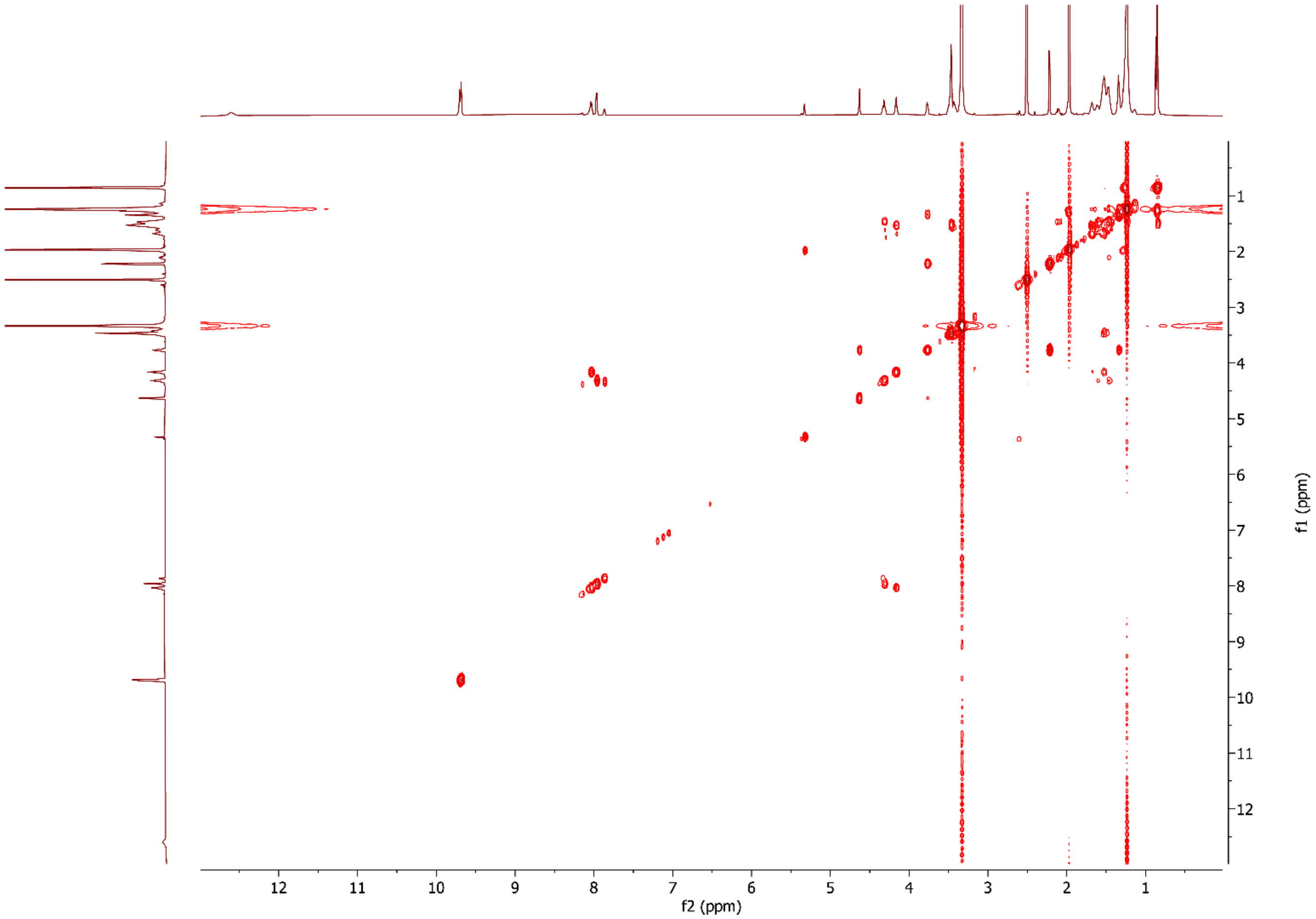

Figure S10. The ^{1}H-^{1}H COSY NMR spectrum of compound **1** (700 MHz, DMSO- d_6).

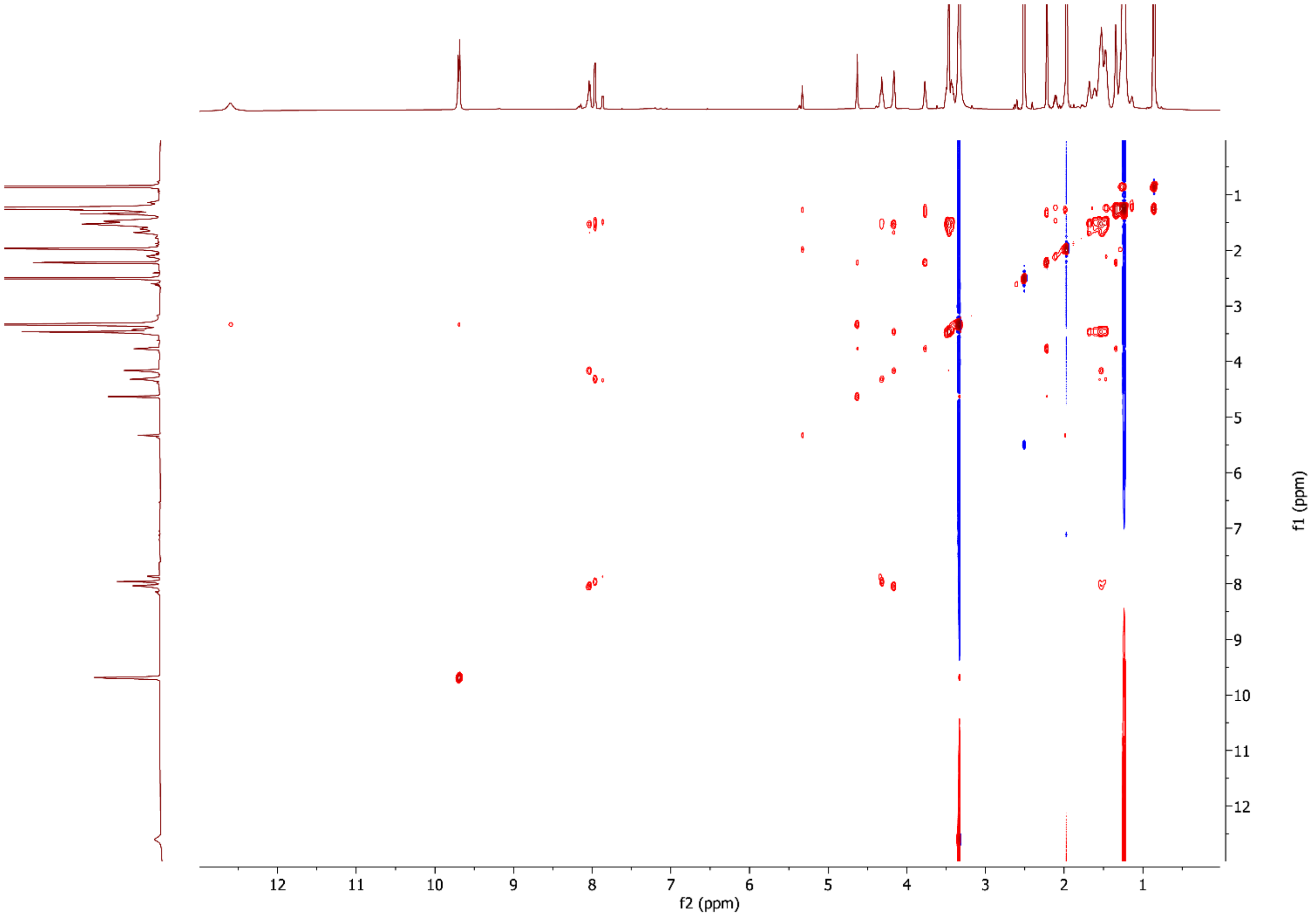

Figure S11. The ^{1}H-^{1}H TOCSY spectrum of compound **1** (700 MHz, DMSO- d_6).

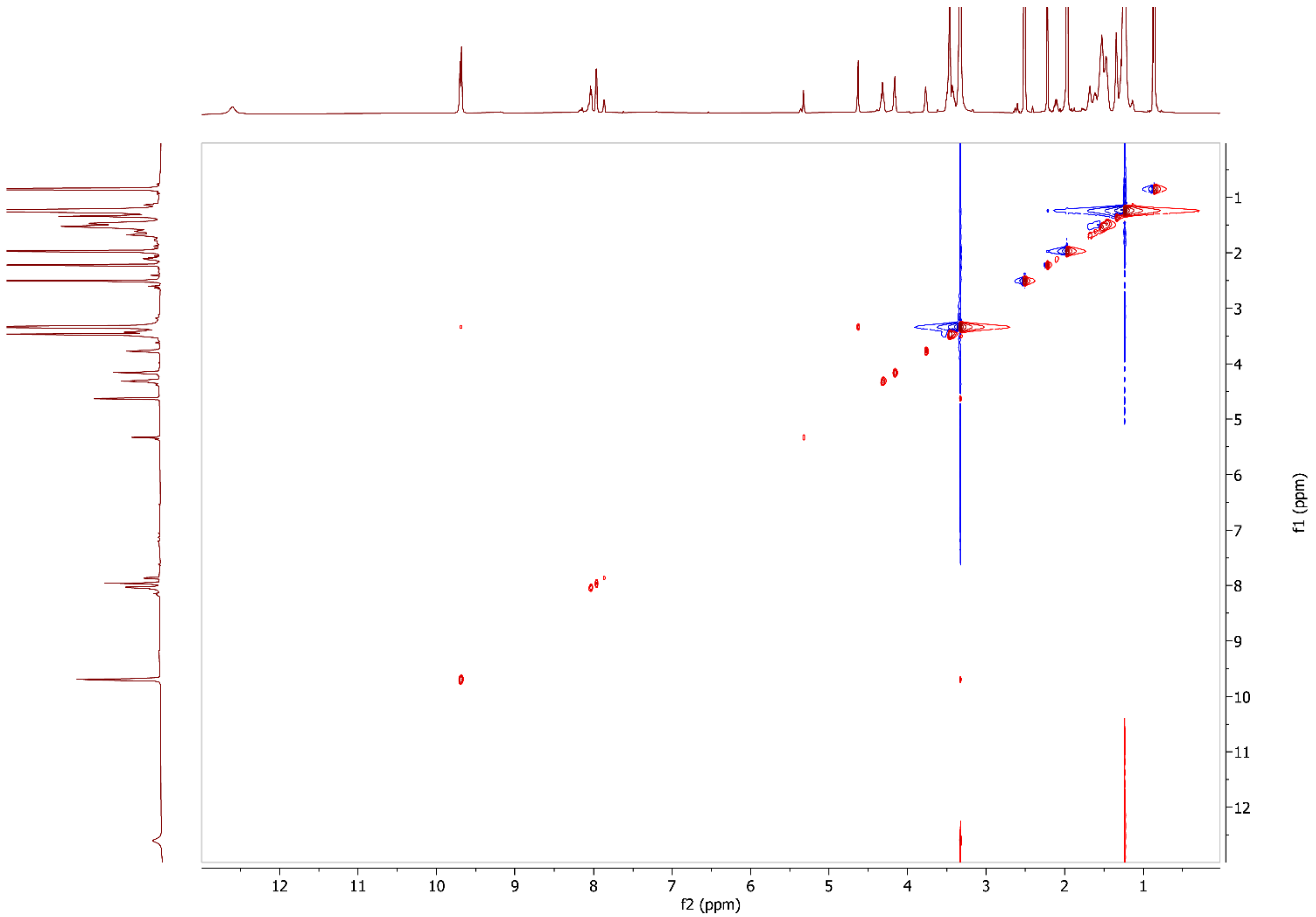

Figure S12. The ROESY spectrum of compound **1** (700 MHz, DMSO- d_6).

Amphibactin F

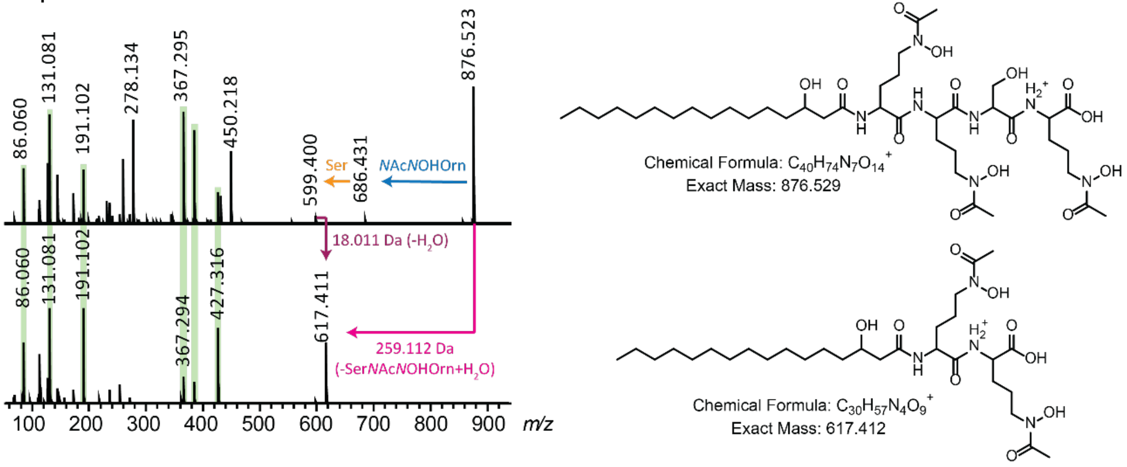

Amphibactin B

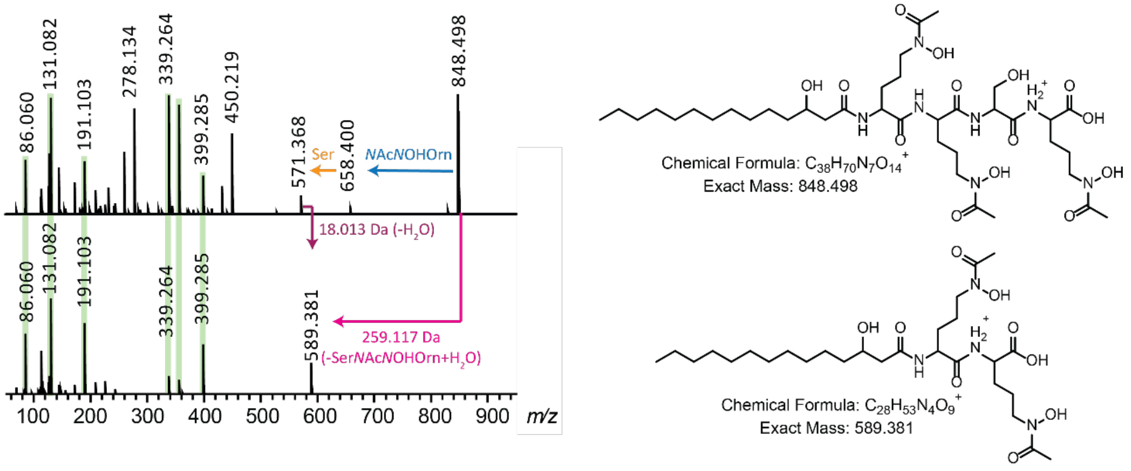

Amphibactin C

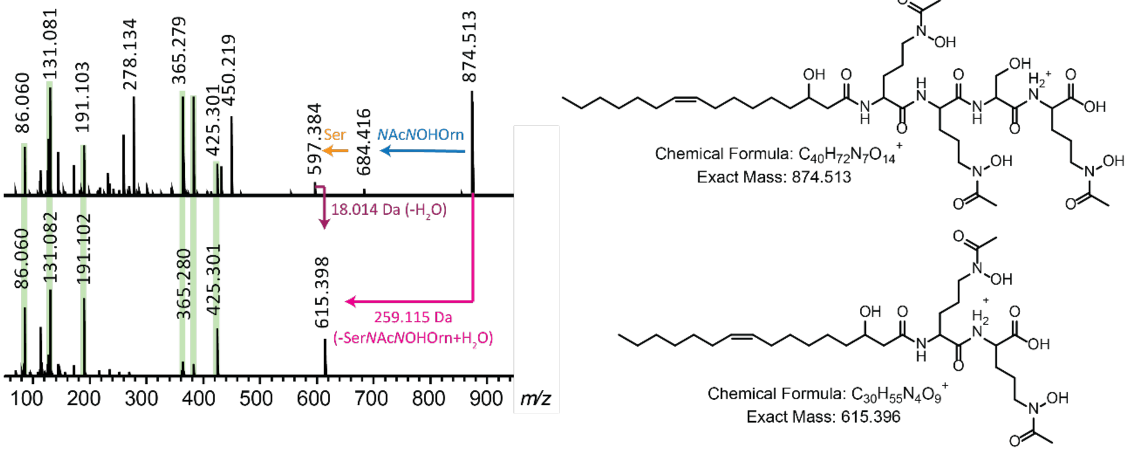

Amphibactin D

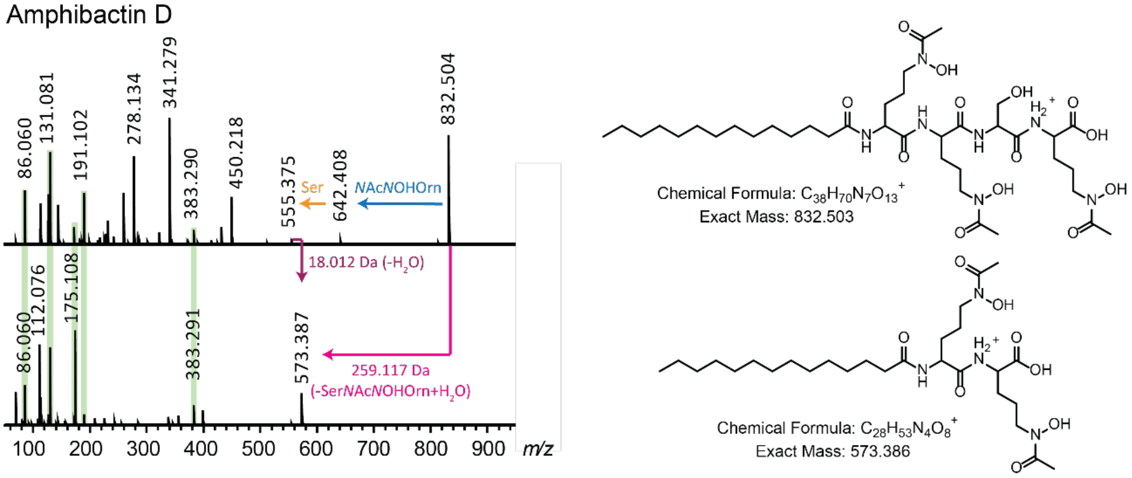

Amphibactin E

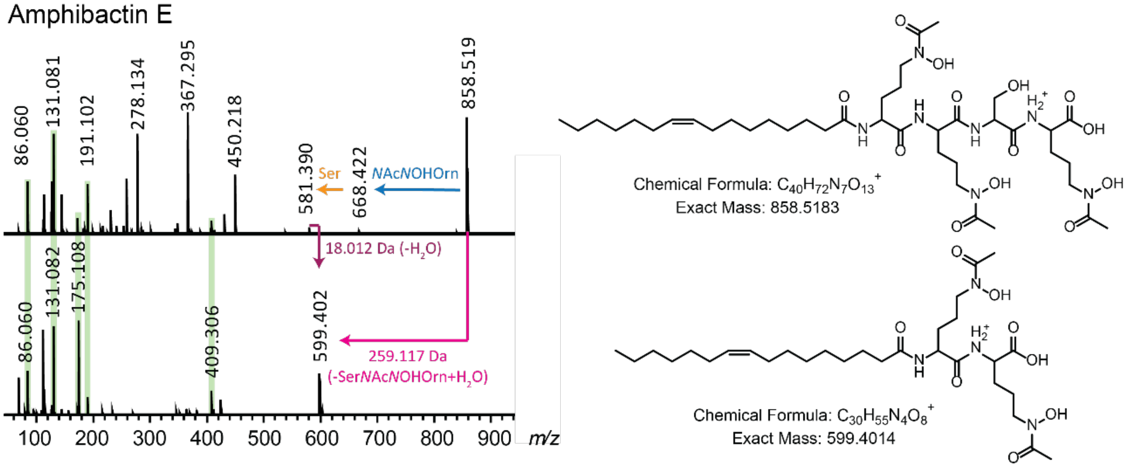

Amphibactin G

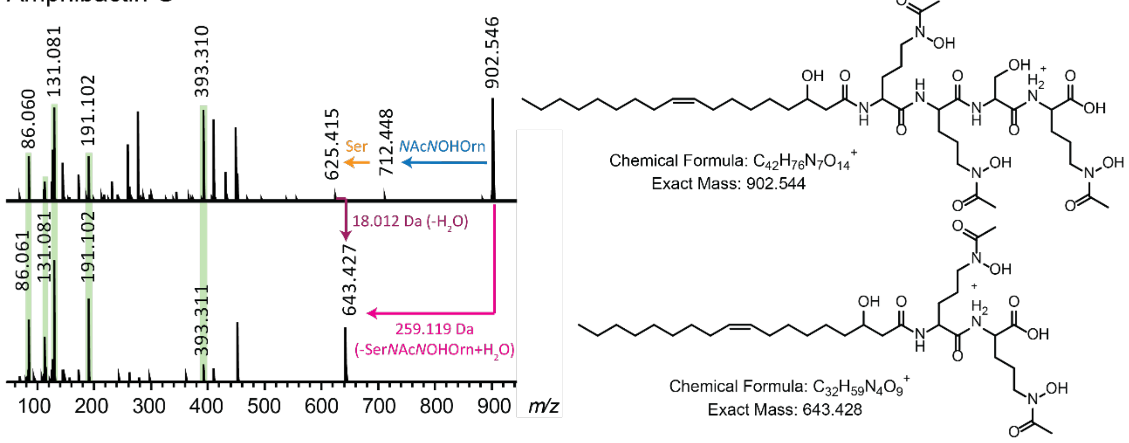

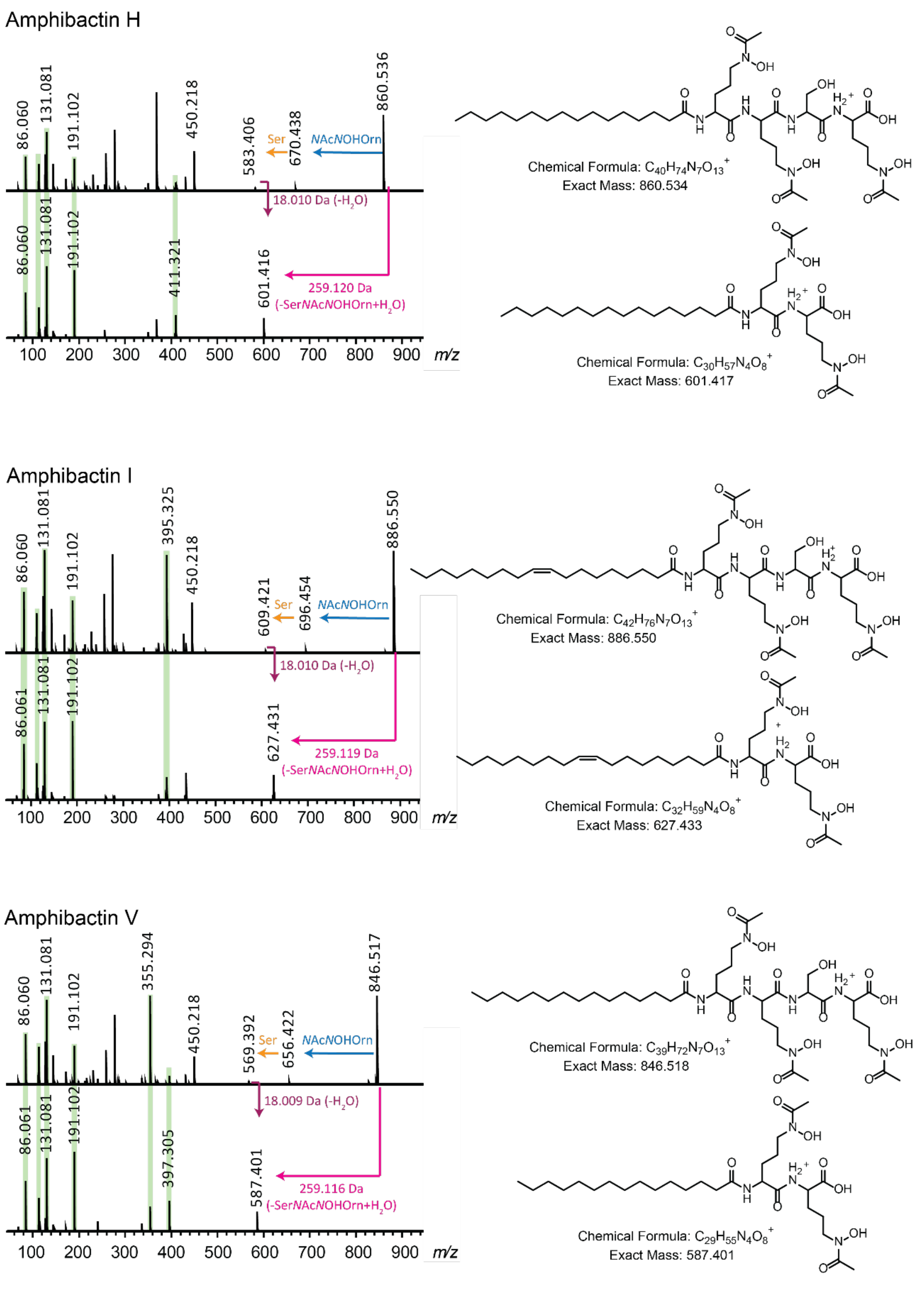

Figure S13. MS^2 spectral analysis of amphibactins and degradation products. The MS^2 mirror plots for amphibactin F, amphibactin B, amphibactin C, amphibactin D, amphibactin E, amphibactin G, amphibactin H, amphibactin I, amphibactin V, along with their respective degradation products are shown.

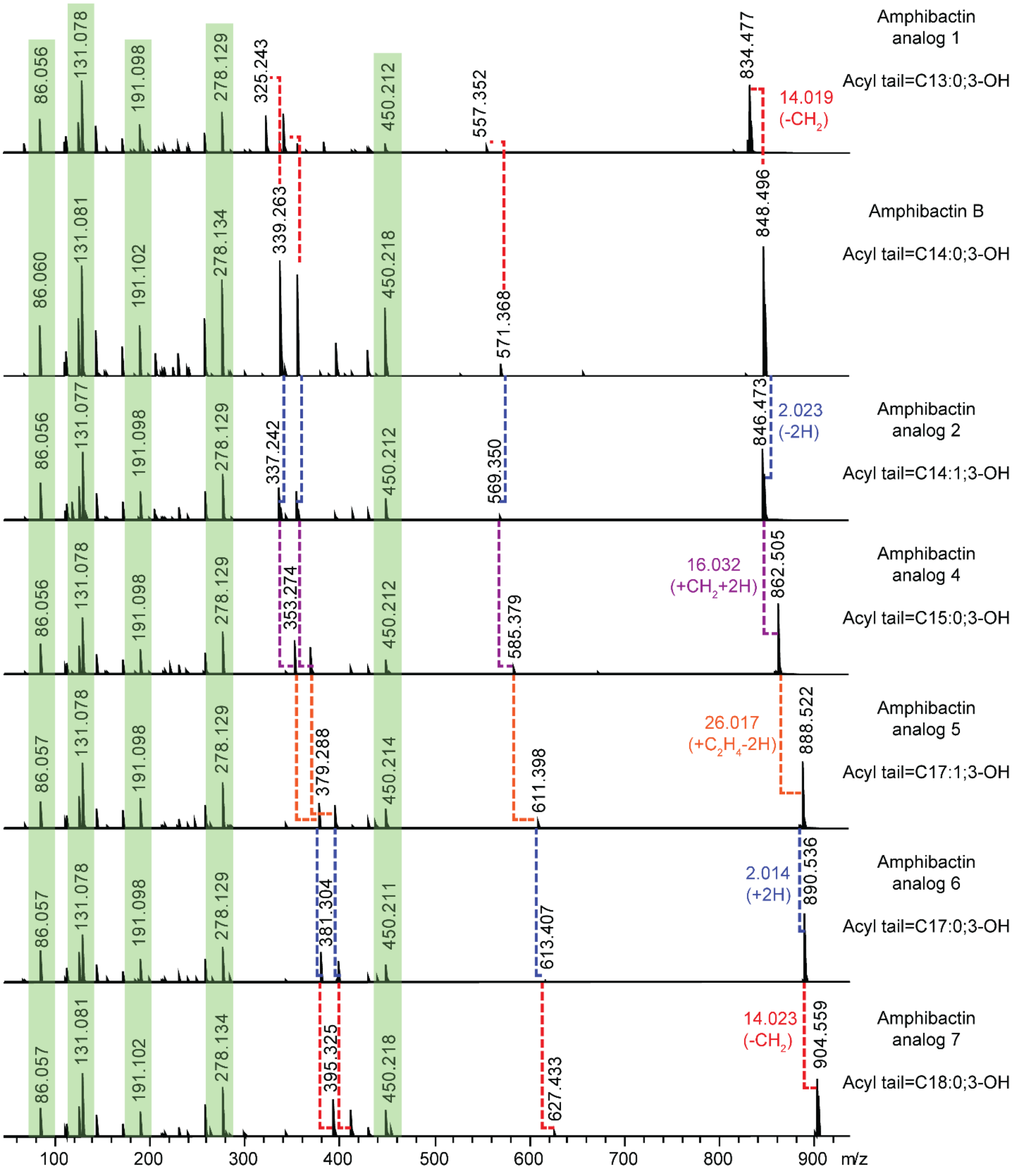

Figure S14. MS^2 spectral analysis of new amphibactin analogs, compared to amphibactin B. Conserved peaks are shown in green whereas mass shifts are shown in dashed lines. Amphibactin analog 3 was only detected in its degraded form and is therefore not shown here.

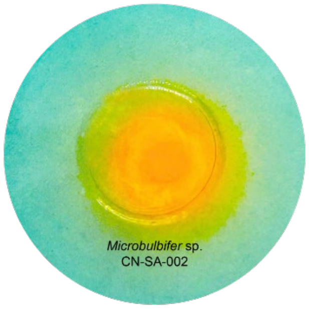

Figure S15. A petri plate showing iron acquisition by *Microbulbifer* sp. CNSA002 culture in O-CAS agar assay.

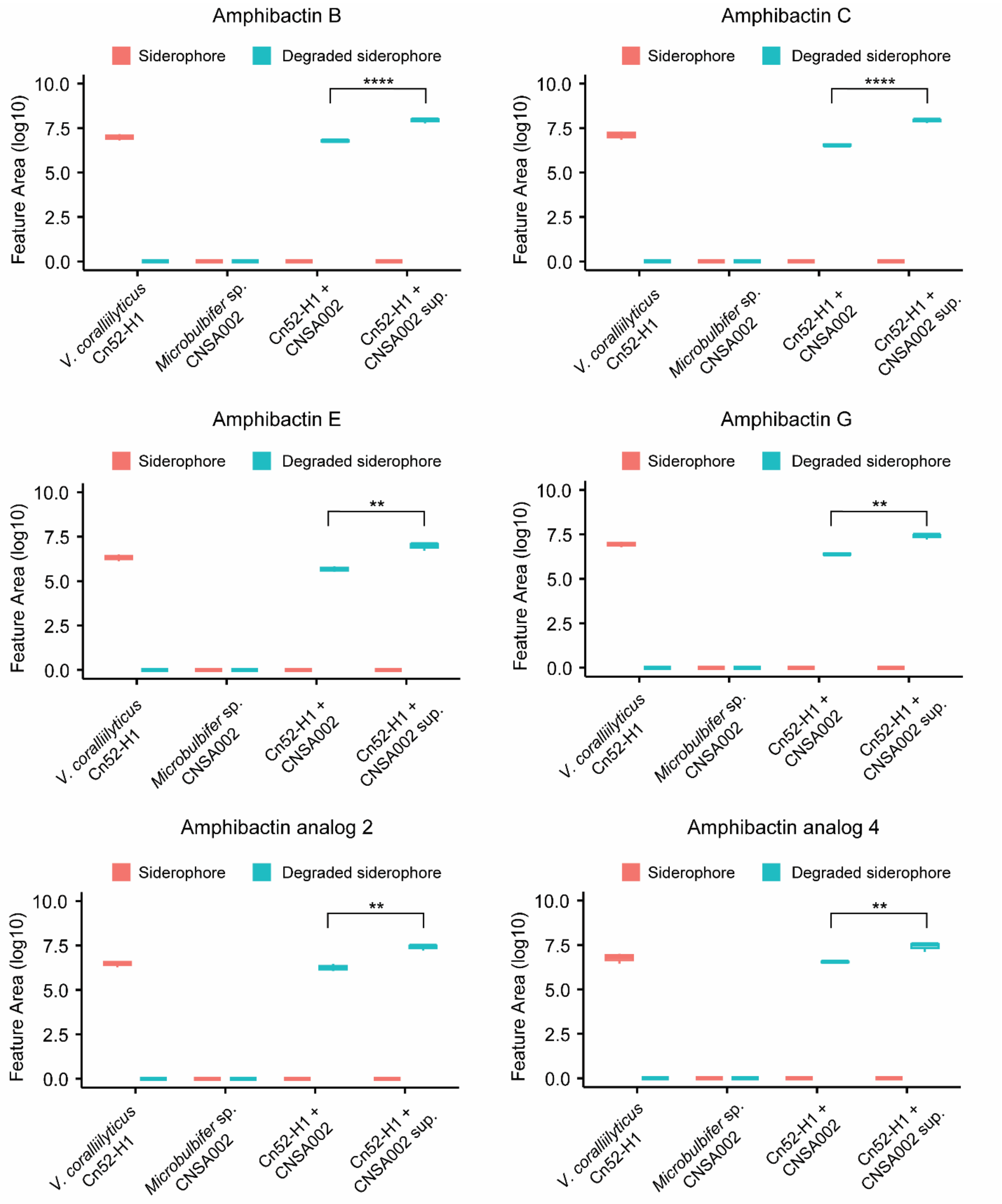

Figure S16. Detection of degraded amphibactins in *V. coralliilyticus* Cn52-H1 in coculture with *Microbulbifer* sp. CNSA002 and supplemented with *Microbulbifer* sp. CNSA002 supernatant. Boxplots are shown for the relative abundances of amphibactin B, C, E, G, analog 2 and analog 4 degradation products (*m/z* 589.381, 615.396, 599.401, 643.428, 587.365 and 603.396 respectively). Asterisks indicate significant differences between compared groups.

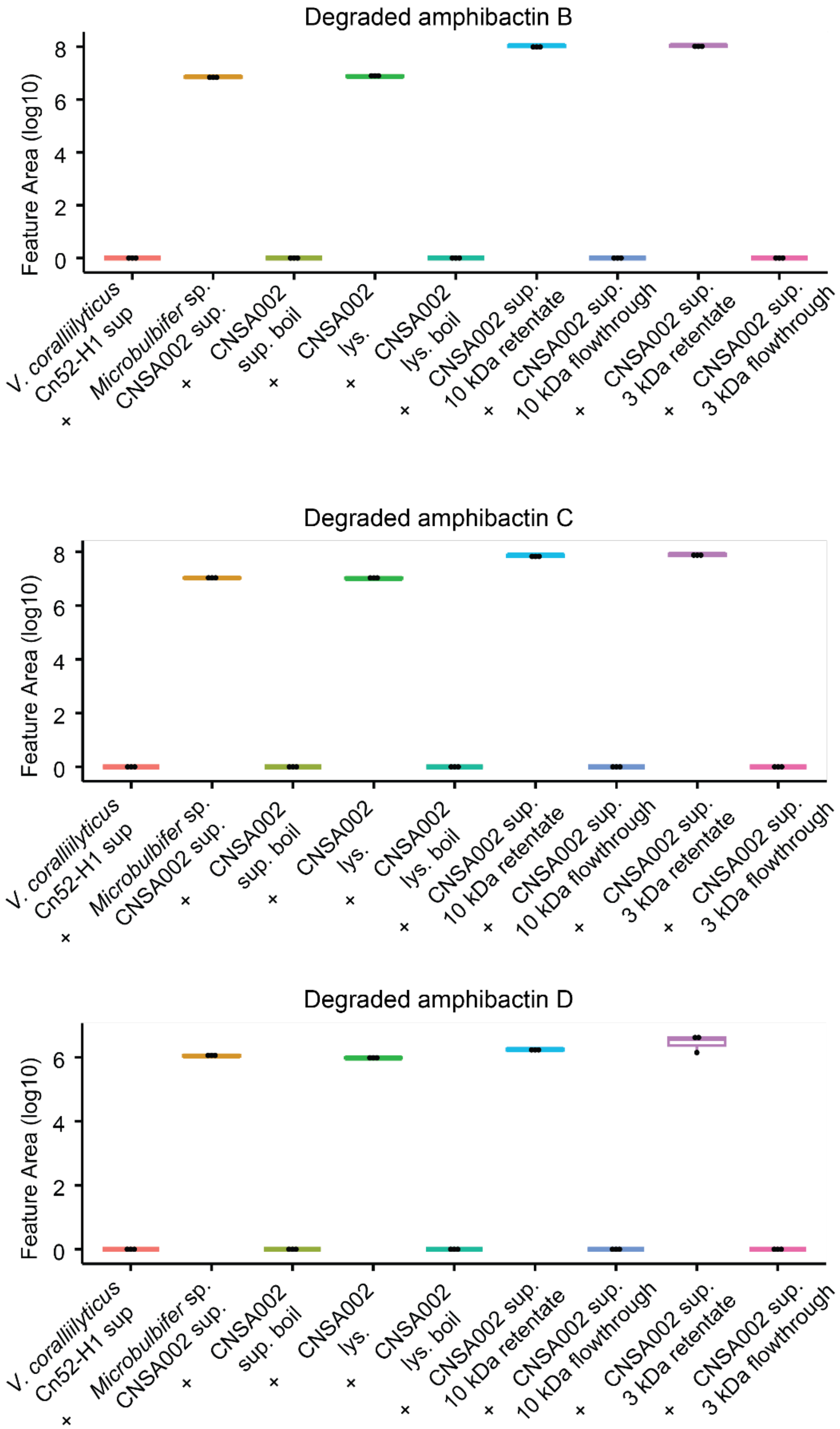

Figure S17. Detection of degraded amphibactins in *V. coralliilyticus* Cn52-H1 supernatant, supplemented with different *Microbulbifer* sp. CNSA002 fractions. Boxplots are shown for the

relative abundances of amphibactin B, C and D degradation products (*m/z* 589.381, 615.396 and 573.386 respectively)

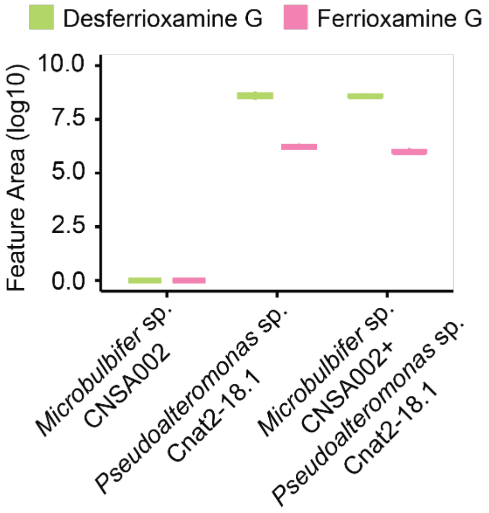

Figure S18. Boxplot of the relative abundances of desferrioxamine G and ferrioxamine G (619.366 *m/z* and 672.277 *m/z* respectively) in *Pseudoalteromonas* sp. Cnat2-18.1 and *Microbulbifer* sp. CNSA002 mono- and cocultures.

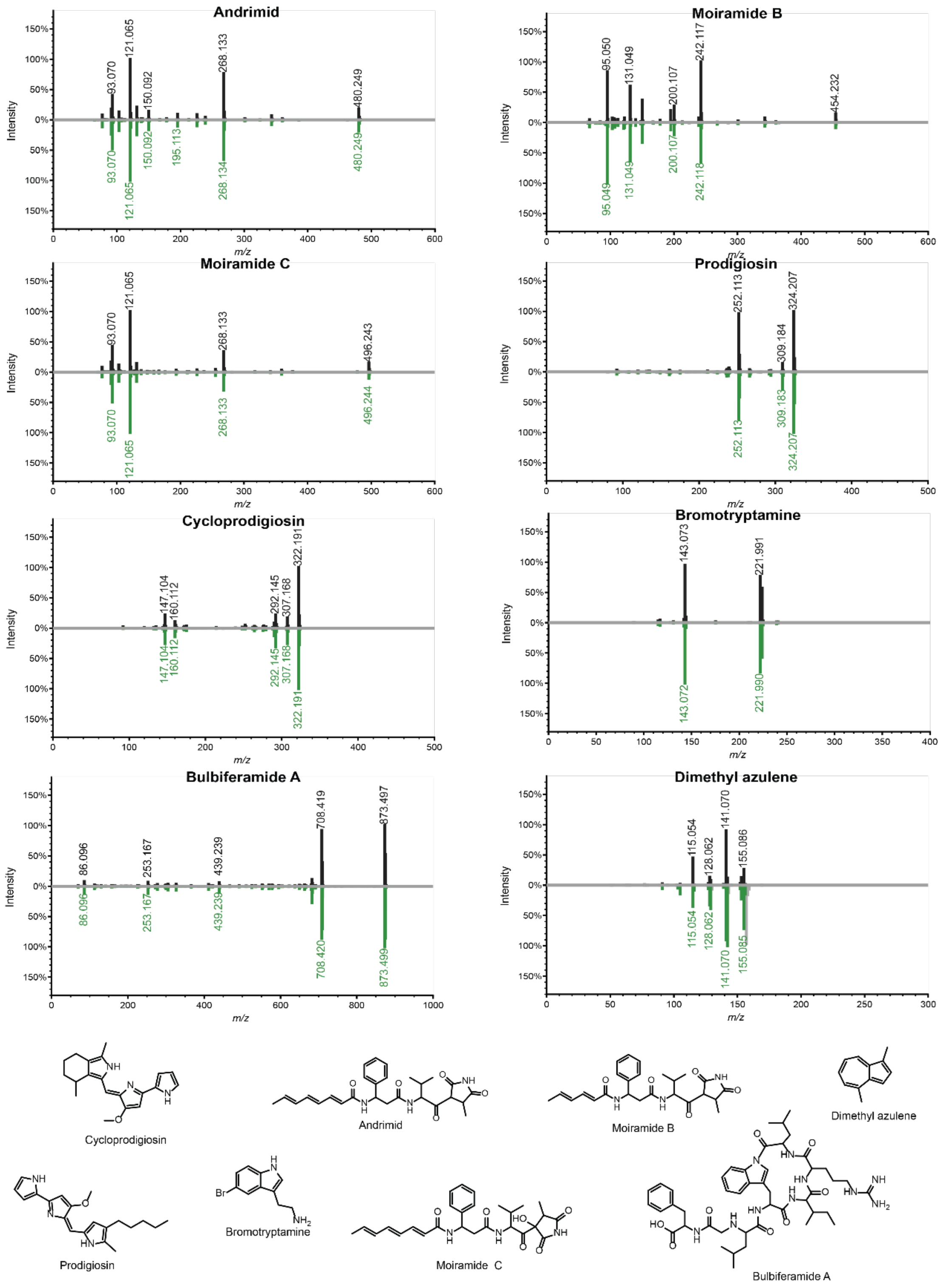

Figure S19. Mirror plots comparing experimental MS[2] spectra of natural products detected in this study with spectra deposited in the GNPS library.

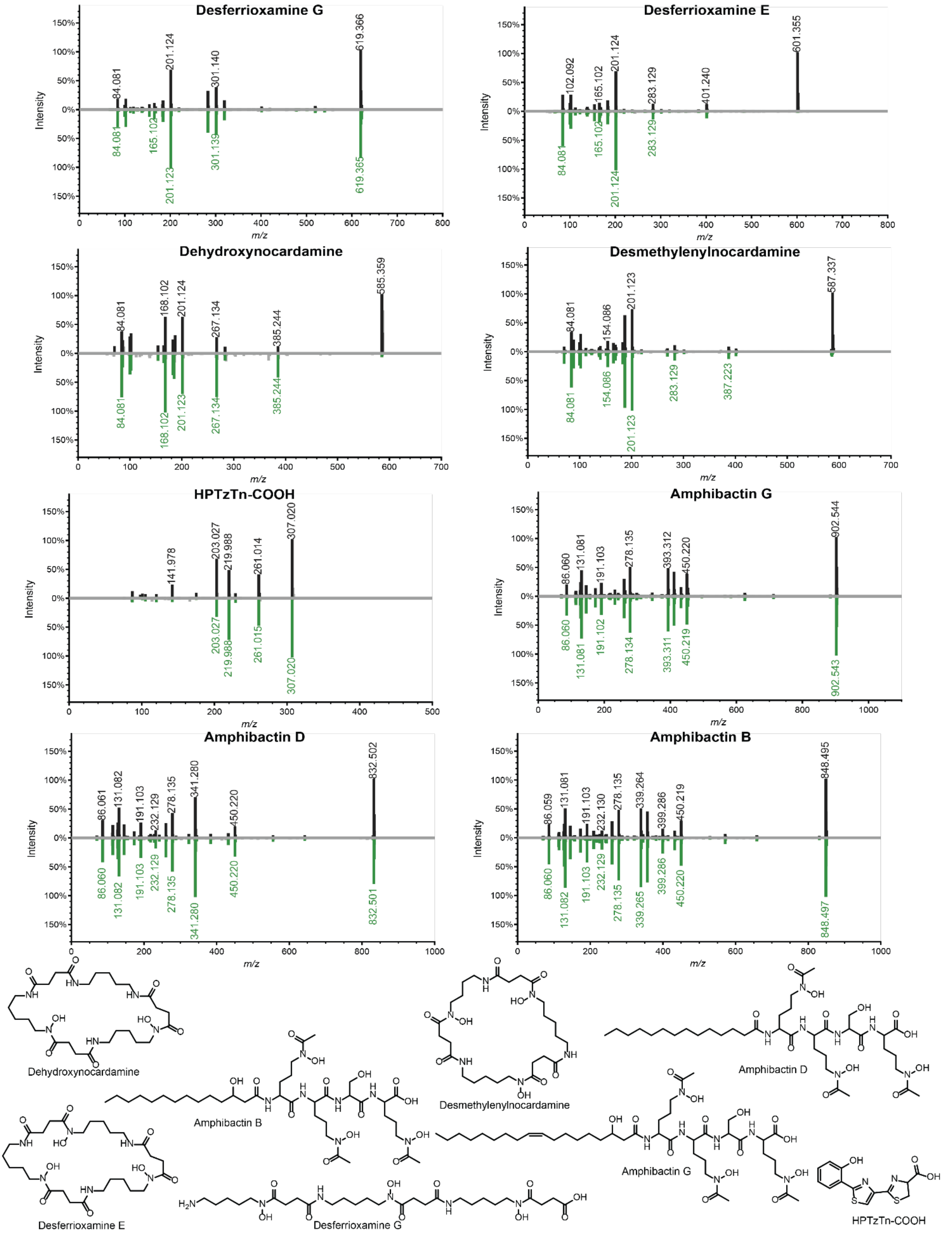

Figure S20. Mirror plots comparing experimental MS^2 spectra of hydroxamate siderophores and HPTzTn-COOH detected in this study with spectra deposited in the GNPS library.

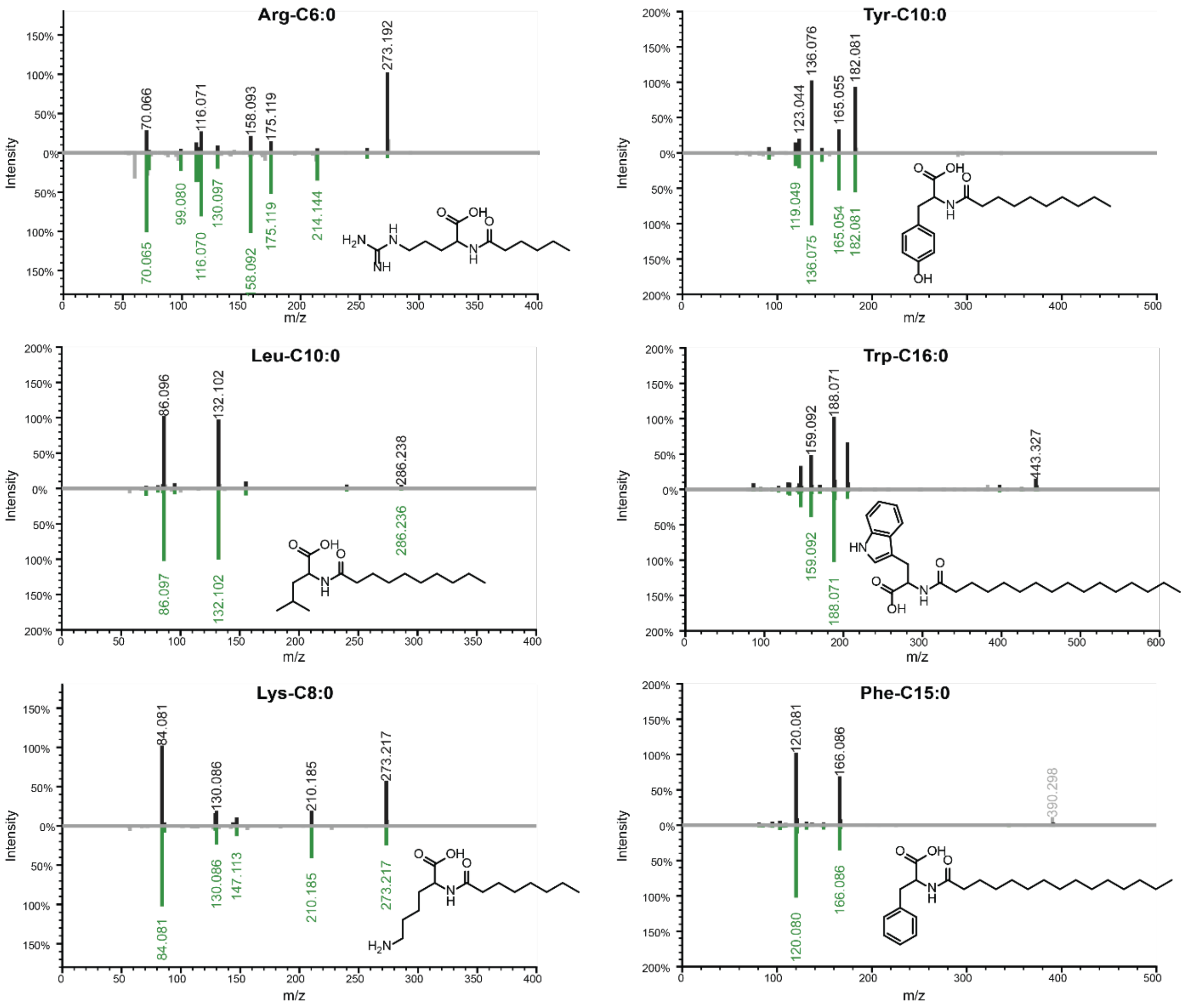

Figure S21. Mirror plots comparing experimental MS^2 spectra of *N*-acyl amides detected in this study with spectra deposited in the GNPS library.

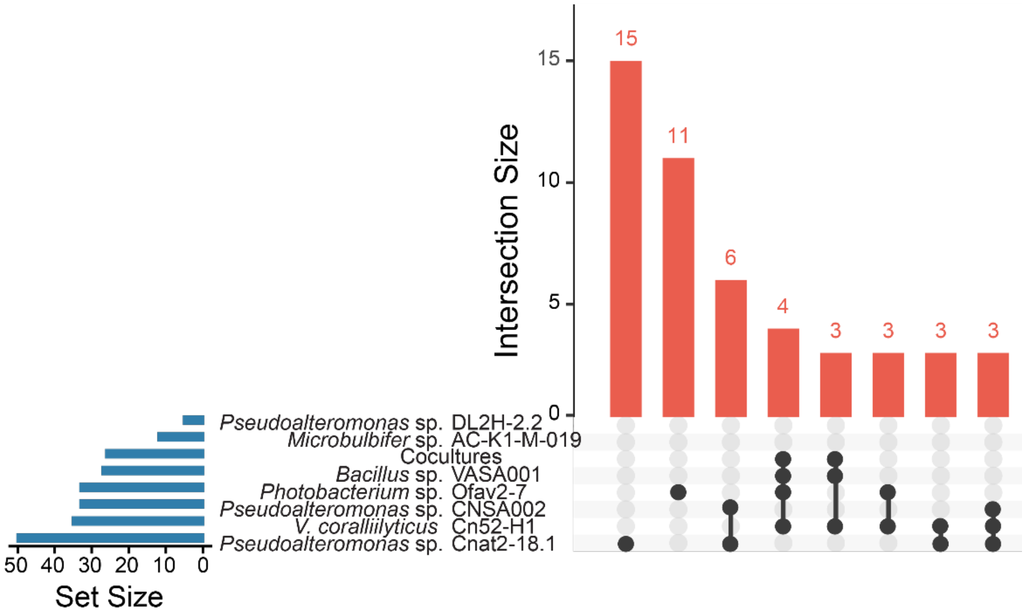

Figure S22. Upset plot for *N*-acyl amides detected in different mono- and cocultures. The top 8 intersections are shown for clarity.

Table S1. Putative annotation of top 50 features present in the *V. coralliilyticus* Cn52-H1 and *Microbulbifer* sp. CNSA002 coculture heatmap.

*m/z*_RT	Formula (M)	Adduct	Annotation/Compound class	Source
169.085_7.24	-	$[M+H]^+$	*N*-acyl amides	Annotation propagation
226.620_6.08	-	-	-	-
241.143_12.10	$C_{13}H_{20}O_4$	$[M+H]^+$	Fatty acyl	CANOPUS
250.647_5.26	-	-	-	-
253.131_6.08	$C_{11}H_{16}N_4O_3$	$[M+H]^+$	His-Pro	CANOPUS
253.689_7.40	-	-	-	-
262.137_6.06	$C_{22}H_{21}N_2O_3S$	$[M+H]^+$	Dipeptide	CANOPUS
267.686_7.39	-	-	-	-
283.238_10.92	$C_{16}H_{30}N_2O_2$	$[M+H]^+$	*N*-acyl amides	CANOPUS
284.197_4.81	$C_{14}H_{25}N_3O_3$	$[M+H]^+$	Proline and derivatives	CANOPUS
284.626_6.79	-	-	-	-
286.144_9.43	$C_{17}H_{19}NO_3$	$[M+H]^+$	Phe-C8:3	Annotation propagation
286.238_12.33	$C_{16}H_{31}NO_3$	$[M+H]^+$	Leu-C10:0	GNPS
287.681_7.36	-	-	Proline and derivatives	Annotation propagation
288.187_10.44	$C_{27}H_{50}N_4O_9$	$[M+2H]^{2+}$	Degraded amphibactin analog 1	Annotation propagation
288.679_5.64	-	-	*N*-acyl amides	Annotation propagation
294.679_5.96	-	-	Peptide	Annotation propagation
299.197_11.53	$C_{30}H_{53}N_4O_8$	$[M+2H]^{2+}$	Degraded amphibactin analog 3	CANOPUS
301.105_3.24	$C_{12}H_{16}N_2O_7$	$[M+H]^+$	Pyrimidine nucleoside	CANOPUS
307.176_7.09	$C_{15}H_{22}N_4O_3$	$[M+H]^+$	Arg-C9:4	CANOPUS
309.210_12.32	$C_{30}H_{56}N_4O_9$	$[M+2H]^{2+}$	Degraded amphibactin F	Annotation propagation
309.684_6.19	-	-	-	-
310.167_23.77	$C_{17}H_{27}NS_2$	$[M+H]^+$		SIRIUS
314.659_8.03	-	-	-	-
316.674_6.22	-	-	Peptide	Annotation propagation
320.160_4.80	$C_{16}H_{21}N_3O_4$	$[M+H]^+$	Phe-Gly-Pro	CANOPUS
328.183_12.32	$C_{11}H_{21}N_9O_3$	$[M+H]^+$	*N*-acyl amides	CANOPUS
329.254_8.49	$C_{16}H_{32}N_4O_3$	$[M+H]^+$	Arg-C10:0	GNPS
333.202_12.69	$C_{18}H_{30}O_4$	$[M+Na]^+$	-	SIRIUS
334.164_6.54	$C_{12}H_{24}N_5O_4P$	$[M+H]^+$	*N*-acyl amides	CANOPUS
334.183_12.15	$C_{12}H_{19}N_{11}O$	$[M+H]^+$	Dialkylarylamides	CANOPUS
337.189_12.32	$C_{21}H_{24}N_2O_2$	$[M+H]^+$	Amino acids and derivatives	CANOPUS
339.169_6.39	$C_{20}H_{22}N_2O_3$	$[M+H]^+$	*N*-acyl amides	CANOPUS
339.712_6.96	-	-	-	-
341.191_12.69	$C_{14}H_{24}N_6O_4$	$[M+H]^+$	*N*-acyl amides	CANOPUS
345.220_6.78	$C_{12}H_{26}N_9O_3$	$[M+H]^+$	Peptide	CANOPUS
351.220_7.19	$C_{14}H_{30}N_4O_6$	$[M+H]^+$	Peptide	CANOPUS

*m/z*_RT	Formula (M)	Adduct	Annotation/Compound class	Source
356.710_6.66	-	-	-	-
358.177_7.80	$C_{19}H_{23}N_3O_4$	$[M+H]^+$	Amino acids and derivatives	
365.684_6.26	-	-	-	-
378.713_7.75	-	-	-	-
421.281_7.40	$C_{22}H_{36}N_4O_4$	$[M+H]^+$	Cyclo-(Pro)$_2$-(Leu)$_2$	CANOPUS
427.233_6.71	$C_{23}H_{30}N_4O_4$	$[M+H]^+$	Peptide	CANOPUS
452.218_6.45	$C_{25}H_{29}N_3O_5$	$[M+H]^+$	Peptide	CANOPUS
473.286_5.96	$C_{24}H_{36}N_6O_4$	$[M+H]^+$	Oligopeptide	CANOPUS
488.286_5.13	$C_{25}H_{37}N_5O_5$	$[M+H]^+$	(Pro)$_2$-Lys-Phe	CANOPUS
523.266_6.09	$C_{27}H_{34}N_6O_5$	$[M+H]^+$	Oligopeptide	CANOPUS
602.265_7.30	$C_{29}H_{39}N_5O_7S$	$[M+H]^+$	Met-(Ala)$_2$-Phe-Tyr	CANOPUS
677.330_6.39	$C_{35}H_{44}N_6O_8$	$[M+H]^+$	(Pro)$_3$-Gly-Phe-Tyr	CANOPUS
746.355_5.07	$C_{43}H_{47}N_5O_7$	$[M+H]^+$	Oligopeptide	CANOPUS

Table S2. Putative annotation of 76 features uniquely detected in marine bacteria cocultures with *V. coralliilyticus* Cn52-H1.

*m/z*_RT	Formula (M)	Adduct	Annotation/ Compound class	Source	GNPS Analog	Samples
283.118_9.67	$C_{14}H_{18}O_6$	$[M+H]^+$	Benzenoids	CANOPUS	-	Cn52-H1+Cnat2-18.1
285.290_9.55	$C_{17}H_{36}N_2O$	$[M+H]^+$	Fatty amide	CANOPUS	-	Cn52-H1+AC-K1-M-019
286.189_11.16	-	-	-	-	-	Cn52-H1+CNSA002
287.086_10.37	-	-	Ethylphenol substructure	MS2LDA	17-*epi*-methandienone	Cn52-H1+Ofav2-7
287.204_14.50	$C_{16}H_{30}O_2S$	$[M+H]^+$	Dialkylthioether	CANOPUS	-	Cn52-H1+Ofav2-7
289.170_8.20	$C_{20}H_{20}N_2$	$[M+H]^+$	Alkylindole	CANOPUS	MMV676539 (*N*-[2-(1*H*-Indol-3-yl)ethyl]-2-oxo-2*H*-1-benzopyran-3-carboxamide)	Cn52-H1+AC-K1-M-019
294.186_10.50	$C_{28}H_{50}N_4O_9$	$[M+2H]^{2+}$	Degraded amphibactin analog 1	MS2LDA	-	Cn52-H1+CNSA002
300.205_12.47	$C_{30}H_{54}N_4O_8$	$[M+2H]^{2+}$	Degraded amphibactin E	MS2LDA	-	Cn52-H1+CNSA002
301.213_13.38	$C_{30}H_{56}N_4O_8$	$[M+2H]^{2+}$	Putative degraded amphibactin H	MS2LDA	-	Cn52-H1+CNSA002
308.202_11.61	$C_{30}H_{54}N_4O_9$	$[M+2H]^{2+}$	Degraded amphibactin C	MS2LDA	-	Cn52-H1+CNSA002
316.218_12.99	$C_{31}H_{58}N_4O_9$	$[M+2H]^{2+}$	Degraded amphibactin analog 4	MS2LDA	-	Cn52-H1+CNSA002
320.259_10.10	$C_{20}H_{33}NO_2$	$[M+H]^+$	*N*-acyl amines	CANOPUS	PABA-C17:1	Cn52-H1+Ofav2-7
322.218_12.79	$C_{32}H_{58}N_4O_9$	$[M+2H]^{2+}$	Degraded amphibactin G	MS2LDA	-	Cn52-H1+CNSA002
336.158_10.44	$C_{14}H_{26}NO_6P$	$[M+H]^+$	-	SIRIUS	-	Cn52-H1+Cnat2-18.1
342.189_11.93	-	-	-	-	-	Cn52-H1+Cnat2-18.1
344.186_19.76	-	-	-	-	-	Cn52-H1+DL2H-2.2
346.219_12.51	$C_{24}H_{27}NO$	$[M+H]^+$	Benzenoids	CANOPUS	-	Cn52-H1+AC-K1-M-019

*m/z*_RT	Formula (M)	Adduct	Annotation/ Compound class	Source	GNPS Analog	Samples
349.248_9.71	$C_{20}H_{32}N_2O_3$	$[M+H]^+$	Amino acids and derivatives	CANOPUS	-	Cn52-H1+VASA001
351.264_14.14	$C_{20}H_{34}N_2O_3$	$[M+H]^+$	-	SIRIUS	-	Cn52-H1+Cnat2-18.1
362.298_23.97	-	-	-	-	-	Cn52-H1+Cnat2-18.1,Cn52-H1+CNSA002
365.303_18.63	$C_{21}H_{42}O_3$	$[M+Na]^+$	-	SIRIUS	-	Cn52-H1+Cnat2-18.1
377.280_11.15	$C_{22}H_{36}N_2O_3$	$[M+H]^+$	Amino acids and derivatives	CANOPUS	-	Cn52-H1+VASA001
383.322_23.92	-	-	-	-	-	Cn52-H1+Cnat2-18.1
387.182_11.26	$C_{22}H_{26}O_6$	$[M+H]^+$	Benzenoids	CANOPUS	-	Cn52-H1+Cnat2-18.1
396.182_18.13	$C_{23}H_{25}NO_5$	$[M+H]^+$	Piperidine	CANOPUS	-	Cn52-H1+Cnat2-18.1
398.766_12.39	-	-	Unannotated motifs 547, 656, 661	-	-	Cn52-H1+AC-K1-M-019
399.695_8.63	-	-	-	-	-	Cn52-H1+Cnat2-18.1
401.270_11.25	$C_{14}H_{36}N_6O_7$	$[M+H]^+$	Amino acids and derivatives	FBMN	-	Cn52-H1+Cnat2-18.1
406.703_9.27			Alkylamine substructure	MS2LDA	-	Cn52-H1+Cnat2-18.1
409.192_15.37	$C_{27}H_{24}N_2O_2$	$[M+H]^+$	Alkyl indole	CANOPUS	Ajmalicine	Cn52-H1+Cnat2-18.1
411.322_18.49	-	-	Unannotated motifs 552, 656	-	-	Cn52-H1+AC-K1-M-019
418.283_17.68	$C_{22}H_{35}N_5O_3$	$[M+H]^+$	Cyclohexylamines	CANOPUS	-	Cn52-H1+AC-K1-M-019
422.294_12.98	$C_{13}H_{39}N_7O_8$	$[M+H]^+$	Amino acids and derivatives	FBMN	-	Cn52-H1+Cnat2-18.1
429.302_13.59	$C_{16}H_{40}N_6O_7$	$[M+H]^+$	Amino acids and derivatives	FBMN	-	Cn52-H1+Cnat2-18.1
432.194_16.64	$C_{25}H_{25}N_3O_4$	$[M+H]^+$	Benzodiazine	CANOPUS	-	Cn52-H1+Cnat2-18.1
433.265_11.98	$C_{31}H_{32}N_2$	$[M+H]^+$	Phenyl piperidine	CANOPUS	Acetamide, *N*-[3-(acetyloxy)-22-oxo-16,23-cyclocholesta-5,16(23)-dien-26-yl]- (9CI)	Cn52-H1+AC-K1-M-019

*m/z*_RT	Formula (M)	Adduct	Annotation/ Compound class	Source	GNPS Analog	Samples
435.302_13.30	$C_{17}H_{38}N_8O_5$	$[M+H]^+$	Amino acids and derivatives	FBMN	-	Cn52-H1+Cnat2-18.1
436.309_14.16	$C_{14}H_{41}N_7O_8$	$[M+H]^+$	Amino acids and derivatives	FBMN	-	Cn52-H1+Cnat2-18.1,Cn52-H1+AC-K1-M-019
442.310_13.85	$C_{15}H_{39}N_9O_6$	$[M+H]^+$	Amino acids and derivatives	CANOPUS	-	Cn52-H1+Cnat2-18.1
448.358_19.66	$C_{31}H_{45}NO$	$[M+H]^+$	Benzenoids	CANOPUS	-	Cn52-H1+Ofav2-7
448.749_13.05	-	-	-	-	-	Cn52-H1+Cnat2-18.1
456.406_19.66	$C_{27}H_{53}NO_3$	$[M+O+H]^+$	Ceramide	CANOPUS	-	Cn52-H1+Cnat2-18.1
462.765_14.38	-	-	-	-	-	Cn52-H1+Cnat2-18.1
468.765_13.99	-	-	-	-	-	Cn52-H1+Cnat2-18.1
470.179_8.62	$C_{19}H_{28}N_5O_7P$	$[M+H]^+$	Amino acids and derivatives	CANOPUS	-	Cn52-H1+Cnat2-18.1
470.179_8.88	$C_{21}H_{23}N_7O_6$	$[M+H]^+$	Amino acids and derivatives	CANOPUS	-	Cn52-H1+Cnat2-18.1
472.358_16.83	$C_{33}H_{45}NO$	$[M+H]^+$	Steroid	CANOPUS	Cyclopamine	Cn52-H1+AC-K1-M-019
491.288_8.43	-	-	Leucine substructure	MS2LDA	-	Cn52-H1+Cnat2-18.1
509.843_12.54	-	-	Unannotated motifs 398, 552, 656, 661	-	-	Cn52-H1+AC-K1-M-019
516.327_16.89	$C_{37}H_{41}NO$	$[M+H]^+$	Benzenoids	CANOPUS	-	Cn52-H1+AC-K1-M-019
518.342_16.71	-	-	-	-	-	Cn52-H1+Cnat2-18.1,Cn52-H1+AC-K1-M-019
542.400_17.66	$C_{37}H_{51}NO_2$	$[M+H]^+$	Benzenoids	CANOPUS	-	Cn52-H1+AC-K1-M-019
544.416_19.45	$C_{37}H_{53}NO_2$	$[M+H]^+$	Steroid	CANOPUS	-	Cn52-H1+AC-K1-M-019
545.411_9.56	$C_{36}H_{52}N_2O_2$	$[M+H]^+$	Steroid	CANOPUS	-	Cn52-H1+AC-K1-M-019
573.311_20.36	-	-	-	-	-	Cn52-H1+Ofav2-7,Cn52-H1+Cnat2-18.1
573.386_12.06	$C_{28}H_{52}N_4O_8$	$[M+H]^+$	Degraded amphibactin D	Annotation propagation	-	Cn52-H1+CNSA002

*m/z*_RT	Formula (M)	Adduct	Annotation/ Compound class	Source	GNPS Analog	Samples
587.366_10.50	$C_{28}H_{50}N_4O_9$	$[M+H]^+$	Degraded amphibactin analog 1	Annotation propagation	-	Cn52-H1+CNSA002
587.401_12.70	$C_{29}H_{54}N_4O_8$	$[M+H]^+$	Degraded amphibactin V	Annotation propagation	-	Cn52-H1+CNSA002
589.381_11.11	$C_{28}H_{52}N_4O_9$	$[M+H]^+$	Degraded amphibactin B	Annotation propagation	-	Cn52-H1+CNSA002
599.402_12.48	$C_{30}H_{54}N_4O_8$	$[M+H]^+$	Degraded amphibactin E	Annotation propagation	-	Cn52-H1+CNSA002
601.417_12.25	$C_{30}H_{56}N_4O_8$	$[M+H]^+$	Putative degraded amphibactin H	Annotation propagation	-	Cn52-H1+CNSA002
601.417_13.36	$C_{30}H_{56}N_4O_8$	$[M+H]^+$	Putative degraded amphibactin H	Annotation propagation	-	Cn52-H1+CNSA002
603.397_11.78	$C_{29}H_{54}N_4O_9$	$[M+H]^+$	Degraded amphibactin analog 2	Annotation propagation	-	Cn52-H1+CNSA002
612.410_11.77	-	-	Unannotated motifs 547, 567	-	-	Cn52-H1+AC-K1-M-019
615.397_11.62	$C_{30}H_{54}N_4O_9$	$[M+H]^+$	Degraded amphibactin C	Annotation propagation	-	Cn52-H1+CNSA002
617.412_12.42	$C_{30}H_{56}N_4O_9$	$[M+H]^+$	Degraded amphibactin F	NMR	-	Cn52-H1+CNSA002
627.433_12.64	$C_{32}H_{58}N_4O_8$	$[M+H]^+$	Putative degraded amphibactin I	Annotation propagation	Desferrichrome	Cn52-H1+CNSA002
627.433_13.70	$C_{32}H_{58}N_4O_8$	$[M+H]^+$	Putative degraded amphibactin I	Annotation propagation	-	Cn52-H1+CNSA002
629.412_12.24	$C_{31}H_{56}N_4O_9$	$[M+H]^+$	Degraded amphibactin analog 3	Annotation propagation	-	Cn52-H1+CNSA002
631.427_12.98	$C_{31}H_{58}N_4O_9$	$[M+H]^+$	Degraded amphibactin analog 4	Annotation propagation	-	Cn52-H1+CNSA002
643.427_12.80	$C_{32}H_{58}N_4O_9$	$[M+H]^+$	Degraded amphibactin G	Annotation propagation	-	Cn52-H1+CNSA002
643.499_18.74	-	-	-	-	-	Cn52-H1+AC-K1-M-019

*m/z*_RT	Formula (M)	Adduct	Annotation/ Compound class	Source	GNPS Analog	Samples
728.303_5.64	-	-	-	-	-	Cn52-H1+Cnat2-18.1
790.472_16.17	$C_{56}H_{59}N_3O$	$[M+H]^+$	Benzenoids	CANOPUS	-	Cn52-H1+AC-K1-M-019
792.486_16.54	$C_{56}H_{61}N_3O$	$[M+H]^+$	-	-	-	Cn52-H1+AC-K1-M-019
794.502_17.01	-	-	-	-	-	Cn52-H1+AC-K1-M-019

Table S3. List of annotated metabolites detected in this study.

Compound	Adduct	Theoretical *m/z*	Experimental *m/z*	ppm error	Formula (M)	Source
Dimethyl azulene	$[M+H]^+$	157.101	157.101	0	$C_{12}H_{12}$	CANOPUS
Dimethylvinyl azulene	$[M+H]^+$	183.117	183.116	5.5	$C_{14}H_{14}$	Annotation propagation
Methyl divinyl azulene	$[M+H]^+$	195.117	195.117	0	$C_{15}H_{14}$	Annotation propagation
Ethylmethyl azulenecarbaldehyde	$[M+H]^+$	199.112	199.112	0	$C_{14}H_{16}O_2$	Annotation propagation
Dimethyl propenyl tetrahydroazulene	$[M+H]^+$	201.164	201.164	0	$C_{15}H_{20}$	Annotation propagation
Bromotryptamine	$[M+H]^+$	239.018	239.018	0	$C_{10}H_8BrN$	GNPS
N-formyl bromotryptamine	$[M+H]^+$	267.013	267.013	0	$C_{11}H_{11}BrN_2O$	Annotation propagation
I-acetyl bromotryptamine	$[M+H]^+$	281.028	281.028	0	$C_{12}H_{13}BrN_2O$	Annotation propagation
HPTzTn-COOH	$[M+H]^+$	307.021	307.021	0	$C_{13}H_{10}N_2O_3S_2$	GNPS
Cycloprodigiosin-CH_2	$[M+H]^+$	308.176	308.176	0	$C_{19}H_{21}N_3O$	Annotation propagation
Cycloprodigiosin-CH_2+2H	$[M+H]^+$	310.191	310.192	3.2	$C_{19}H_{23}N_3O$	Annotation propagation
Cycloprodigiosin	$[M+H]^+$	322.191	322.191	0	$C_{20}H_{23}N_3O$	GNPS
Prodigiosin	$[M+H]^+$	324.207	324.207	0	$C_{18}H_{27}N_3O$	GNPS
Cycloprodigiosin+CH_2	$[M+H]^+$	336.207	336.207	0	$C_{21}H_{25}N_3O$	Annotation propagation
Methyl hexyl prodiginine	$[M+H]^+$	338.223	338.223	0	$C_{21}H_{27}N_3O$	Annotation propagation
Cycloprodigiosin+C_2H_4	$[M+H]^+$	350.223	350.223	0	$C_{22}H_{27}N_3O$	Annotation propagation
Heptylprodigiosin	$[M+H]^+$	352.238	352.239	2.8	$C_{22}H_{29}N_3O$	Annotation propagation
Moiramide B	$[M+H]^+$	454.234	454.234	0	$C_{25}H_{31}N_3O_5$	GNPS
Andrimid	$[M+H]^+$	480.249	480.250	1.5	$C_{27}H_{33}N_3O_5$	GNPS
Andrimid+2H	$[M+H]^+$	482.265	482.265	0	$C_{27}H_{35}N_3O_5$	GNPS
Moiramide C	$[M+H]^+$	496.244	496.245	2	$C_{27}H_{33}N_3O_6$	GNPS
Degraded amphibactin D	$[M+H]^+$	573.386	573.386	0	$C_{28}H_{52}N_4O_8$	Annotation propagation
Degarded amphibactin analog 1	$[M+H]^+$	575.365	575.365	0	$C_{27}H_{50}N_4O_9$	Annotation propagation
Dehydroxynocardamine	$[M+H]^+$	585.361	585.359	3.4	$C_{27}H_{48}N_6O_8$	GNPS
Desmethylenylnocardamine	$[M+H]^+$	587.340	587.340	0	$C_{26}H_{46}N_6O_9$	GNPS
Degraded amphibactin analog 2	$[M+H]^+$	587.365	587.366	1.7	$C_{28}H_{50}N_4O_9$	Annotation propagation
Degraded amphibactin V	$[M+H]^+$	587.401	587.401	0	$C_{29}H_{54}N_4O_8$	Annotation propagation
Degraded amphibactin B	$[M+H]^+$	589.381	589.381	0	$C_{28}H_{52}N_4O_9$	Annotation propagation
Degraded amphibactin analog 3	$[M+H]^+$	597.386	597.386	0	$C_{30}H_{53}N_4O_8$	Annotation propagation
Degraded amphibactin E	$[M+H]^+$	599.401	599.402	1.7	$C_{30}H_{54}N_4O_8$	Annotation propagation
Desferrioxamine E	$[M+H]^+$	601.356	601.355	1.7	$C_{27}H_{48}N_6O_9$	GNPS
Degraded amphibactin H	$[M+H]^+$	601.417	601.417	0	$C_{30}H_{56}N_4O_8$	Annotation propagation
Degraded amphibactin analog 4	$[M+H]^+$	603.396	603.397	0	$C_{29}H_{54}N_4O_9$	Annotation propagation
Degraded amphibactin C	$[M+H]^+$	615.396	615.397	1.6	$C_{30}H_{54}N_4O_9$	Annotation propagation
Degraded amphibactin F	$[M+H]^+$	617.412	617.412	0	$C_{30}H_{56}N_4O_9$	NMR
Desferrioxamine G	$[M+H]^+$	619.366	619.366	0	$C_{27}H_{50}N_6O_{10}$	GNPS

Compound	Adduct	Theoretical *m/z*	Experimental *m/z*	ppm error	Formula (M)	Source
Degraded amphibactin I	$[M+H]^+$	627.433	627.433	0	$C_{32}H_{58}N_4O_8$	Annotation propagation
Degraded amphibactin analog 5	$[M+H]^+$	629.412	629.412	0	$C_{31}H_{56}N_4O_9$	Annotation propagation
Degraded amphibactin analog 6	$[M+H]^+$	631.428	631.427	1.6	$C_{31}H_{58}N_4O_9$	Annotation propagation
Degraded amphibactin G	$[M+H]^+$	643.428	643.427	1.6	$C_{32}H_{58}N_4O_9$	Annotation propagation
Degraded amphibactin analog 7	$[M+H]^+$	645.443	645.443	0	$C_{32}H_{60}N_4O_9$	Annotation propagation
Amphibactin D	$[M+H]^+$	832.503	832.501	1.9	$C_{38}H_{69}N_7O_{13}$	GNPS
Amphibactin analog 1	$[M+H]^+$	834.477	834.482	6.0	$C_{37}H_{67}N_7O_{14}$	Annotation propagation
Amphibactin B analog	$[M+H]^+$	846.482	846.481	1.2	$C_{37}H_{67}N_{11}O_{10}$	GNPS
Amphibactin analog 2	$[M+H]^+$	846.482	846.481	1.2	$C_{37}H_{67}N_{11}O_{10}$	Annotation propagation
Amphibactin V	$[M+H]^+$	846.518	846.516	2.7	$C_{39}H_{71}N_7O_{13}$	Annotation propagation
Amphibactin B	$[M+H]^+$	848.498	848.496	1.8	$C_{38}H_{69}N_7O_{14}$	GNPS
Amphibactin E	$[M+H]^+$	858.518	858.517	1.5	$C_{40}H_{71}N_7O_{13}$	Annotation propagation
Amphibactin H	$[M+H]^+$	860.534	860.531	3.4	$C_{40}H_{73}N_7O_{13}$	Annotation propagation
Amphibactin analog 4	$[M+H]^+$	862.513	862.512	1.4	$C_{39}H_{71}N_7O_{14}$	Annotation propagation
Bulbiferamide A	$[M+H]^+$	873.500	873.497	3.4	$C_{45}H_{64}N_{10}O_8$	GNPS
Amphibactin C	$[M+H]^+$	874.513	874.513	0.2	$C_{40}H_{71}N_7O_{14}$	Annotation propagation
Amphibactin F	$[M+H]^+$	876.529	876.527	2.1	$C_{40}H_{73}N_7O_{14}$	Annotation propagation
Fe-Amphibactin D	$[M+Fe-2H]^+$	885.414	885.413	1.1	$C_{38}H_{69}N_7O_{13}$	Annotation propagation
Amphibactin I	$[M+H]^+$	886.550	886.548	1.8	$C_{42}H_{75}N_7O_{13}$	Annotation propagation
Amphibactin analog 5	$[M+H]^+$	888.528	888.528	0.5	$C_{41}H_{73}N_7O_{14}$	Annotation propagation
Amphibactin analog 6	$[M+H]^+$	890.544	890.547	3.7	$C_{41}H_{75}N_7O_{14}$	Annotation propagation
Fe-Amphibactin analog 1	$[M+Fe-2H]^+$	899.393	899.392	1.1	$C_{37}H_{67}N_{11}O_{10}$	Annotation propagation
Fe-Amphibactin V	$[M+Fe-2H]^+$	899.429	899.429	0	$C_{39}H_{71}N_7O_{13}$	Annotation propagation
Amphibactin G	$[M+H]^+$	902.545	902.543	1.7	$C_{42}H_{79}N_7O_{14}$	GNPS
Amphibactin analog 7	$[M+H]^+$	904.560	904.563	3.4	$C_{42}H_{77}N_7O_{14}$	Annotation propagation
Fe-Amphibactin E	$[M+Fe-2H]^+$	911.429	911.430	1.1	$C_{40}H_{71}N_7O_{13}$	Annotation propagation
Fe-Amphibactin H	$[M+Fe-2H]^+$	913.445	913.445	0	$C_{40}H_{73}N_7O_{13}$	Annotation propagation
Fe-Amphibactin analog 4	$[M+Fe-2H]^+$	915.424	915.424	0	$C_{39}H_{71}N_7O_{14}$	Annotation propagation
Fe-Amphibactin C	$[M+Fe-2H]^+$	927.424	927.424	0	$C_{40}H_{71}N_7O_{14}$	Annotation propagation
Fe-Amphibactin F	$[M+Fe-2H]^+$	929.440	929.439	1.1	$C_{40}H_{73}N_7O_{14}$	Annotation propagation
Fe-Amphibactin I	$[M+Fe-2H]^+$	939.461	939.461	0	$C_{42}H_{75}N_7O_{13}$	Annotation propagation
Fe-Amphibactin analog 5	$[M+Fe-2H]^+$	941.439	941.439	0	$C_{41}H_{73}N_7O_{14}$	Annotation propagation
Fe-Amphibactin analog 6	$[M+Fe-2H]^+$	943.455	943.455	0	$C_{41}H_{75}N_7O_{14}$	Annotation propagation
Fe-Amphibactin G	$[M+Fe-2H]^+$	955.456	955.454	2.1	$C_{42}H_{79}N_7O_{14}$	Annotation propagation

Table S4. ^{1}H (700 MHz) and ^{13}C (176 MHz) NMR data of compound **1** in DMSO- d_6 (J in Hz, δ in ppm)

Residue	No.	δ_C, type	δ_H (J, Hz), multiplicity
N-Ac-*N*-OH-Orn[1]	1	173.8, C	
	2	52.0, CH	4.16, m
	3	29.0, CH_2	1.67 (m), 1.52 (m)
	4	23.3, CH_2	1.51 (overlap)
	5	47.2, CH_2	3.44, m
	6	170.8, C	
	7	20.8, CH_3	1.96, s
	hydroxamate OH		9.69, s
	NH		8.03, d (7.9)
N-Ac-*N*-OH-Orn[2]	1	172.0, C	
	2	52.5, CH	4.31, m
	3	29.9, CH_2	1.60 (m),1.46 (m)
	4	23.3, CH_2	1.49, (overlap)
	5	46.9, CH_2	3.44, m
	6	170.7, C	
	7	20.8, CH_3	1.96, s
	hydroxamate OH		9.69, s
	NH		7.96, d (8.2)
Lipid chain	1	171.4, C	
	2	44.0, CH_2	2.21, d (6.5)
	3	68.1, CH	3.76, m
	4	37.4, CH_2	1.34, m
	5-13	29.6-29.4	1.31-1.20 (overlap)
	14	31.75, CH_2	1.22 (overlap)
	15	29.16, CH_2	1.22 (overlap)
	16	14.43, CH_3	0.84, m
	OH		4.63, d (4.8)

Table S5. Biosynthetic gene clusters present in *Microbulbifer* sp. bacteria analyzed in this study.

Strain	Cluster	Category of predicted cluster	Most similar known cluster	Similarity	Genome size (Mb)
Microbublifer sp. CNSA002 Chromosome I	1	Arylpolyene	APE Vf	40%	5.3
	2	RiPP-like			
	3	Ectoine	Ectoine	100%	
	4	NRPS siderophore	Enantio-pyochelin	20%	
	5	NRPS-like domain			
	6	NIS siderophore			

Table S6. List of annotated *N*-acyl amides detected in this study.

Annotation	Adduct	Theoretical *m/z*	Experimental *m/z*	ppm error	Source	Genus	Samples
Ala/Sarcosine-C10:0	[M + H]+	244.1907	244.191	1.2	GNPS	*Vibrio*, *Photobacterium*, *Bacillus*, coculture	Cn52-H1, Cn52-H1+VASA001, Ofav2-7
Ala/Sarcosine-C12:0	[M + H]+	272.0000	272.000	0.0	GNPS	*Pseudoalteromonas*, *Vibrio*, *Microbulbifer*, *Bacillus*, coculture	Cn52-H1, Cn52-H1+CNSA002, Cn52-H1+VASA001, Cnat2-18.1
Ala/Sarcosine-C16:1	[M + H]+	326.2690	326.270	3.1	GNPS	*Pseudoalteromonas*	Cnat2-18.1
Amylamine-C18:0	[M + H]+	354.373	354.373	0.0	GNPS	*Pseudoalteromonas*, *Vibrio*, *Microbulbifer*, *Photobacterium*, *Bacillus*, coculture	AC-K1-M-019, Cn52-H1, Cn52-H1+AC-K1-M-019, Cn52-H1+CNSA002, Cn52-H1+VASA001, Cnat2-18.1, CNSA002, DL2H-2.2, Ofav2-7, VASA001
Arg-C10:0	[M + H]+	329.2547	329.255	0.9	GNPS	*Vibrio*, *Photobacterium*, *Bacillus*, coculture	Cn52-H1, Cn52-H1+VASA001, Ofav2-7
Arg-C12:0	[M + H]+	357.2860	357.286	0.0	GNPS	*Vibrio*, *Photobacterium*	Cn52-H1, Ofav2-7
Arg-C14:0	[M + H]+	385.3137	385.317	8.6	GNPS	*Pseudoalteromonas*	Cnat2-18.1
Arg-C15:0	[M + H]+	399.3330	399.334	2.5	Annotation propagation	*Pseudoalteromonas*, *Microbulbifer*	Cnat2-18.1, CNSA002
Arg-C15:1	[M + H]+	397.3173	397.318	1.8	Annotation propagation	*Pseudoalteromonas*	Cnat2-18.1
Arg-C16:0	[M + H]+	413.3486	413.349	1.0	GNPS	*Pseudoalteromonas*	Cnat2-18.1
Arg-C16:1	[M + H]+	411.3330	411.333	0.0	GNPS	*Pseudoalteromonas*	Cnat2-18.1
Arg-C17:1	[M + H]+	425.3486	425.348	1.4	GNPS	*Pseudoalteromonas*	Cnat2-18.1
Arg-C6:0	[M + H]+	273.1921	273.192	0.4	GNPS	*Vibrio*	Cn52-H1
Arg-C8:0	[M + H]+	301.0000	301.000	0.0	GNPS	*Vibrio*, *Bacillus*, coculture	Cn52-H1, Cn52-H1+VASA001
Citrulline-C10:0	[M + H]+	330.2387	330.239	0.9	GNPS	*Photobacterium*	Ofav2-7
Citrulline-C12:0	[M + H]+	358.2700	358.271	2.8	GNPS	*Photobacterium*	Ofav2-7
Gly-C12:0	[M + H]+	258.2064	258.207	2.3	GNPS	*Photobacterium*	Ofav2-7
Gly-C16:1	[M + H]+	312.2533	312.254	2.2	GNPS	*Pseudoalteromonas*, *Photobacterium*	Cnat2-18.1, Ofav2-7
His-C14:0	[M + H]+	366.2751	366.275	0.3	GNPS	*Pseudoalteromonas*	Cnat2-18.1
His-C16:1	[M + H]+	392.2908	392.291	0.5	GNPS	*Pseudoalteromonas*	Cnat2-18.1
Kynurenine-C2:0	[M + H]+	251.1026	251.102	2.4	GNPS	*Microbulbifer*	CNSA002
Leu-C10:0	[M + H]+	286.2377	286.238	1.0	GNPS	*Vibrio*, *Photobacterium*, *Bacillus*, coculture	Cn52-H1, Cn52-H1+VASA001, Ofav2-7

**Annotation**	**Adduct**	**Theoretical *m/z***	**Experimental *m/z***	**ppm error**	**Source**	**Genus**	**Samples**
Leu-C13:0	[M + H]+	328.2846	328.285	1.2	Annotation propagation	*Pseudoalteromonas*, *Bacillus*	Cnat2-18.1, VASA001
Leu-C14:0	[M + H]+	342.3003	342.301	2.0	GNPS	*Pseudoalteromonas*	Cnat2-18.1, DL2H-2.2
Leu-C14:1	[M + H]+	340.2846	340.286	4.1	Annotation propagation	*Pseudoalteromonas*	Cnat2-18.1
Leu-C15:0	[M + H]+	356.3159	356.316	0.3	GNPS	*Pseudoalteromonas*, *Vibrio*	Cn52-H1, Cnat2-18.1
Leu-C15:1	[M + H]+	354.3003	354.301	2.0	GNPS	*Pseudoalteromonas*, *Microbulbifer*	Cnat2-18.1, CNSA002
Leu-C17:0	[M + H]+	384.3472	384.348	2.1	Annotation propagation	*Pseudoalteromonas*, *Vibrio*	Cn52-H1, Cnat2-18.1
Leu-C17:1	[M + H]+	382.3316	382.332	1.0	GNPS	*Pseudoalteromonas*, *Microbulbifer*, coculture	AC-K1-M-019, Cn52-H1+AC-K1-M-019, Cnat2-18.1, CNSA002
Leu-C18:1	[M + H]+	396.3472	396.348	2.0	GNPS	*Pseudoalteromonas*, *Microbulbifer*, *Bacillus*, coculture	Cn52-H1+AC-K1-M-019, Cn52-H1+CNSA002, Cn52-H1+VASA001, Cnat2-18.1, CNSA002
Lys-C12:0	[M + H]+	329.2799	329.280	0.3	GNPS	*Photobacterium*	Ofav2-7
Lys-C13:0-OH	[M + H]+	359.2904	359.291	1.7	GNPS	*Vibrio*, *Microbulbifer*	Cn52-H1, CNSA002
Lys-C14:0-OH	[M + H]+	373.3061	373.306	0.3	GNPS	*Microbulbifer*	CNSA002
Lys-C15:0	[M + H]+	371.3268	371.327	0.5	GNPS	*Pseudoalteromonas*, *Microbulbifer*, *Photobacterium*, *Bacillus*	Cnat2-18.1, CNSA002, Ofav2-7, VASA001
Lys-C15:0-OH	[M + H]+	387.3217	387.322	0.8	GNPS	*Pseudoalteromonas*, *Vibrio*, *Microbulbifer*	Cn52-H1,Cnat2-18.1, CNSA002
Lys-C15:1	[M + H]+	369.3112	369.312	2.2	GNPS	*Pseudoalteromonas*, *Vibrio*	Cn52-H1, Cnat2-18.1
Lys-C16:0	[M + H]+	385.3425	385.343	1.3	GNPS	*Pseudoalteromonas*, *Microbulbifer*, *Bacillus*	Cnat2-18.1, CNSA002, VASA001
Lys-C16:1	[M + H]+	383.3268	383.327	0.5	GNPS	*Pseudoalteromonas*, *Vibrio*, *Photobacterium*, *Bacillus*	Cn52-H1, Cnat2-18.1, Ofav2-7, VASA001
Lys-C17:1	[M + H]+	397.3425	397.343	1.3	GNPS	*Pseudoalteromonas*, *Vibrio*, *Bacillus*	Cn52-H1, Cnat2-18.1, VASA001
Lys-C8:0	[M + H]+	273.2173	273.216	4.8	GNPS	*Pseudoalteromonas*, *Vibrio*, *Microbulbifer*, *Photobacterium*, *Bacillus*, coculture	Cn52-H1, Cn52-H1+CNSA002, Cn52-H1+VASA001, Cnat2-18.1, Ofav2-7
Met-C14:0	[M + H]+	360.2567	360.257	0.8	Annotation propagation	*Pseudoalteromonas*, *Microbulbifer*	Cnat2-18.1, CNSA002, DL2H-2.2
Met-C15:1	[M + H]+	372.2567	372.258	3.5	Annotation propagation	*Pseudoalteromonas*, *Microbulbifer*	Cnat2-18.1, CNSA002
Met-C16:1	[M + H]+	386.2723	386.272	0.8	GNPS	*Pseudoalteromonas*, *Microbulbifer*	Cnat2-18.1, CNSA002

Annotation	Adduct	Theoretical *m/z*	Experimental *m/z*	ppm error	Source	Genus	Samples
Met-C17:1	[M + H]+	400.288	400.289	2.5	GNPS	*Pseudoalteromonas*, *Microbulbifer*, *Bacillus*	Cnat2-18.1, CNSA002, VASA001
Orn-C14:0-OH	[M + H]+	359.2904	359.291	1.7	Annotation propagation	*Vibrio*	Cn52-H1
Orn-C15:0-OH	[M + H]+	373.3061	373.306	0.3	Annotation propagation	*Vibrio*, *Microbulbifer*	Cn52-H1, CNSA002
Orn-C16:0-OH	[M + H]+	387.3217	387.322	0.8	Annotation propagation	*Pseudoalteromonas*, *Vibrio*, *Microbulbifer*	Cn52-H1, Cnat2-18.1, CNSA002
Orn-C16:1-OH	[M + H]+	385.3061	385.306	0.3	Annotation propagation	*Vibrio*	Cn52-H1
Orn-C17:1-OH	[M + H]+	399.3217	399.323	3.3	Annotation propagation	*Pseudoalteromonas*, *Microbulbifer*	Cnat2-18.1, CNSA002
Orn-C18:1-OH	[M + H]+	413.3374	413.338	1.5	Annotation propagation	*Pseudoalteromonas*, *Vibrio*, *Microbulbifer*	Cn52-H1, Cnat2-18.1, CNSA002
Phe-C10:0	[M + H]+	320.2220	320.222	0.0	GNPS	*Vibrio*, *Photobacterium*, *Bacillus*, coculture	Cn52-H1, Cn52-H1+VASA001, Ofav2-7
Phe-C12:0	[M + H]+	348.2533	348.254	2.0	GNPS	*Pseudoalteromonas*, *Vibrio*, *Photobacterium*	Cn52-H1, Cnat2-18.1, Ofav2-7
Phe-C14:0	[M + H]+	376.2846	376.285	1.1	GNPS	*Pseudoalteromonas*, *Vibrio*, *Microbulbifer*, coculture	Cn52-H1, Cn52-H1+CNSA002, Cnat2-18.1, CNSA002
Phe-C14:1	[M + H]+	374.2690	374.269	0.0	GNPS	*Pseudoalteromonas*	Cnat2-18.1
Phe-C15:0	[M + H]+	390.3003	390.300	0.8	GNPS	*Pseudoalteromonas*, *Vibrio*, *Microbulbifer*, *Photobacterium*, *Bacillus*	Cn52-H1, Cnat2-18.1, CNSA002, Ofav2-7, VASA001
Phe-C15:1	[M + H]+	388.2846	388.287	6.2	GNPS	*Pseudoalteromonas*, *Microbulbifer*	Cnat2-18.1, CNSA002
Phe-C16:0	[M + H]+	404.3159	404.316	0.2	GNPS	*Pseudoalteromonas*, *Microbulbifer*, *Photobacterium*, coculture	Cn52-H1+CNSA002, Cnat2-18.1, CNSA002, Ofav2-7
Phe-C16:1	[M + H]+	402.3003	402.300	0.7	GNPS	*Pseudoalteromonas*, *Microbulbifer*, *Photobacterium*, *Bacillus*, coculture	Cn52-H1+AC-K1-M-019, Cn52-H1+CNSA002, Cnat2-18.1, CNSA002, DL2H-2.2, Ofav2-7, VASA001
Phe-C17:1	[M + H]+	416.3159	416.317	2.6	GNPS	*Pseudoalteromonas*, *Microbulbifer*, *Bacillus*, coculture	Cn52-H1+CNSA002, Cnat2-18.1, CNSA002, VASA001
Phe-C18:1	[M + H]+	430.3316	430.332	0.9	GNPS	*Pseudoalteromonas*	Cnat2-18.1
Phe-C4:0	[M + H]+	236.1281	236.128	0.4	GNPS	*Pseudoalteromonas*, *Vibrio*, *Photobacterium*	Cn52-H1, Cnat2-18.1, Ofav2-7
Thr/Hse-C11:0-OH	[M + H]+	304.2118	304.211	2.6	Annotation propagation	*Photobacterium*	Ofav2-7
Thr/Hse-C12:0-OH	[M + H]+	318.2275	318.227	1.6	GNPS	*Photobacterium*	Ofav2-7

Annotation	Adduct	Theoretical *m/z*	Experimental *m/z*	ppm error	Source	Genus	Samples
Thr/Hse-C13:0	[M + H]+	314.2326	314.232	1.9	Annotation propagation	*Photobacterium*	Ofav2-7
Thr/Hse-C13:0-OH	[M + H]+	332.2431	332.243	0.3	Annotation propagation	*Photobacterium*	Ofav2-7
Thr/Hse-C14:0-OH	[M + H]+	346.2588	346.260	3.5	GNPS	*Photobacterium*	Ofav2-7
Thr/Hse-C14:1-OH	[M + H]+	344.2431	344.244	2.6	Annotation propagation	*Pseudoalteromonas*, *Photobacterium*, coculture	AC-K1-M-019, Cn52-H1+AC-K1-M-019, Ofav2-7
Thr/Hse-C16:1	[M + H]+	356.2795	356.280	1.4	GNPS	*Vibrio*	Cn52-H1
Trp-C14:0	[M + H]+	415.2955	415.295	1.2	GNPS	*Pseudoalteromonas*	Cnat2-18.1
Trp-C15:1	[M + H]+	427.2955	427.296	1.2	GNPS	*Pseudoalteromonas*	Cnat2-18.1
Trp-C16:1	[M + H]+	441.3112	441.311	0.5	GNPS	*Pseudoalteromonas*	Cnat2-18.1
Trp-C8:0	[M + H]+	331.2016	331.202	1.2	GNPS	*Vibrio*, *Photobacterium*	Cn52-H1, Ofav2-7
Tryptamine-C10:0	[M + H]+	315.2431	315.244	2.9	Annotation propagation	*Vibrio*, *Microbulbifer*, coculture	Cn52-H1, Cn52-H1+CNSA002
Tryptamine-C11:0	[M + H]+	329.2587	329.259	0.9	Annotation propagation	*Vibrio*, *Microbulbifer*, *Bacillus*, coculture	Cn52-H1, Cn52-H1+CNSA002, Cn52-H1+VASA001
Tryptamine-C12:0	[M + H]+	343.2744	343.275	1.7	GNPS	*Vibrio*, *Microbulbifer*, *Bacillus*, coculture	Cn52-H1, Cn52-H1+CNSA002, Cn52-H1+VASA001
Tryptamine-C14:0	[M + H]+	371.3057	371.306	0.8	GNPS	*Vibrio*, *Bacillus*, coculture	Cn52-H1, Cn52-H1+VASA001
Tryptamine-C15:0	[M + H]+	385.3213	385.323	4.4	GNPS	*Vibrio*, *Bacillus*, coculture	Cn52-H1, Cn52-H1+VASA001
Tryptamine-C16:1	[M + H]+	397.3213	397.322	1.8	GNPS	*Pseudoalteromonas*, *Vibrio*, *Microbulbifer*, *Photobacterium*, *Bacillus*, coculture	Cn52-H1, Cn52-H1+AC-K1-M-019, Cn52-H1+CNSA002, Cn52-H1+VASA001, Cnat2-18.1, DL2H-2.2, Ofav2-7
Tryptamine-C18:1	[M + H]+	425.3526	425.353	0.9	GNPS	*Vibrio*, *Microbulbifer*, *Photobacterium*, *Bacillus*, coculture	Cn52-H1, Cn52-H1+CNSA002, Cn52-H1+VASA001, Ofav2-7
Tryptamine-C2:0	[M + H]+	203.1179	203.118	0.5	GNPS	*Pseudoalteromonas*, *Vibrio*, *Photobacterium*, *Bacillus*, coculture	Cn52-H1, Cn52-H1+AC-K1-M-019, Cn52-H1+VASA001, Ofav2-7
Tryptamine-C5:0	[M + H]+	245.1648	245.165	0.8	Annotation propagation	*Pseudoalteromonas*, *Vibrio*, *Microbulbifer*, *Photobacterium*, *Bacillus*, coculture	Cn52-H1+AC-K1-M-019,Cn52-H1, Cn52-H1+CNSA002, Cn52-H1+VASA001, Ofav2-7

**Annotation**	**Adduct**	**Theoretical *m/z***	**Experimental *m/z***	**ppm error**	**Source**	**Genus**	**Samples**
Tryptamine-C6:1	[M + H]+	257.1648	257.165	0.8	Annotation propagation	*Photobacterium*	Ofav2-7
Tyr-C10:0	[M + H]+	336.2169	336.217	0.3	GNPS	*Vibrio*, *Photobacterium*	Cn52-H1, Ofav2-7
Tyr-C12:1	[M + H]+	362.2326	362.233	1.1	GNPS	*Pseudoalteromonas*, *Vibrio*, *Bacillus*, coculture	Cn52-H1,Cn52-H1+AC-K1-M-019, Cn52-H1+VASA001, VASA001
Tyr-C14:0	[M + H]+	392.2795	392.282	6.4	GNPS	*Pseudoalteromonas*	Cnat2-18.1
Tyr-C15:1	[M + H]+	404.2795	404.282	6.2	GNPS	*Pseudoalteromonas*, coculture	AC-K1-M-019, Cn52-H1+AC-K1-M-019, Cnat2-18.1
Tyr-C16:1	[M + H]+	418.2952	418.296	1.9	GNPS	*Pseudoalteromonas*	AC-K1-M-019
Tyr-C17:1	[M + H]+	432.3108	432.311	0.5	GNPS	*Pseudoalteromonas*	AC-K1-M-019
Tyr-C8:0	[M + H]+	308.1856	308.185	1.9	GNPS	*Photobacterium*	Ofav2-7
